# NifA is the master regulator of both nitrogenase systems in *Rhodobacter capsulatus*

**DOI:** 10.1002/mbo3.921

**Published:** 2019-08-22

**Authors:** Lisa Demtröder, Yvonne Pfänder, Sina Schäkermann, Julia Elisabeth Bandow, Bernd Masepohl

**Affiliations:** ^1^ Microbial Biology, Faculty of Biology and Biotechnology Ruhr University Bochum Bochum Germany; ^2^ Applied Microbiology, Faculty of Biology and Biotechnology Ruhr University Bochum Bochum Germany

**Keywords:** AnfA regulon, Fe‐nitrogenase, Mo‐nitrogenase, NifA regulon, *Rhodobacter*

## Abstract

*Rhodobacter capsulatus* fixes atmospheric nitrogen (N_2_) by a molybdenum (Mo)‐nitrogenase and a Mo‐free iron (Fe)‐nitrogenase, whose production is induced or repressed by Mo, respectively. At low nanomolar Mo concentrations, both isoenzymes are synthesized and contribute to nitrogen fixation. Here we examined the regulatory interplay of the central transcriptional activators NifA and AnfA by proteome profiling. As expected from earlier studies, synthesis of the structural proteins of Mo‐nitrogenase (NifHDK) and Fe‐nitrogenase (AnfHDGK) required NifA and AnfA, respectively, both of which depend on the alternative sigma factor RpoN to activate expression of their target genes. Unexpectedly, NifA was found to be essential for the synthesis of Fe‐nitrogenase, electron supply to both nitrogenases, biosynthesis of their cofactors, and production of RpoN. Apparently, RpoN is the only NifA‐dependent factor required for target gene activation by AnfA, since plasmid‐borne *rpoN* restored *anfH* transcription in a NifA‐deficient strain. However, plasmid‐borne *rpoN* did not restore Fe‐nitrogenase activity in this strain. Taken together, NifA requirement for synthesis and activity of both nitrogenases suggests that Fe‐nitrogenase functions as a complementary nitrogenase rather than an alternative isoenzyme in *R*.* capsulatus*.

## Graphical abstract

In *Rhodobacter capsulatus*, biological nitrogen fixation is catalyzed by the molybdenum nitrogenase and a Mo‐free iron‐only nitrogenase. Here, we show that NifA controls synthesis of both nitrogenases by controlling synthesis of the sigma factor RpoN.

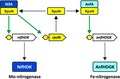

## 1 INTRODUCTION

Biological nitrogen fixation, the enzymatic reduction of highly abundant but chemically inert molecular nitrogen, N_2_, from air to bioavailable ammonia, NH_3_, is exclusively performed by diazotrophic bacteria and archaea, but not by eukaryotes. N_2_ reduction is catalyzed by three isoenzymes, namely molybdenum (Mo)‐nitrogenase, vanadium (V)‐nitrogenase, and iron‐only (Fe)‐nitrogenase (Loveless & Bishop, 1999; McGlynn, Boyd, Peters, & Orphan, 2012; McRose, Zhang, Kraepiel, & Morel, 2017; Thiel & Pratte, 2014). While all diazotrophs have Mo‐nitrogenase, only few are capable of synthesizing one or both Mo‐free nitrogenases. V‐ and Fe‐nitrogenases are less efficient than Mo‐nitrogenases in terms of ATP consumption per N_2_ reduced (Eady, 1996, 2003; Lee, Hu, & Ribbe, 2009; Schneider, Gollan, Dröttboom, Selsemeier‐Voigt, & Müller, 1997; Seefeldt, Yang, Duval, & Dean, 2013) that is why molybdate represses synthesis of Mo‐free nitrogenases in many diazotrophs making Mo‐nitrogenase the preferred isoenzyme (Demtröder, Narberhaus, & Masepohl, 2019; Hamilton et al., 2011; Kutsche, Leimkühler, Angermüller, & Klipp, 1996; Thiel & Pratte, 2014; Wiethaus, Wirsing, Narberhaus, & Masepohl, 2006). Mo limitation and low temperature, however, favor nitrogen fixation by Mo‐free nitrogenases (Miller & Eady, 1988). In addition, Mo‐free nitrogenases have recently been shown to be active in unexpected environments that are not obviously depleted in Mo (Darnajoux et al., 2017; McRose et al., 2017).

All diazotrophs have a core set of nitrogen fixation (*nif*) genes essential for the biosynthesis of Mo‐nitrogenase (Curatti & Rubio, 2014; Dos Santos, Fang, Mason, Setubal, & Dixon, 2012; Hu & Ribbe, 2011; Wang et al., 2013). These are the structural genes of Mo‐nitrogenase (*nifHDK*), genes involved in iron–sulfur cluster formation (*nifUS*), and genes required for the biosynthesis of the iron–molybdenum cofactor, FeMoco (*nifB*, *nifEN*, *nifV*). In addition, some diazotrophs have the structural genes of V‐nitrogenase (*vnfH*, *vnfDGK*) or Fe‐nitrogenase (*anfHDGK*) or both. In *Azotobacter vinelandii*, activity of the V‐ and Fe‐nitrogenases depends on the *nifUS*, *nifB*, and *nifV* genes, reflecting common biosynthetic pathways and structural similarity of the cofactors of Mo‐nitrogenase (FeMoco), V‐nitrogenase (FeVco), and Fe‐nitrogenase (FeFeco) (Drummond, Walmsley, & Kennedy, 1996; Hamilton et al., 2011; Hu & Ribbe, 2016; Kennedy & Dean, 1992; Sippel & Einsle, 2017; Yang, Xie, Wang, Dixon, & Wang, 2014). Besides the core set of *nif* genes, diazotrophs have species‐specific *nif* genes involved in electron transfer to nitrogenase (*nifF*, *fdxN*, *rnfABCDGEH*, *fixABCX*), adaptation to environmental niches, and in case of symbiotic diazotrophs, interaction with their eukaryotic host (Boyd, Costas, Hamilton, Mus, & Peters, 2015; Dos Santos et al., 2012; Oldroyd, 2013; Poudel et al., 2018).

Effective growth with N_2_ as sole nitrogen source requires huge amounts of nitrogenase, which can make up 10% of the total soluble proteome, and reduction of one N_2_ molecule by Mo‐nitrogenase consumes at least 16 ATP molecules (Dingler, Kuhla, Wassink, & Oelze, 1988; Eady, 1996, 2003; Hamilton et al., 2011; Hoffmann et al., 2016; Lee et al., 2009; Schneider et al., 1997; Seefeldt et al., 2013; Sippel & Einsle, 2017). As compared to Mo‐nitrogenase, Mo‐free nitrogenases consume even more ATP during nitrogen fixation; for example, 40 ATP per N_2_ reduced have been determined for *Azotobacter chroococcum* V‐nitrogenase (Eady, 2003; Sippel & Einsle, 2017). In any case, nitrogen fixation is a costly process, and consequently, diazotrophs synthesize nitrogenases only when ammonium is limiting (Bueno Batista & Dixon, 2019; Erkal et al., 2019; Fischer, 1994; Herrero & Flores, 2019; Kessler & Leigh, 1999; Martinez‐Argudo, Little, Shearer, Johnson, & Dixon, 2004; Wang et al., 2013).

NifA, VnfA, and AnfA are the central nitrogen fixation regulators that activate transcription of all the other *nif*, *vnf*, and *anf* genes, respectively, in proteobacterial diazotrophs (Dixon & Kahn, 2004; Drummond et al., 1996; Fischer, 1994; Hamilton et al., 2011; Heiniger, Oda, Samanta, & Harwood, 2012; Hübner, Masepohl, Klipp, & Bickle, 1993; Joerger, Jacobson, & Bishop, 1989; Kutsche et al., 1996; Mus, Alleman, Pence, Seefeldt, & Peters, 2018; Oda et al., 2005; Oliveira et al., 2012; Sarkar & Reinhold‐Hurek, 2014; Souza, Pedrosa, Rigo, Machado, & Yates, 2000; Zhang, Pohlmann, Ludden, & Roberts, 2000; Zou et al., 2008). A common factor required for the activation of target promoters by NifA, VnfA, and AnfA is the alternative sigma factor RpoN (Bush & Dixon, 2012; Fischer, 1994; Merrick, 1993).

**Figure 1:**
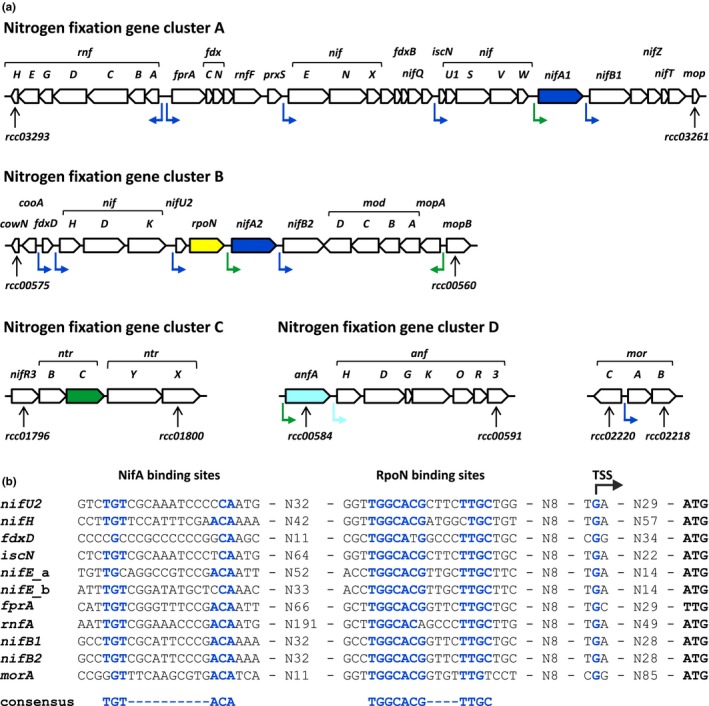
Nitrogen fixation genes and promoters in *Rhodobacter capsulatus*. (a) Organization of nitrogen fixation genes. Most nitrogen fixation genes belong to one of four chromosomal clusters, A–D (Masepohl & Klipp, 1996; Schüddekopf et al., 1993). Known or presumed promoters activated by NifA (upstream of *rnfA*, *fprA*, *nifE*, *nifU1*, *nifB1*, *fdxD*, *nifH*, *nifU2*, *nifB2*, and *morA*), AnfA (upstream of *anfH*), and NtrC (upstream of *nifA1*, *nifA2*, *mopA*, and *anfA*) are marked by bent arrows (Cullen et al., 1994; Foster‐Hartnett & Kranz, 1992; Preker, Hübner, Schmehl, Klipp, & Bickle, 1992; Wiethaus et al., 2006; Willison, Pierrard, & Hübner, 1993). (b) Comparison of nitrogen fixation promoters. Conserved nucleotides in the presumed binding sites of NifA and RpoN are highlighted in blue. For consensus sequences, see Buck, Miller, Drummond, & Dixon, 1986; Morett & Buck, 1988; Morett & Buck, 1989. The *nifE* promoter encompasses two possible NifA binding sites (*nifE*_a*, nifE*_b). Transcription start sites (TSS) have been experimentally determined for the *nifU2*, *nifH*, and *fdxD* promoters (Preker et al., 1992; Willison et al., 1993). The number of nucleotides (N) between different cis‐regulatory elements (NifA and RpoN binding sites, TSS, and ATG start codon) is indicated

*Rhodobacter capsulatus* is a photosynthetic alphaproteobacterium capable of synthesizing Mo‐ and Fe‐nitrogenases (Schneider, Müller, Schramm, & Klipp, 1991; Schüddekopf, Hennecke, Liese, Kutsche, & Klipp, 1993). In this model bacterium, most nitrogen fixation genes are clustered in four chromosomal regions, A–D (Figure 1) (Masepohl & Klipp, 1996; Schüddekopf et al., 1993). *R*.* capsulatus* contains two almost identical and functionally redundant *nifA* copies, *nifA1* and *nifA2*, while other proteobacterial diazotrophs typically have only one *nifA* copy (Fischer, 1994; Masepohl, Klipp, & Pühler, 1988; Sullivan, Brown, & Ronson, 2013). Upon ammonium depletion, expression of both *nifA* genes is equally activated by NtrC, but *nifA2* expression is further enhanced by RegA (Elsen, Dischert, Colbeau, & Bauer, 2000). RegA forms part of the redox‐responding RegBA two‐component system regulating photosynthesis, carbon dioxide assimilation, hydrogen oxidation, and nitrogen fixation. RegA acts as a coactivator of *nifA2* expression, but is incapable of activating *nifA2* transcription in the absence of NtrC. In addition to *nifA1* and *nifA2*, NtrC activates expression of *anfA* (Cullen, Bowman, Hartnett, Reilly, & Kranz, 1998; Foster‐Hartnett, Cullen, Monika, & Kranz, 1994; Hübner et al., 1993). In contrast to NtrC proteins from other bacteria, which cooperate with RpoN, *R*.* capsulatus* NtrC acts in concert with the housekeeping sigma factor RpoD (Bowman & Kranz, 1998). Under molybdate–replete conditions, either of the two ModE‐like regulators, MopA or MopB, represses *anfA* transcription (Kutsche et al., 1996; Wang, Angermüller, & Klipp, 1993; Wiethaus et al., 2006).

To improve our understanding of the regulatory interplay of NifA and AnfA in *R*.* capsulatus*, we took advantage of its ability to simultaneously synthesize Mo‐ and Fe‐nitrogenase when grown under molybdate‐limiting conditions (Hoffmann et al., 2016). Proteome profiling identified the putative periplasmic molybdate‐binding protein MorA as a previously unrecognized NifA‐controlled protein. A closer inspection of the NifA and AnfA regulons revealed that NifA is crucial for *rpoN* expression and, in this way, indirectly influences AnfA‐dependent activation of Fe‐nitrogenase genes.

## 2 MATERIALS AND METHODS

### 2.1 Strains, plasmids, and growth conditions

The bacterial strains and plasmids used in this study are shown in Table A1 (Appendix 1). *R*.* capsulatus* minimal medium V (RCV) contained 30 mM DL‐malic acid, 10 mM potassium phosphate buffer, 0.8 mM MgSO_4_, 0.7 mM CaCl_2_, 50 µM EDTA, 45 µM H_3_BO_3_, 40 µM FeSO_4_, 9.5 µM MnSO_4_, 3 µM thiamine hydrochloride, 0.85 µM ZnSO_4_, 0.15 µM Cu(NO_3_)_2_ with pH adjusted to 6.8 before autoclaving. In this medium, a fixed nitrogen source and molybdate have been omitted. Traces of Mo arising from impurities of the chemicals used support residual Mo‐nitrogenase activity in the wild type, but not in a strain lacking the high‐affinity molybdate transporter ModABC (Gisin et al., 2010). Hence, this medium is Mo‐limited (−Mo), but not completely Mo‐free (Hoffmann et al., 2016). When required, 10 µM Na_2_MoO_4_, 10 mM L‐serine, or 10 mM (NH_4_)_2_SO_4_ were added. To determine diazotrophic growth, 3‐ml cultures were placed in screw‐capped 17‐ml Hungate tubes prior to exchanging the headspace for N_2_ gas (as sole nitrogen source) and incubation in the light.

### 2.2 Proteome profiling of *Rhodobacter capsulatus* strains lacking nitrogen fixation regulators

To determine proteome profiles of the wild‐type (B10S), Δ*nifA1‐A2* (YP202‐YP203), Δ*anfA* (KS94A), and Δ*mopAB* (R423CI) strains, cultures were phototrophically grown under nitrogenase‐derepressing conditions in RCV minimal medium with or without the addition of 10 µM Na_2_MoO_4_. Media contained serine, which does not repress nitrogen fixation, as sole nitrogen source. Protein preparation, tryptic digestion, spiking with PhosB peptides, mass spectrometry, and data processing were carried out essentially as described earlier (Hoffmann et al., 2016). Up‐ and downregulated proteins were selected using a confidence interval of 95% and p‐values below 0.05. For Δ*nifA1‐A2*, Δ*anfA*, and Δ*mopAB* strains, proteins with log_2_ ratios below −0.82, −1.19, or −1.58, and above 0.92, 0.75, or 1.20 were considered significantly down‐ or upregulated compared to the wild type upon −Mo conditions, respectively. log_2_ ratios for Δ*nifA1‐A2*, Δ*anfA*, and Δ*mopAB* strains below −0.88, −0.80, or −1.10, and above 1.22, 0.97, or 1.31 were considered significantly down‐ or upregulated compared to the wild type upon +Mo conditions, respectively. Proteins present in all three biological replicates of one condition and missing in all biological replicates of the other condition were considered as unique for the first condition.

### 2.3 Construction of *Rhodobacter capsulatus lacZ* reporter strains and β‐galactosidase assays

Transcriptional fusions between selected nitrogen fixation (*nif*) genes and the promoterless *Escherichia coli lacZ* gene were generated essentially as described earlier (Hoffmann et al., 2016). Briefly, appropriate primer pairs were used to PCR‐amplify genes of interest, thereby adding a HindIII or a BamHI site immediately downstream of the stop codon. After blunt‐end cloning of these DNA fragments into the SmaI site of the narrow‐host‐range plasmid pYP168 (Hoffmann et al., 2016), the *lac*TeT cassette (carrying the promoterless *lacZ* gene, a tetracycline resistance gene, and a conjugational transfer origin) from plasmid pYP5 (Gisin et al., 2010) was inserted into the HindIII or BamHI site. The resulting reporter plasmids were conjugationally transferred into *R*.* capsulatus*. Selection for tetracycline resistance indicated plasmid integration into the chromosome by single recombination events, placing the *lacZ* reporters under the control of the respective *nif* promoters. The design of the *lacZ* reporter strains is not supposed to interrupt the expression of the respective *nif* gene. The resulting *R*.* capsulatus* reporter strains were grown phototrophically in RCV medium with 10 mM serine until the late logarithmic phase prior to determination of LacZ (β‐galactosidase) activity.

### 2.4 Nitrogenase activity assays and AnfH detection by Western analysis

To determine in vivo nitrogenase activity, *R*.* capsulatus* wild‐type and mutant strains were phototrophically grown in RCV minimal medium with 10 mM serine (no Mo added) in screw‐capped Hungate tubes (headspace flushed with argon) until the late logarithmic phase. Nitrogenase activity was determined by the acetylene reduction assay as described earlier (Wang et al., 1993).

AnfH accumulation was determined by Western analysis. For this purpose, cell‐free protein was isolated from the cultures used in the acetylene reduction assay. Western analysis was carried out as described earlier using an antiserum raised against *R*.* capsulatus* AnfH protein (Masepohl, Krey, & Klipp, 1993).

## 3 RESULTS

### 3.1 Proteome profiling of *Rhodobacter capsulatus* strains lacking NifA or AnfA or MopAB

**Figure A1:**
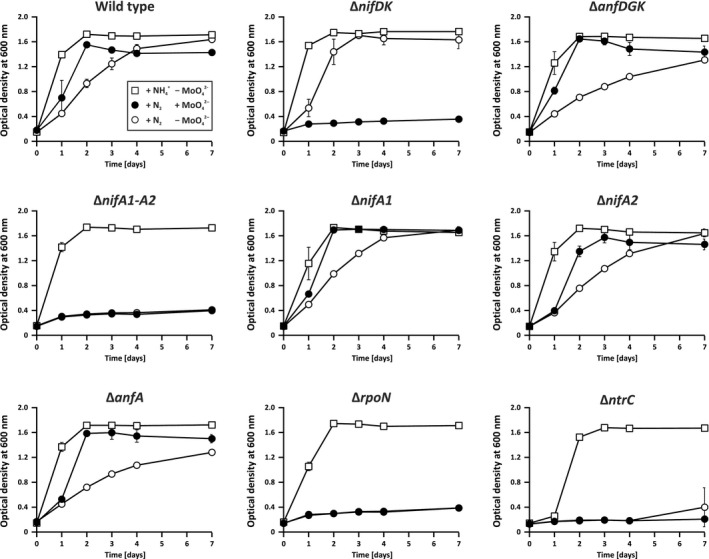
Diazotrophic growth of *Rhodobacter capsulatus* wild‐type and mutant strains. *R*.* capsulatus* strains were grown in RCV minimal medium without (open circles) or with 10 µM molybdate (filled circles) under a pure N_2_ atmosphere (no fixed nitrogen source added). Growth in RCV medium containing 10 mM ammonium (open squares) served as control. The strains used were as follows: wild type (B10S), Δ*nifDK* (BS85), Δ*anfDGK* (YP243), Δ*nifA1‐A2* (YP202‐YP203), Δ*nifA1* (YP203), Δ*nifA2* (YP202), Δ*anfA* (KS94A), Δ*rpoN* (YP201), and Δ*ntrC* (PBK2). Diazotrophic growth of the Δ*anfDGK* and Δ*anfA* strains in −MoO_4_^2−^ medium is attributable to traces of Mo arising from impurities in the chemicals used. The data represent the means and standard deviations of the results of at least three independent measurements

Mo‐nitrogenase levels increase with increasing molybdate concentrations in *R*.* capsulatus*; however, significant quantities are produced even under severe Mo limitation as is the case in a strain lacking the high‐affinity molybdate transporter ModABC (Gisin et al., 2010; Hoffmann et al., 2016). In contrast, the Mo‐free Fe‐nitrogenase is exclusively synthesized under Mo‐limiting conditions. Both nitrogenases are synthesized simultaneously as long as Mo concentrations are in the low nanomolar range. To better understand how the production of two complementary nitrogenases with different catalytic efficiencies is coordinated, we examined the proteomes of *R*.* capsulatus* strains lacking NifA (Δ*nifA1‐A2*) or AnfA (Δ*anfA*) or MopA and MopB (Δ*mopAB*). Due to the redundant functions of NifA1 and NifA2 in *nif* gene activation, and MopA and MopB in *anfA* repression, the Δ*nifA1‐A2* and Δ*mopAB* double mutants were used (Figure A1 in Appendix 2; Masepohl et al., 1988; Paschen, Drepper, Masepohl, & Klipp, 2001; Wiethaus et al., 2006).

In these mutants, the regulatory genes are disrupted by antibiotic resistance cassettes (Table A1 in Appendix 1). Since *nifA1*, *nifA2*, and *anfA* form monocistronic operons, we do not expect polar effects on downstream genes by cassette insertion. In the Δ*mopAB* strain, however, the nearby *mopA* and *mopB* genes are replaced by a gentamicin cassette, which drives expression of the *modABC* genes belonging to the *mopA*‐*modABC* operon (Figure 1a; Wiethaus et al., 2006). Table 1 shows low but Mo‐independent ModA production in the Δ*mopAB* strain. In contrast to a strain lacking the ModABC transporter, the Δ*mopAB* strain still exhibits Mo‐nitrogenase activity under Mo‐limiting conditions (Wang et al., 1993).

**Table 1:** Levels of nitrogen fixation proteins in *Rhodobacter capsulatus* wild‐type and regulatory mutant strains

Gene ID	Protein	Description or function	Level of protein (fmol)^a^							
			Wild type		Δ*nifA1*‐*A2*		Δ*anfA*		Δ*mopAB*	
			−Mo	+Mo	−Mo	+Mo	−Mo	+Mo	−Mo	+Mo
Proteins encoded by nitrogen fixation gene cluster A										
Rcc03261	Mop	Molybdate storage	**161.0**	*3*.*0*	86.0	*16*.*2*	**165.0**	**18.5**	**33.1**	NF
Rcc03262		SIR2 family protein	**7.3**	6.6	NF	NF	4.6	**6.3**	**11.5**	**5.0**
Rcc03263	NifT	FeMoco biosynthesis	17.8	**11.5**	NF	NF	**9.9**	*7*.*3*	**22.0**	8.0
Rcc03268	NifW	NifDK maturation	*2*.*9*	NF	NF	NF	NF	NF	**4.4**	NF
Rcc03270	NifS	Cysteine desulfurase	**2.1**	**1.4**	NF	NF	NF	NF	**2.2**	**1.8**
Rcc03271	NifU1	FeS cluster biosynthesis	*13*.*0*	*9*.*0*	NF	NF	*1*.*8*	*7*.*3*	**12.0**	*7*.*1*
Rcc03272	IscN	FeS cluster biosynthesis	**8.0**	NF	NF	NF	**4.1**	*2*.*2*	**9.0**	*3*.*4*
Rcc03275	FdxB	Ferredoxin III	**15.1**	**6.4**	NF	NF	**6.3**	**7.1**	**10.1**	3.7
Rcc03276		Rop family protein	*12*.*5*	NF	NF	NF	9.1	NF	*3*.*6*	*3*.*5*
Rcc03277		DUF269	**18.7**	**14.2**	NF	NF	**15.9**	**17.0**	**20.3**	**9.2**
Rcc03278	NifX	FeMoco biosynthesis	**35.0**	**25.0**	NF	NF	**24.4**	**21.6**	**36.5**	**15.0**
Rcc03279	NifN	FeMoco biosynthesis	**3.5**	1.9	NF	NF	**2.4**	NF	5.6	NF
Rcc03280	NifE	FeMoco biosynthesis	**4.6**	**2.0**	NF	NF	3.1	**2.0**	**6.0**	*1*.*5*
Rcc03281	PrxS	Peroxiredoxin	5.2	3.7	**2.5**	**2.0**	5.8	**5.7**	**16.6**	11.1
Rcc03285	FdxC	Ferredoxin IV	**19.5**	**9.9**	NF	NF	**10.7**	**10.2**	**14.7**	**7.7**
Rcc03286	FprA	Flavorubredoxin	*3*.*5*	*2*.*9*	NF	NF	NF	NF	**5.8**	NF
Rcc03291	RnfG	Electron transport	**5.8**	**2.7**	NF	NF	*1*.*3*	1.2	**3.1**	*1*.*2*
Rcc03288	RnfB	Electron transport	*2*.*3*	NF	NF	NF	NF	NF	NF	NF
Proteins encoded by nitrogen fixation gene cluster B										
Rcc00560	MopB	Mo‐responsive regulator	1.8	2.5	*0*.*8*	**1.6**	0.7	1.8	NF	NF
Rcc00561	MopA	Mo‐responsive regulator	**15.9**	**3.1**	**6.2**	NF	13.7	**2.9**	NF	NF
Rcc00562	ModA	Molybdate transporter	**163.6**	15.0	90.8	*7*.*5*	648.2	57.9	**5.6**	**8.2**
Rcc00565	ModD	NAD biosynthesis	**3.3**	NF	**2.8**	NF	**4.3**	*1*.*5*	NF	NF
Rcc00570	NifK	Mo‐nitrogenase	**108.8**	**277.3**	NF	*1*.*5*	97.8	**262.3**	**261.5**	**170.8**
Rcc00571	NifD	Mo‐nitrogenase	**62.3**	**234.8**	*8*.*3*	NF	53.9	**216.9**	**171.9**	**130.4**
Rcc00572	NifH	Mo‐nitrogenase	**325.3**	**404.5**	*13*.*2*	*1*.*5*	**366.8**	**434.0**	**390.0**	**235.0**
Rcc00573	FdxD	Shetna ferredoxin	**17.1**	**22.9**	NF	NF	**39.0**	**19.0**	**6.4**	*15*.*0*
Proteins encoded by nitrogen fixation gene cluster C										
Rcc01798	NtrC	2‐component regulator	*3*.*4*	**2.7**	4.5	**4.5**	2.4	**2.1**	*1*.*8*	*1*.*5*
Rcc01800	NtrX	2‐component regulator	2.5	1.6	**2.1**	**2.9**	1.6	**2.0**	**1.5**	**2.1**
Proteins encoded by nitrogen fixation gene cluster D										
Rcc00585	AnfH	Fe‐nitrogenase	**329.7**	1.2	NF	NF	*0*.*4*	*1*.*2*	**466.5**	**53.1**
Rcc00586	AnfD	Fe‐nitrogenase	**37.4**	NF	NF	NF	NF	*3*.*2*	**79.6**	*1*.*8*
Rcc00587	AnfG	Fe‐nitrogenase	**12.3**	NF	NF	NF	NF	NF	**11.2**	NF
Rcc00588	AnfK	Fe‐nitrogenase	**76.3**	2.5	NF	NF	NF	*3*.*2*	**113.1**	**16.5**
Rcc00589	AnfO	Fe‐nitrogenase accessory	2.9	NF	NF	NF	NF	NF	**12.5**	NF
Rcc00591	Anf3	Fe‐nitrogenase accessory	**34.7**	*2*.*9*	NF	NF	NF	*2*.*6*	**48.6**	**6.4**
Proteins encoded by genes apart from nitrogen fixation gene clusters A–D										
Rcc02219	MorA	ModA‐like protein	**19.5**	NF	NF	NF	**40.5**	NF	**45.9**	**28.8**
Rcc02220	MorC	ModC‐like protein	*1*.*6*	NF	NF	NF	NF	NF	NF	NF

Abbreviation: ID, identifier; NF, protein never found in any of the replicates.

a Values with standard deviations of <25% are in bold. Values for proteins identified in one replicate only are in italics.

To achieve comparable growth of *R*.* capsulatus* wild‐type and mutant strains, a fixed nitrogen source, serine, was added to the RCV minimal medium. In contrast to ammonium, serine does not repress nitrogen fixation (Hoffmann et al., 2016; Klipp, Masepohl, & Pühler, 1988). To achieve Mo‐limiting and Mo‐replete conditions, *R*.* capsulatus* strains were grown without (−Mo) or with 10 micromolar molybdate (+Mo), respectively, prior to protein isolation, processing, and mass‐spectrometric quantification as described earlier (Hoffmann et al., 2016). For wild‐type, Δ*nifA1‐A2*, Δ*anfA*, or Δ*mopAB* cultures, 686 and 725, 746 and 758, 691 and 723, or 637 and 633 proteins, respectively, were identified in at least two of three replicates under both −Mo and +Mo conditions (Table A2 in Appendix 1). The vast majority of known *R*.* capsulatus* nitrogen fixation proteins encoded by the four nitrogen fixation gene clusters, A–D (Figure 1a), was differentially produced validating the reliability of our datasets (Table 1; Masepohl & Klipp, 1996; Schüddekopf et al., 1993; Strnad et al., 2010). In the Δ*nifA1‐A2* strain, 30 and 16 proteins were either missing or significantly downregulated as compared to the wild type upon −Mo and +Mo conditions, respectively. In the Δ*anfA* strain, 26 and 18 proteins were either missing or significantly downregulated as compared to the wild type upon −Mo and +Mo conditions, respectively. These findings are consistent with the function of NifA and AnfA as transcriptional activators. In the Δ*mopAB* strain, 15 and 9 proteins were upregulated as compared to the wild type upon −Mo and +Mo conditions, respectively, compatible with the repressor function of MopA and MopB. The abundance of proteins identified is listed in the Table S1. The complete data set is available via ProteomeXchange with identifier PXD013515.

### 3.2 NifA is required for the production of both nitrogenases

Table 1 shows the levels of nitrogen fixation proteins identified by proteome profiling of *R*.* capsulatus* wild‐type and mutant strains devoid of NifA or AnfA or MopAB in response to Mo availability. Mo stimulated the accumulation of the Mo‐nitrogenase proteins, NifHDK, while the levels of Fe‐nitrogenase proteins, AnfHDGK, were largely reduced by Mo, findings well in line with earlier studies on the *R*.* capsulatus* molybdoproteome (Hoffmann et al., 2016). The levels of NifHDK, FdxD, and most products of nitrogen fixation cluster A (Figure 1a) were largely diminished or absent in the Δ*nifA1‐A2* strain. NifA‐dependent production of these proteins corresponds with their functions in protection of Mo‐nitrogenase from oxygen damage (FdxD), NifDK maturation (NifW), formation of FeS clusters (NifU1‐NifS), FeMoco biosynthesis (NifENX, NifT), and electron supply to nitrogenase (RnfB, RnfG) (Curatti & Rubio, 2014; Hoffmann et al., 2014; Jimenez‐Vicente et al., 2018; Schüddekopf et al., 1993).

Likewise, the levels of the Fe‐nitrogenase proteins AnfHDGK and the accessory proteins AnfO and Anf3 were strongly reduced or absent in the Δ*anfA* strain consistent with the function of AnfA as the activator of *anfHDGKOR* transcription (Table 1). Surprisingly, Fe‐nitrogenase production was also abolished in the Δ*nifA1‐A2* strain, suggesting that AnfA is essential, but not sufficient for *anfHDGKOR* expression. Consistent with the proteomic results, the Δ*nifA1‐A2* strain did not grow under N_2_‐fixing conditions, indicating that NifA is required for the synthesis of both Mo‐nitrogenase and Fe‐nitrogenase (Figure A1 in Appendix 2).

### 3.3 NifA is required for the production of the MorABC transporter

**Figure 2:**
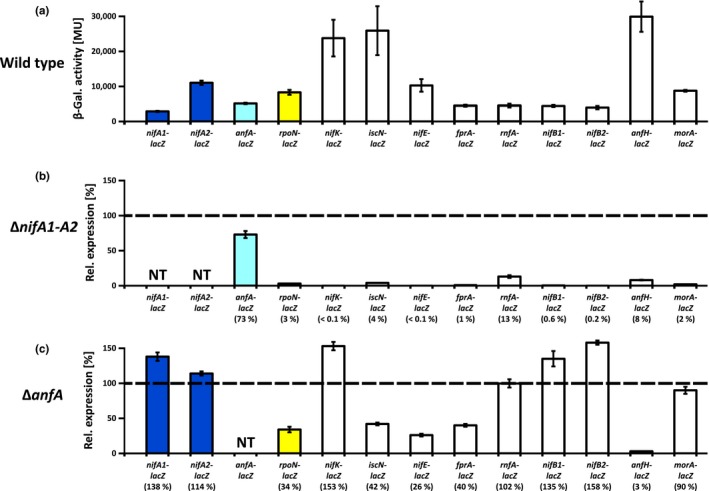
Expression of nitrogen fixation genes in *nifA* and *anfA* mutants. *Rhodobacter capsulatus* strains were phototrophically grown in RCV minimal medium with 10 mM serine but without Mo addition to allow simultaneous synthesis of NifA and AnfA and, consequently, production of Mo‐ and Fe‐nitrogenase. The strains used were as follows: the wild‐type strain B10S (a), the Δ*nifA1‐A2* strain YP202‐YP203 (b), and the Δ*anfA* strain KS94A (c) carrying chromosomally integrated plasmids with transcriptional *lacZ* fusions to *nifA1* and *nifA2* (pYP352), *anfA* (pLD37), *rpoN* (pLD28), *nifK* (pYP348), *iscN* (pEW58), *nifE* (pLD16), *fprA* (pLD52), *rnfA* (pLD15), *nifB1* and *nifB2* (pLD14), *anfH* (pMH187), and *morA* (pLD107). Plasmids pLD14 and pYP352 can each integrate at two chromosomal sites, because the duplicated *nifA1*‐*nifB1* and *nifA2*‐*nifB2* regions are identical except for the *nifA* promoters (Masepohl et al., 1988). LacZ (β‐galactosidase) activity is given in Miller units (a) (Miller, 1972) or shown as relative expression (b,c) with the wild‐type levels set as 100%. The results represent the means and standard deviations of at least five independent measurements. Colors of *nifA1*, *nifA2*, *anfA*, and *rpoN* are the same as in Figure 1

In this study, we identified a so far unrecognized member of the NifA regulon, MorA, previously found to belong to the Mo regulon (Table 1; Hoffmann et al., 2016; Wiethaus et al., 2006). The *morA* gene forms part of the *morAB* operon preceded by the divergently transcribed *morC* gene, whose products exhibit clear similarity to the high‐affinity molybdate transporter ModABC (Wiethaus et al., 2006). The levels of MorA and ModA were much higher under −Mo than under +Mo conditions (Table 1) consistent with earlier studies showing that Mo prevents the accumulation of these proteins (Hoffmann et al., 2016) and represses transcription of the *morAB* and *mopA*‐*modABC* operons (Wiethaus et al., 2006). Mo repression is mediated by MopA and MopB, which independently bind the *morA* and *mopA* promoters (Wiethaus et al., 2006). In contrast to the wild type, the Δ*mopAB* strain produced MorA even under +Mo conditions consistent with the absence of both Mo‐responsive repressors (Table 1). Consistent with the proteome data and as described below, NifA was strictly required for *morA*‐*lacZ* transcription (Figure 2) verifying that the *morAB* genes belong to the NifA regulon. In contrast, the *mopA*‐*modABC* operon belongs to the NtrC regulon (Bowman & Kranz, 1998; Kutsche et al., 1996). It is tempting to speculate that MorABC functions in molybdate uptake in addition to ModABC (Wang et al., 1993). However, deletion of the *morABC* genes in different genetic backgrounds did not affect in vivo Mo‐nitrogenase activity (Wiethaus et al., 2006), suggesting that MorABC contribution to Mo uptake was negligible at least under the tested conditions.

### 3.4 Validation of proteome profiling by reporter fusions

To validate the proteome profiling data, we generated transcriptional fusions between selected nitrogen fixation genes and the promoterless *lacZ* gene as described earlier (Hoffmann et al., 2016). The *lacZ* reporter gene was fused to cluster A genes *nifB1* (rcc03266), *nifA1* (rcc03267), *iscN* (rcc03272), *nifE* (rcc03280), *fprA* (rcc03286), and *rnfA* (rcc03287); cluster B genes *nifB2* (rcc00566), *nifA2* (rcc00567), *rpoN* (rcc00568), and *nifK* (rcc00570); cluster D genes *anfA* (rcc00584), and *anfH* (rcc00585); and to the *morA* (rcc02219) gene (for genetic organization of nitrogen fixation clusters A–D and the *mor* region, see Figure 1a). These reporter fusions were chromosomally integrated into the *R*.* capsulatus* wild‐type, Δ*nifA1‐A2*, and Δ*anfA* strains. Following growth of the reporter strains in −Mo medium supplemented with serine as a fixed nitrogen source, LacZ (β‐galactosidase) activities were determined (Figure 2).

In the wild‐type background, all tested reporter fusions were clearly expressed albeit to different levels (Figure 2a). The most strongly expressed genes were *nifK*, *anfH*, and *iscN*. Strong expression of *nifK* and *anfH* was consistent with high NifHDK and AnfHDGK levels (Table 1). Despite strong *iscN* expression, however, the IscN protein level was relatively low. This discrepancy is possibly explained by posttranscriptional control of *iscN* expression (Hoffmann et al., 2016). Expression of the *nifK*, *iscN*, *nifE*, *fprA*, *rnfA*, *nifB1*, *nifB2*, and *morA* genes was strongly reduced in the Δ*nifA1‐A2* strain (Figure 2b), consistent with the proteome studies (Table 1). These genes form part of the *nifHDK*, *iscN*‐*nifU1*‐*nifSVW*, *nifENX*‐rcc03277‐rcc03276‐*fdxB*‐*nifQ*‐rcc03273, *fprA*‐*fdxCN*‐rcc03282‐*rnfF*‐rcc03281, *rnfABCDGEH*, *nifB1*‐rcc03265‐*nifZT*‐rcc03262, *nifB2*, and *morAB* operons, all of which are preceded by putative NifA and RpoN binding sites (Figure 1b) (Masepohl, Angermüller, et al., 1993; Moreno‐Vivian, Hennecke, Pühler, & Klipp, 1989; Moreno‐Vivian, Schmehl, Masepohl, Arnold, & Klipp, 1989; Pollock, Bauer, & Scolnik, 1988; Schmehl et al.., 1993). Hence, it seems that NifA directly activates transcription of these operons.

Consistent with the absence of the AnfHDGKOR proteins in the Δ*anfA* and Δ*nifA1‐A2* strains (Table 1), *anfH* expression was 30‐fold and 12‐fold reduced, respectively, in these backgrounds (Figure 2). In the Δ*anfA* strain, expression of the *nifK*, *nifB1*, *nifB2*, *nifA1*, and *nifA2* genes was about 1.5‐fold higher than in the wild type, while expression of the *iscN*, *nifE*, *fprA*, and *rpoN* genes was threefold to fourfold lower than in the wild type. Apparently, AnfA functions not only as an activator, but may also act as a repressor. Alternatively, AnfA may activate a yet unknown repressor gene. In summary, these findings indicate that AnfA is essential for activation of *anfH* expression, while it exhibits comparatively low impact on the expression of other nitrogen fixation genes.

### 3.5 NifA controls AnfA‐mediated gene activation by controlling *rpoN* expression

The *nifA2* gene was about fourfold stronger expressed than the *nifA1* gene, when cultures were grown in the presence of serine (Figure 2a), and diazotrophic growth of the Δ*nifA2* strain was delayed as compared to the wild‐type and Δ*nifA1* strains (Figure A1 in Appendix 2). These observations are in line with a previous report demonstrating that NtrC‐dependent *nifA2* expression is further enhanced by RegA (Elsen et al., 2000). As is the case for *nifA1* and *nifA2*, activation of *anfA* expression depends on NtrC (for a regulatory model, see Figure 5) (Kutsche et al., 1996). While NtrC activates its target genes in concert with the housekeeping sigma factor RpoD, target gene activation by NifA and AnfA depends on the alternative sigma factor RpoN (Bowman & Kranz, 1998).

Transcription of *rpoN* was almost completely abolished in the Δ*nifA1‐A2* strain (Figure 2b), showing that NifA is the master regulator of *rpoN* expression. The fact that *rpoN* transcription was more than twofold reduced in the Δ*anfA* strain (Figure 2c) suggests that AnfA is required for maximal *rpoN* expression possibly by acting as a coactivator as described above for RegA.

Despite almost full expression of the *anfA* gene in the Δ*nifA1‐A2* strain, AnfA‐mediated *anfH* expression was very low (Figure 2b). Since *rpoN* expression was also even lower in the Δ*nifA1‐A2* strain (Figure 2b), we suspected that low *anfH* expression resulted from shortage of RpoN.

**Figure 3:**
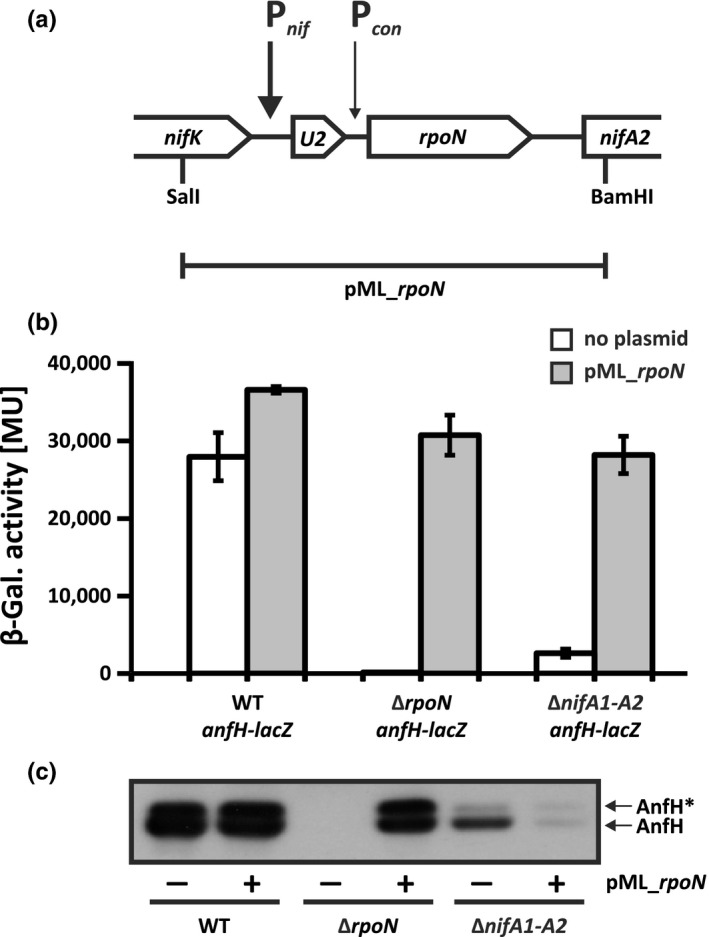
Analysis of *anfH* expression in *rpoN* and *nifA* mutants. (a) Organization of the *Rhodobacter capsulatus rpoN* region. The *nifU2*‐*rpoN* superoperon encompasses a NifA‐activated promoter (P*_nif_*) and a constitutive promoter (P*_con_*). The indicated 3 kbp SalI‐BamHI fragment was cloned into the broad‐host‐range vector pML5 (Labes et al., 1990) resulting in plasmid pML_*rpoN*. (b) Transcription of *anfH*‐*lacZ* in Δ*rpoN* and Δ*nifA1‐A2* backgrounds. *R*.* capsulatus* strains were phototrophically grown in RCV minimal medium with 10 mM serine (no Mo added). The strains used were as follows: the wild‐type strain B10S, the Δ*rpoN* strain YP201, and the Δ*nifA1‐A2* strain YP202‐YP203 carrying a chromosomal *anfH*‐*lacZ* fusion (pMH187) and plasmid pML_*rpoN* as indicated. LacZ (β‐galactosidase) activity is given in Miller units (Miller, 1972). The results represent the means and standard deviations of at least five independent measurements. (c) Accumulation of AnfH in Δ*rpoN* and Δ*nifA1‐A2* backgrounds. *R*.* capsulatus* strains were grown as in (b). Equal amounts of protein were loaded in each lane as determined by total protein staining (data not shown). Western analyses were done in triplicate with one representative result shown in (c). The strains used were as follows: B10S, YP201, and YP202‐YP203 carrying plasmid pML_*rpoN* as indicated. The ADP‐ribosylated form of AnfH is marked by an asterisk

The *rpoN* gene belongs to the *nifU2*‐*rpoN* superoperon, which has a weak constitutive primary promoter upstream of the *rpoN* coding region and a NifA‐RpoN‐activated secondary promoter upstream of the *nifU2* coding region (Figure 3a) (Cullen, Foster‐Hartnett, Gabbert, & Kranz, 1994). The primary promoter is essential for basal *rpoN* expression, while the secondary promoter is required to enhance *nifU2*‐*rpoN* expression under nitrogen‐fixing conditions.

We speculated that multiple copies of the *nifU2*‐*rpoN* operon might enhance *rpoN* expression and, consequently, enhance AnfA‐RpoN‐mediated *anfH* expression in the Δ*nifA1‐A2* strain. To test this assumption, the *nifU2*‐*rpoN* operon was cloned into the broad‐host‐range vector pML5 (Labes, Pühler, & Simon, 1990). The resulting hybrid plasmid pML_*rpoN* restored *anfH* expression in a Δ*rpoN* strain, thus proving *in‐trans* complementation by plasmid‐borne *rpoN* (Figure 3b). Noteworthy, this strain is capable of synthesizing NifA, the main mediator of *rpoN* expression. Plasmid pML_*rpoN* also restored *anfH* expression in the Δ*nifA1‐A2* background, indicating that this plasmid mediates sufficient production of RpoN to re‐establish AnfA‐mediated *anfH* transcription even in the absence of NifA and suggesting that RpoN is the only NifA‐dependent factor needed for gene activation by AnfA (Figure 3b).

Since plasmid pML_*rpoN* restored *anfH* transcription in the Δ*nifA1‐A2* strain, we next asked for the AnfH protein level in this background. To answer this question, we examined accumulation of the AnfH protein by Western analysis (Figure 3c). As expected from an earlier study (Masepohl, Krey, et al., 1993), two bands corresponding to the modified (ADP‐ribosylated) and unmodified AnfH protein were observed in the wild‐type irrespective of the presence or absence of pML_*rpoN*. In the Δ*rpoN* background, significant levels of AnfH were only detected in the presence but not in the absence of pML_*rpoN*, findings well in line with the *anfH* transcription pattern (Figure 3b). Despite clear *anfH* transcription in the Δ*nifA1‐A2* strain carrying pML_*rpoN*, however, only very low AnfH levels were found in this strain. Unexpectedly, somewhat higher AnfH levels were detected in the Δ*nifA1‐A2* strain lacking pML_*rpoN*. Together, these findings suggest that NifA indirectly controls Fe‐nitrogenase production at the transcriptional level (via RpoN) and at the posttranscriptional level (by a yet unknown mechanism).

### 3.6 NifA is required for activity of Fe‐nitrogenase

Since the Δ*nifA1‐A2* strain carrying pML_*rpoN* produced low levels of AnfH (Figure 3c), we wondered whether this strain exhibited some Fe‐nitrogenase activity. To answer this question, we examined diazotrophic growth of selected *R*.* capsulatus* strains under −Mo conditions as described earlier (Hoffmann et al., 2016).

**Figure 4:**
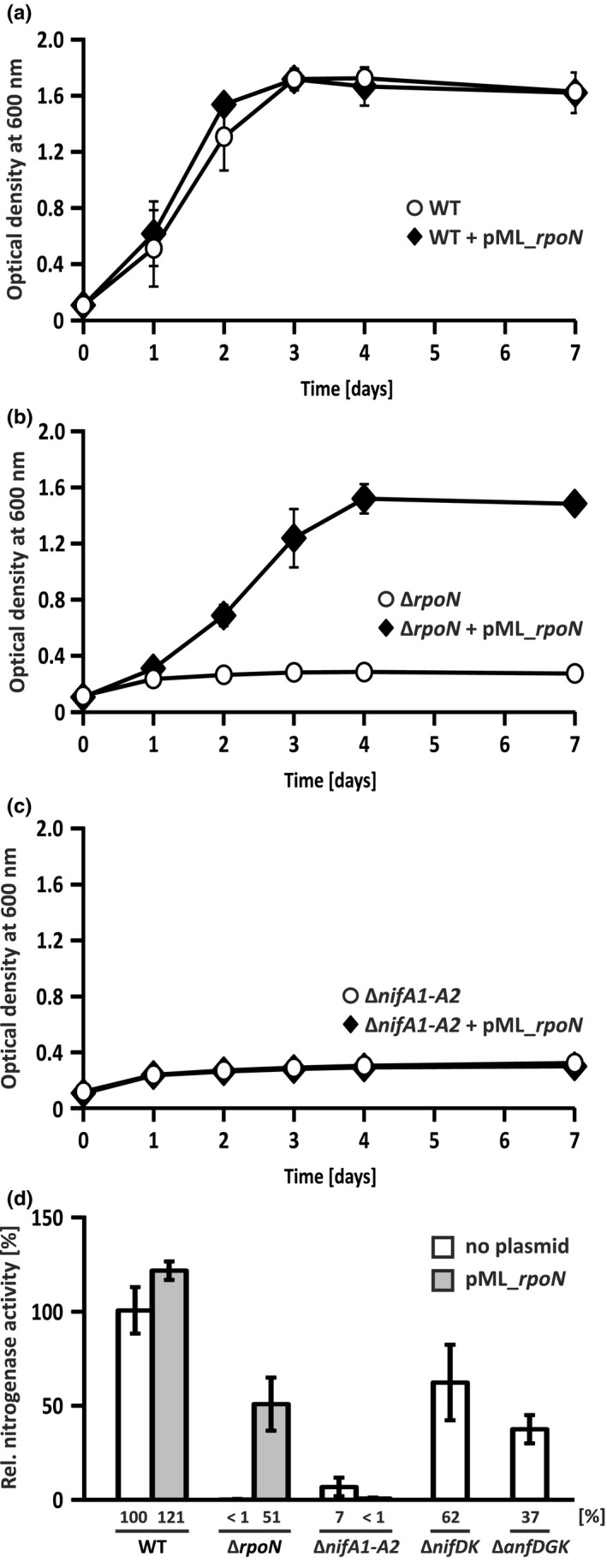
Analysis of diazotrophic growth and nitrogenase activity in *rpoN* and *nifA* mutants. (a‐c) Diazotrophic growth. *Rhodobacter capsulatus* strains were phototrophically grown in RCV minimal medium without Mo addition under a pure N_2_ atmosphere (no fixed nitrogen source added). The strains used were as follows: the wild‐type strain B10S (a), the Δ*rpoN* strain YP201 (b), and the Δ*nifA1‐A2* strain YP202‐YP203 (c) carrying plasmid pML_*rpoN* as indicated. The results represent the means and standard deviations of two independent measurements. (d) Nitrogenase activity. *R*.* capsulatus* strains were phototrophically grown in RCV medium with serine (no Mo added) prior to the determination of nitrogenase activity by the acetylene reduction assay. The 100% value corresponds to 521 nmol ethylene produced hr^−1^ mg protein^−1^. The results represent the means and standard deviations of three independent measurements

Wild‐type cultures with and without pML_*rpoN* grew comparably well, indicating that multiple *rpoN* copies did not negatively affect nitrogen fixation or general fitness (Figure 4a). Consistent with the requirement of RpoN for target gene activation by NifA and AnfA, the Δ*rpoN* strain did not grow diazotrophically (Figure 4b; Fischer, 1994; Merrick, 1993; Schüddekopf et al., 1993). As expected, diazotrophic growth of the Δ*rpoN* strain was re‐established by the pML_*rpoN* plasmid albeit growth was delayed as compared to the wild type (Figure 4b). In contrast, diazotrophic growth of the Δ*nifA1‐A2* strain was not recovered by this plasmid (Figure 4c).

In addition, in vivo nitrogenase activity was determined by the acetylene reduction assay (Figure 4d). Nitrogenase activities of wild‐type and mutant strains were consistent with their ability to grow diazotrophically. Under −Mo conditions, both Mo‐nitrogenase and Fe‐nitrogenase contributed to the total nitrogenase activity (Figure 4d). Together, these findings suggest that the level of AnfH (and possibly AnfDGK) observed in the Δ*nifA1‐A2* strain carrying pML_*rpoN* was not sufficient for diazotrophic growth. Alternatively, at least one NifA‐dependent factor (other than RpoN) required for activity of Fe‐nitrogenase might also be limiting in this background. Likely, candidates are the Fe‐nitrogenase cofactor biosynthesis proteins NifB and NifV, and the Rnf proteins required for electron supply (Table 1; Schüddekopf et al., 1993).

## 4 DISCUSSION

In *R*.* capsulatus*, production of Mo‐nitrogenase and Fe‐nitrogenase is induced or repressed by Mo, respectively (Figure A1 in Appendix 2; Demtröder et al., 2019; Masepohl, 2017; Masepohl & Klipp, 1996). Intriguingly, both nitrogenases are synthesized at the same time at low nanomolar Mo concentrations corresponding to the Mo levels in freshwater habitats of *R*.* capsulatus* (Glass, Axler, Chandra, & Goldman, 2012; Hoffmann et al., 2016; Weaver, Wall, & Gest, 1975). Hence, simultaneous synthesis of Mo‐ and Fe‐nitrogenases probably reflects the natural situation rather than being the exception. In this study, we unraveled the regulation of the two complementary nitrogenases in *R*.* capsulatus*.

Synthesis of the Mo‐ and Fe‐nitrogenases requires the transcriptional activators NifA and AnfA, respectively, as shown by this and earlier studies (Kutsche et al., 1996; Schüddekopf et al., 1993). This study now revealed that synthesis of Fe‐nitrogenase also depends on NifA (Table 1), suggesting that AnfA is essential, but not sufficient for synthesis of the Mo‐free isoenzyme.

**Figure 5:**
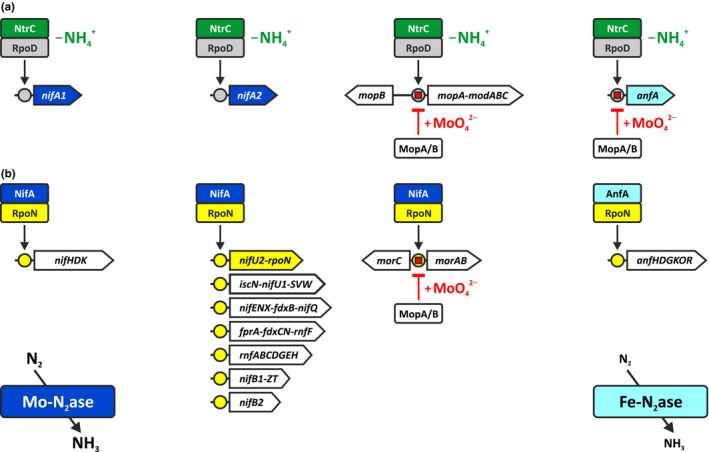
Model of the nitrogen fixation regulon in *Rhodobacter capsulatus*. (a) The NtrC regulon. In the absence of ammonium (−NH_4_^+^), NtrC activates transcription of the *nifA1*, *nifA2*, *mopA*‐*modABC*, and *anfA* genes in concert with the housekeeping sigma factor RpoD (Foster‐Hartnett et al., 1994; Kutsche et al., 1996). (b) The NifA and AnfA regulons. For clarity, NifA1 and NifA2 are collectively shown as NifA. Either MopA or MopB is sufficient to repress transcription of the *mopA*‐*modABC*, *anfA*, and *morAB* genes by binding to Mo‐boxes (red squares) in the presence of molybdate (+MoO_4_^2−^) (Wiethaus et al., 2006). NifA and AnfA activate transcription of their target genes in concert with the alternative sigma factor RpoN (this study; Cullen et al., 1994; Schüddekopf et al., 1993). For further details, see text. Colors of *ntrC*, *nifA1*, *nifA2*, *anfA*, and *rpoN* are the same as in Figure 1

The main reason for the NifA dependency of the Fe‐nitrogenase is that NifA is crucial for expression of *rpoN* (Figure 2b) coding for the sigma factor indispensable for target gene activation by both NifA and AnfA. Of note, NifA and AnfA are the only transcriptional activators requiring RpoN, while NtrC cooperates with the housekeeping sigma factor RpoD (Bowman & Kranz, 1998). In a hierarchical fashion, NifA controls expression of *rpoN* and as a consequence, AnfA‐dependent expression of the Fe‐nitrogenase genes. Apparently, RpoN is the only NifA‐dependent factor required for target gene activation by AnfA since plasmid‐borne *rpoN* restored *anfH* transcription in the Δ*nifA1‐A2* strain (Figure 3b). However, plasmid‐borne *rpoN* failed to restore Fe‐nitrogenase activity in this background (Figure 4c,d). Possibly, a NifA‐dependent factor other than RpoN acting at the posttranscriptional level is required for Fe‐nitrogenase production. In addition, NifA is required for expression of genes involved in FeFeco biosynthesis and electron supply to Fe‐nitrogenase including the *nifB* and *rnf* genes, respectively (Figure 5; Table 1; Schmehl et al., 1993; Schüddekopf et al., 1993).

Besides *R*.* capsulatus*, control of Mo‐free nitrogenases by NifA‐like regulators has been examined in *A*.* vinelandii*, which is one of few species capable of synthesizing all three nitrogenases (Loveless & Bishop, 1999; Mus et al., 2018; Setubal et al., 2009). In *A*.* vinelandii*, synthesis of Mo‐, V‐, and Fe‐nitrogenases depends on NifA, VnfA, and AnfA, respectively (Drummond et al., 1996; Walmsley, Toukdarian, & Kennedy, 1994). While NifA is dispensable for VnfA‐mediated activation of the V‐nitrogenase genes, it is required for AnfA‐mediated activation of the Fe‐nitrogenase genes as is the case in *R*.* capsulatus*. In contrast to *R*.* capsulatus*, however, the *rpoN* gene does not belong to the NifA regulon in *A*.* vinelandii* (Merrick, Gibbins, & Toukdarian, 1987), suggesting that the underlying control mechanisms involve different NifA‐activated factors, namely RpoN in *R*. *capsulatus* and an unknown factor (other than RpoN) in *A*.* vinelandii*.

Sigma factors of the RpoN family are widespread in both diazotrophic and nondiazotrophic bacteria, most of which have a single copy of the *rpoN* gene, but some have two or more copies (Domenzain, Camarena, Osorio, Dreyfus, & Poggio, 2012; Mittenhuber, 2002; Studholme & Buck, 2000). While *R*.* capsulatus* has only one *rpoN* gene, its close relative, *Rhodobacter sphaeroides*, has four *rpoN* copies (Poggio, Osorio, Dreyfus, & Camarena, 2002). In the *Rhodobacteraceae* family, *rpoN* is often linked to nitrogen fixation genes, namely *nifU2* (*R*.* capsulatus*), *fixABCX* (*R*.* blasticus*, *Rhodovulum sulfidophilum*), or *nifUSVW* (*R*.* sphaeroides*, *R*.* azotoformans*) (Domenzain et al., 2012; Meijer & Tabita, 1992; Poggio et al., 2002). Likewise, in many members of the *Rhizobiaceae* family (including *Rhizobium etli*, *Rhizobium mesoamericanum*, and *Rhizobium tropici*), *rpoN* is linked to the nitrogen fixation gene *prxS*, which codes for a peroxiredoxin involved in bacteroid protection against oxidative stress (Dombrecht et al., 2005). As expected from the genetic organization, activation of *rpoN* genes by NifA has been demonstrated in *R*.* capsulatus*, *R*.* sphaeroides*, and *R*.* etli* and is likely to be the case in the other strains (This study; Dombrecht et al., 2005; Meijer & Tabita, 1992). These findings indicate that integration of the *rpoN* gene into the NifA regulon is a common theme in diazotrophic alphaproteobacteria. Apparently, NifA control of *rpoN* in different lineages evolved by several independent gene rearrangements as indicated by integration of *rpoN* into different nitrogen fixation operons.

## 5 CONCLUSIONS

In *R*.* capsulatus*, NifA controls the Fe‐nitrogenase system in at least two ways. (a) NifA controls AnfA‐mediated *anfHDGK* transcription via RpoN. (b) NifA controls Fe‐nitrogenase activity via its requirement for FeFeco biosynthesis and electron supply (Figure 5). Hence, the Fe‐nitrogenase system is largely integrated into the Mo‐nitrogenase system rather than acting as an independent, alternative system. The main function of AnfA is the activation of the Fe‐nitrogenase operon in response to Mo availability, while its effects on other nitrogen fixation genes are less pronounced.

To our knowledge, NifA control of Fe‐nitrogenase has been examined in only two species, the alphaproteobacterium *R*.* capsulatus* (this study) and the gammaproteobacterium *A*.* vinelandii* (Walmsley et al., 1994). However, NifA control of Fe‐nitrogenase in these species involves different factors, namely RpoN in *R*.* capsulatus* and a yet unknown factor (other than RpoN) in *A*.* vinelandii*. Since these diazotrophs are only distantly related, it is tempting to speculate that NifA control of Fe‐nitrogenase is a general feature in proteobacteria.

## CONFLICT OF INTERESTS

None declared.

## AUTHOR CONTRIBUTIONS

LD and BM involved in the conceptualization; BM involved in the funding acquisition; LD, YP, and SS investigated the study; and LD, JEB, BM wrote the manuscript.

## ETHICS STATEMENT

None required.

## Data Availability

The mass spectrometry proteomics data have been deposited to the ProteomeXchange Consortium via the PRIDE (Perez‐Riverol et al., 2019) partner repository with the data set identifier PXD013515.

## APPENDIX 1

**Table A1:** *Rhodobacter capsulatus* strains and plasmids

Strain or plasmid	Relevant characteristic^a^	Source or reference
Strains		
B10S	*R*.* capsulatus* wild type; Sm^r^	Klipp et al. (1988)
BS85	Polar *nifD*::Sp mutant (Δ*nifDK*) of B10S; Sm^r^, Sp^r^	Hoffmann et al. (2014)
KS94A	*anfA*::Sp mutant (Δ*anfA*) of B10S; Sm^r^, Sp^r^	Wang et al. (1993)
PBK2	*ntrC*::Km mutant (Δ*ntrC*) of B10S; Sm^r^, Km^r^	Kutsche et al. (1996)
R423CI	(*mopA‐mopB*)::Gm mutant (Δ*mopAB*) of B10S; Sm^r^, Gm^r^	Wang et al. (1993)
YP201	*rpoN*::Gm mutant (Δ*rpoN*) of B10S; Sm^r^, Gm^r^	Hoffmann et al. (2014)
YP202	*nifA2*::Km mutant (Δ*nifA2*) of B10S; Sm^r^, Km^r^	Hoffmann et al. (2014)
YP203	*nifA1*::Gm mutant (Δ*nifA1*) of B10S; Sm^r^, Gm^r^	Hoffmann et al. (2014)
YP202‐YP203	*nifA1*::Gm, *nifA2*::Km mutant (Δ*nifA1‐A2*) of B10S; Sm^r^, Km^r^, Gm^r^	Hoffmann et al. (2014)
YP243	Polar *anfD*::Sp mutant (Δ*anfDGK*) of B10S; Sm^r^, Sp^r^	This study
Plasmids		
pEW58	pYP168 derivative carrying *iscN*‐*lacZ*; Tc^r^ oriT	Hoffmann et al. (2016)
pLD14	pYP168 derivative carrying *nifB*‐*lacZ*; Tc^r^ oriT	This study
pLD15	pYP168 derivative carrying *rnfA*‐*lacZ*; Tc^r^ oriT	This study
pLD16	pYP168 derivative carrying *nifE*‐*lacZ*; Tc^r^ oriT	This study
pLD28	pYP168 derivative carrying *rpoN*‐*lacZ*; Tc^r^ oriT	This study
pLD37	pYP168 derivative carrying *anfA*‐*lacZ*; Tc^r^ oriT	This study
pLD52	pYP168 derivative carrying *fprA*‐*lacZ*; Tc^r^ oriT	This study
pLD107	pYP168 derivative carrying *morA*‐*lacZ*; Tc^r^ oriT	This study
pMH187	pYP168 derivative carrying *anfH*‐*lacZ*; Tc^r^ oriT	This study
pML5	Mobilizable broad‐host‐range vector; Tc^r^	Labes et al. (1990)
pML_*rpoN*	pML5 derivative carrying *R*.* capsulatus nifU2*‐*rpoN*	This study
pYP5	pBSL15 derivative carrying *lac*TeT	Gisin et al. (2010)
pYP168	pUC18 derivative with reduced multiple cloning site	Hoffmann et al. (2016)
pYP348	pYP168 derivative carrying *nifK*‐*lacZ*; Tc^r^ oriT	Hoffmann et al. (2016)
pYP352	pYP168 derivative carrying *nifA*‐*lacZ*; Tc^r^ oriT	This study

Gm, gentamicin; Km, kanamycin; Sp, spectinomycin; Sm, streptomycin; Tc, tetracycline.

**Table A2:** Global responses of the *Rhodobacter capsulatus* wild‐type and mutant proteomes

	Number of identified proteins^a^			
	Wild type	Δ*nifA1*‐*A2*	Δ*anfA*	Δ*mopAB*
−Mo	686	746 (30 ↓)	691 (26 ↓)	637 (15 ↑)
+Mo	725	758 (16 ↓)	723 (18 ↓)	633 (9 ↑)

^a^ Numbers in brackets represent proteins up‐ (↑) or downregulated (↓) in comparison with the wild type.
