# Supplementary material for: NifA is the master regulator of both nitrogenase systems in Rhodobacter capsulatus
Source: Microbiologyopen. 2019 Aug 22;8(12):e921. doi: 10.1002/mbo3.921 (PMC6925177; doi:10.1002/mbo3.921)
Supplement: Supplementary file 1 [file MBO3-8-e921-s001.pdf]

Abundances of proteins identified by mass spectrometry in Rhodobacter capsulatus wild type (WT) or nifA1-A2, anfA or mopAB mutants upon growth without (-) or with (+) addition of 10 µM Na2MoO4 (Mo). Normalized protein concentrations are given as fmol/column for arithmetic means and standard deviations were calculated (%SD). When no standard deviation is given, the protein was found in only one of the biologial replicates.

protein ID	gene	protein function	WT-Mo		WT +Mo		ΔnifA1-A2 -Mo		ΔnifA1-A2 +Mo		ΔanfA-Mo		ΔanfA +Mo		ΔmopAB-Mo		ΔmopAB +Mo	
			mean	%SD	mean	%SD	mean	%SD	mean	%SD	mean	%SD	mean	%SD	mean	%SD	mean	%SD
YP_003576174	dnaN	DNA polymerase III subunit beta	16,02	15,55	11,82	9,65	16,91	13,06	17,68	3,09	12,67	13,59	13,99	5,13	11,08	22,80	13,87	28,55
YP_003576176	gyrB	DNA gyrase subunit B	2,19	6,62	3,34	20,93	3,30	41,53	4,43	16,15	2,77	17,36	3,88	6,57	3,63	10,03	4,22	12,17
YP_003576177	ntaB	nitrilotriacetate monooxygenase sub			3,24		2,08		2,53				2,86					
YP_003576186	xylA	xylose isomerase	2,74		4,24	2,65	3,35	16,91	3,86	24,72			6,04	1,49			4,63	14,46
YP_003576187	xylB	xylulokinase					0,48											
YP_003576188	xylG	xylose ABC transporter ATP-binding p			0,80		0,83											
YP_003576190	xylF	xylose ABC transporter xylose-bindin	85,24	9,94	212,43	34,37	44,26	22,59	83,60	16,80	108,61	13,32	171,62	13,43	41,68	5,25	71,64	16,22
YP_003576193	rcc00021	cytochrome c peroxidase			0,65				1,18									
YP_003576194	rcc00022	aldo/keto reductase family oxidored	3,59	11,85	4,42	0,76	5,76	9,45	6,21	16,25	3,79	21,61	6,06	8,54	4,07	17,09	5,35	16,38
YP_003576196	gcdH	glutaryl-CoA dehydrogenase	1,02															
YP_003576198	rcc00026	hypothetical protein	2,08	38,70			0,97											
YP_003576199	rcc00027	surface antigen											1,54					
YP_003576201	secB	protein-export chaperone SecB	12,32	12,97	9,47	18,83	13,61	15,20	12,82	12,43	9,30	10,96	10,39	3,09	7,98	12,79	10,96	24,86
YP_003576203	rcc00031	import inner membrane translocase	3,19	50,12	1,26	0,08	0,94										1,29	51,13
YP_003576205	rcc00033	hypothetical protein															0,51	
YP_003576206	hslU	ATP-dependent hsl protease ATP-bin	2,33		3,14	16,54	2,98	30,38	3,45	17,14	25,68	91,14	2,27	2,22	2,43		3,20	27,33
YP_003576208	trxA1	thioredoxin	82,67	13,10	97,45	27,54	94,65	30,94	74,54	11,15	102,22	17,29	90,46	6,02	73,18	20,55	72,56	34,14
YP_003576212	rcc00040	aminoglycoside phosphotransferase											0,42					
YP_003576215	regB	sensor histidine kinase RegB			2,07		2,30		2,05				1,72				1,05	
YP_003576216	senC	electron transport protein SenC			3,82		4,44	26,75			2,69		1,63		2,85		2,30	
YP_003576217	regA1	photosynthetic apparatus regulatory	29,00	8,43	30,86	7,65	31,77	8,99	32,87	4,45	33,91	11,04	33,96	7,88	25,84	0,63	28,67	1,86
YP_003576218	hvrA	trans-acting regulatory protein HvrA	72,14	14,97	74,85	14,79	88,62	43,43	31,68	21,43	74,19	41,95	37,61	4,76	31,09	19,79	31,08	26,84
YP_003576221	ahcY	adenosylhomocysteinase	41,77	6,29	36,69	17,96	46,48	9,18	56,91	6,94	38,53	13,45	44,68	5,43	37,09	11,33	43,89	7,63
YP_003576222	rcc00050	hypothetical protein			9,07	4,23	8,81	10,15	10,95	5,91	9,35	6,43	9,19	7,81	4,79	4,72	6,66	6,01
YP_003576223	rcc00051	enoyl-CoA hydratase/isomerase					2,20	28,37	1,36		1,90		1,46				1,87	38,68
YP_003576224	rcc00052	hypothetical protein	9,64	13,36	6,88	4,46	7,87	11,61	6,97	6,26	5,82	7,55	7,81	16,69	4,52	1,21	5,20	6,87
YP_003576225	rcc00053	U62 family peptidase	3,27	23,94	3,03	24,05	4,12	16,86	2,55	17,50	2,29		3,07	22,36	3,20		3,53	23,06
YP_003576232	parB	chromosome-partitioning protein Pa	3,16	3,55	2,73	22,66	3,35	8,31	3,53	9,39	2,63	16,82	3,11	5,17	2,86	10,43	3,24	6,77
YP_003576233	parA	chromosome-partitioning protein Pa	3,92	13,88	3,40	6,81	3,96	15,09	4,37	4,88	3,13	20,12	3,34	2,19			2,72	
YP_003576235	gidA	tRNA uridine 5-carboxymethylamino							2,46	9,15			0,81				3,15	
YP_003576236	trmE	tRNA modification GTPase TrmE			1,57		1,82											
YP_003576237	rho	transcription termination factor Rho	7,81	23,05	6,05	22,77	7,39	24,35	8,18	8,90	5,32	15,01	7,83	3,46	6,91	18,36	8,99	25,84
YP_003576240	aroE	shikimate 5-dehydrogenase					1,02		0,98								1,62	

YP_003576243	nadA	quinolinate synthetase A	1,09		2,65	16,04	0,86		1,53	38,63	1,37		0,87		1,13	15,51	10,21	
YP_003576244	nadB	L-aspartate oxidase	0,81		0,76		4,13				0,84							
YP_003576245	nadC	nicotinate-nucleotide diphosphorylas	1,08		1,42	37,49	1,42	10,96	2,30	32,19	1,62	41,29	1,93	35,44	1,19	12,35	1,19	20,55
YP_003576246	nudH	(di)nucleoside polyphosphate hydrol	5,13				5,39	1,85	6,17		4,28	14,38	3,18		3,42			
YP_003576247	ctpA	C-terminal processing peptidase	12,39	14,75	15,38	8,50	14,23	14,98	12,98	7,11	14,85	17,43	19,03	2,81	11,90	1,61	15,94	6,84
YP_003576249	gpmI	2_3-bisphosphoglycerate-independe	6,93	5,88	5,29	23,18	7,54	19,55	6,49	17,66	5,99	25,37	6,62	6,84	5,52	21,25	7,72	26,21
YP_003576251	rcc00079	iojap-related protein	1,50															
YP_003576253	leuC	3-isopropylmalate dehydratase large	12,83	16,77	12,35	12,96	12,16	7,49	13,61	5,98	13,48	4,94	15,49	2,29	19,07	10,75	16,04	22,78
YP_003576254	leuD	3-isopropylmalate dehydratase small	21,56	11,32	16,81	3,38	17,27	11,42	15,01	3,88	19,02	5,62	16,86	11,95	23,63	3,36	18,22	7,39
YP_003576257	leuB	3-isopropylmalate dehydrogenase	20,06	18,20	14,36	15,83	18,20	17,30	19,85	7,13	15,01	11,00	17,80	4,83	15,37	15,95	21,19	27,26
YP_003576263	feoA2	ferrous iron transport protein A													2,86			
YP_003576265	rcc00093	hypothetical protein													1,02			
YP_003576272	rcc00100	ABC transporter ATP-binding protein					1,31						0,89					
YP_003576275	rcc00103	ABC transporter periplasmic substrat	7,21	5,96	8,38	24,15	12,44	14,54	14,92	4,56	8,11	6,13	14,42	6,54	4,84	13,24	10,82	26,86
YP_003576285	rnd1	ribonuclease D	2,96	3,77	3,35	6,93	2,91	16,49	3,65	21,84	4,04		3,60	4,07			3,46	2,40
YP_003576286	kdsD	arabinose 5-phosphate isomerase	1,84		1,76	13,01	2,08		2,32	17,09	1,60	23,58	2,08	8,96	1,94		2,04	14,51
YP_003576289	rcc00117	ABC transporter ATP-binding protein					2,18											
YP_003576290	rcc00118	sigma 54 modulation protein/ribosor	65,60	4,33	73,65	5,19	71,95	13,42	63,07	3,43	66,57	9,31	77,70	6,07	48,94	2,92	72,67	2,40
YP_003576291	ptsN	nitrogen regulatory protein	3,34		3,17		3,74	24,01	3,75	16,86	4,45		3,45	1,21	1,33		2,99	2,76
YP_003576292	rcc00120	hypothetical protein											0,92					
YP_003576297	galU	UTP--glucose-1-phosphate uridylyltra	13,49	15,02	12,18	20,82	14,15	11,06	17,22	4,34	12,19	18,58	15,03	9,29	11,79	7,65	16,44	8,41
YP_003576299	cysQ	3'(2')_5'-bisphosphate nucleotidase	10,65	8,47	11,04	9,26	15,57	6,79	15,04	2,77	11,33	6,30	13,70	3,72	7,85	9,41	11,01	9,06
YP_003576301	rcc00129	LacI family transcriptional regulator	2,56	52,98	2,81	19,71	3,77	3,94	3,00	25,41	2,76	12,66	2,85	22,06	2,43	7,13	2,57	22,32
YP_003576302	rcc00130	radical SAM family protein			2,21													
YP_003576303	rcc00131	tRNA/rRNA cytosine-C5-methylase							0,55									
YP_003576305	rcc00133	hypothetical protein	42,97	18,74	32,93	15,23	48,12	14,12	21,28	28,39	47,84	21,95	17,11	8,69	13,57	17,36	24,43	7,57
YP_003576315	etfA	electron transfer flavoprotein subuni	32,55	7,23	28,83	15,36	34,91	11,92	33,69	10,49	31,66	8,74	29,58	16,78	15,85	8,23	21,86	5,85
YP_003576316	etfB	electron transfer flavoprotein subuni	35,06	19,89	26,55	5,11	36,56	11,49	37,19	1,63	26,88	4,26	25,46	7,30	16,64	9,98	23,67	21,66
YP_003576318	parC	DNA topoisomerase IV subunit A	2,06		4,70	8,18	4,64	47,77	2,16	20,34	4,00	38,47	2,76	46,20	2,02	20,07	3,82	6,19
YP_003576319	tuf1	translation elongation factor Tu	591,92	6,00	565,51	9,69	705,55	15,38	876,00	3,56	626,79	6,87	699,97	10,41	603,19	4,04	688,11	12,72
YP_003576324	rcc00152	peptide ABC transporter periplasmic							1,41				2,04		2,30	5,83	3,36	10,37
YP_003576329	rcc00157	hypothetical protein	3,89	1,08	3,17		3,11		4,71	35,31	2,72		2,98		3,04		3,01	
YP_003576333	gltB	glutamate synthase (NADPH) subunit	5,71	7,67	5,07	24,31	6,78	20,62	6,92	4,22	5,12	17,69	5,82	3,65	8,48	19,20	11,69	48,73
YP_003576335	gltD	glutamate synthase (NADPH) subunit	6,40	12,23	5,07	14,90	7,36	30,96	6,39	6,66	5,19	23,78	5,85	1,77	8,38	19,36	9,34	21,25
YP_003576337	rcc00165	NAD-dependent epimerase/dehydrat	5,81	13,53	4,45	19,09	5,15	31,27	4,06	13,23	3,04	34,72	4,47	34,33	4,70	10,98	3,36	16,01
YP_003576338	rcc00166	sulfotransferase	5,52		4,26		6,63	24,92	5,72	23,10	5,09				2,61		4,88	
YP_003576339	rcc00167	family 2 glycosyl transferase	5,89	4,70	5,00		5,72	26,62	5,12	2,93	4,15	36,41	4,76		3,68		7,30	
YP_003576344	rcc00172	glycosyl transferase					1,18											

YP_003576345	rfbA	glucose-1-phosphate thymidylyltrans	9,10	17,83	5,81	19,77	10,19	26,83	9,10	12,85	5,87	16,71	7,12	4,40	6,62	20,55	9,50	40,62
YP_003576346	rfbD	dTDP-4-dehydrorhamnose reductase	7,93	11,62	6,05	21,49	8,69	12,23	9,00	10,78	5,98	16,21	7,00	3,57	4,97	15,95	6,04	23,01
YP_003576347	rcc00175	hypothetical protein	0,78	8,35	1,43		0,71	30,05	1,19	30,78					0,44	3,19	0,80	
YP_003576348	rfbB	dTDP-glucose 4_6-dehydratase	6,90	24,54	4,83		7,55	37,84	8,69	12,93	4,65	1,92	5,16	34,56	6,35	14,25	8,37	46,82
YP_003576349	rfbC	dTDP-4-dehydrorhamnose 3_5-epim	8,75	8,19	8,24	0,78	8,63	11,51	10,05	3,89	7,53	11,67	12,76	11,22	6,88	12,78	9,38	15,76
YP_003576351	ilvA	threonine dehydratase	2,66	1,27	2,87	31,06	2,45	21,56	1,89	21,94	1,46	20,75	2,66	6,58	1,90	18,01	2,64	20,34
YP_003576357	rcc00185	hypothetical protein	2,42				2,82								8,10	21,27	6,31	19,69
YP_003576359	hslO	chaperonin HslO	2,26	2,77	2,59	7,91	2,74	15,88	2,92	17,17	2,03	7,44	2,98	0,54	2,48	9,77	2,89	25,87
YP_003576362	rcc00190	ABC transporter ATP-binding/permea	1,31	27,49	0,82	26,65			0,76		0,92		0,62				0,57	
YP_003576365	rcc00193	SCP-like extracellular protein													2,03			
YP_003576366	rcc00194	ErfK/YbiS/YcfS/YnhG family protein/T	2,08	33,04	3,57	10,01			1,51		2,79		3,98	14,36	2,46	24,81	3,68	13,21
YP_003576367	hemH	ferrochelatase	1,84				2,22		1,04						1,47			
YP_003576370	grxC	glutaredoxin			2,66		4,99	14,68			4,22						2,94	
YP_003576371	rcc00199	carbon-nitrogen family hydrolase					0,91				0,49							
YP_003576379	lys1	saccharopine dehydrogenase	3,43	21,50	3,23	29,05	5,55	31,35	6,12	8,29	4,82	11,24	4,39	17,62	4,71	22,24	6,22	21,93
YP_003576380	argG	argininosuccinate synthase	16,42	6,71	19,47	29,88	18,86	41,36	20,74	9,05	15,02	13,95	18,63	2,51	16,59	10,50	26,79	4,14
YP_003576381	mogA	molybdopterin biosynthesis mog pro									0,84							
YP_003576382	ubiG	3-demethylubiquinone-9 3-O-methyl	5,23	18,30	3,69	15,23	5,72	0,27	4,73	1,43	3,27	20,78	4,92		3,54	4,67	4,77	
YP_003576383	pip1	prolyl aminopeptidase											1,34					
YP_003576384	rcc00212	hypothetical protein	5,78	17,02	4,33	25,50	6,92	8,57			4,71	13,66	4,20	4,52	3,13	17,89	3,42	
YP_003576385	nusA	transcription elongation factor NusA	19,04	15,61	18,17	5,56	19,61	7,03	19,45	0,99	18,48	6,63	22,47	8,16	17,15	2,13	18,14	6,04
YP_003576387	infB	translation initiation factor IF	17,34	13,68	14,19	5,56	13,70	6,50	14,95	2,59	12,69	4,60	16,16	7,31	14,23	1,63	23,21	0,13
YP_003576389	nudG	CTP pyrophosphohydrolase			1,11								2,05					
YP_003576390	argJ	arginine biosynthesis bifunctional pr	31,86	11,25	28,40	2,26	30,75	7,95	31,19	1,72	29,09	10,89	31,47	11,74	23,21	8,26	27,49	0,23
YP_003576391	rcc00219	PpiC-type peptidyl-prolyl cis-trans iso	19,16	40,80	18,35	22,56	22,75	19,47	10,86	23,90	28,27	36,62	12,37	2,31	12,76	0,12	9,52	31,90
YP_003576392	secA	preprotein translocase subunit SecA	8,29	4,22	7,19	29,18	9,99	35,31	8,08	8,74	5,75	27,56	5,75	25,66	8,49	11,75	9,63	12,69
YP_003576395	dnaJ	chaperone DnaJ			2,19		2,38	10,75	2,49	14,53	2,27	11,95	2,13					
YP_003576396	dnaK	chaperone DnaK	49,37	11,65	41,13	11,32	54,97	4,00	54,04	0,12	49,38	2,37	47,22	5,01	47,84	9,46	54,51	17,14
YP_003576400	rcc00228	DSBA family oxidoreductase	8,13	16,47	16,88	5,31	9,02	16,52	17,42	7,27	14,82	5,11	24,47	9,67	11,75	0,19	12,97	4,03
YP_003576403	rcc00231	hypothetical protein	9,33	12,13	10,83		9,78	11,02	9,83	4,07	7,49		8,88	20,89	5,57		5,41	
YP_003576404	rcc00232	xylose isomerase-like TIM barrel fam											0,81					
YP_003576407	folC	folylpolyglutamate synthase/dihydro							1,61								2,13	
YP_003576408	accD	acetyl-CoA carboxylase carboxyl tran	2,09				3,29						2,35					
YP_003576412	rcc00240	hypothetical protein	15,40	17,42	27,65	17,28	20,67	20,50	30,89	8,07	26,87	18,59	28,50	27,83	13,84	13,67	14,87	3,28
YP_003576415	dapD	2_3_4_5-tetrahydropyridine-2_6-dica	7,98	18,44	5,78	21,20	6,82	26,67	9,18	9,11	5,99	21,21	8,62	9,80	3,50	21,22	5,90	18,16
YP_003576418	mltB	membrane-bound lytic murein trans	1,76		2,18	15,77	2,34	21,99	2,16	9,91	1,58		1,67				2,17	
YP_003576421	hup1	DNA-binding protein HU	79,28	13,38	100,70	40,48	78,21	25,03	55,87	12,35	90,30	20,00	83,36	7,28	79,96	14,85	84,08	32,85
YP_003576422	amn	AMP nucleosidase	2,45	0,52	2,25	21,79	2,36	17,11	2,32	13,35	1,61	41,66	1,89	25,29	1,43	31,62	3,50	

YP_003576423	ade	adenine deaminase	3,19	9,80	2,83	25,39	3,51	14,12	3,89	2,96	2,34	19,68	4,75	23,57	3,50	8,43	3,46	2,71
YP_003576424	rcc00252	NAD-dependent epimerase/dehydrat					2,55						0,95				1,56	
YP_003576429	leuS	leucyl-tRNA synthetase	8,94	11,42	6,82	35,45	8,00	24,44	8,19	18,55	6,45	25,08	6,45	12,65	7,20	19,23	8,06	3,04
YP_003576430	rcc00258	lipoprotein	0,53															
YP_003576431	rcc00259	porin family protein	185,19	5,91	331,54	27,56	187,79	26,76	289,30	17,47	199,90	23,61	173,97	41,05	223,31	1,69	251,14	8,06
YP_003576432	rcc00260	alanine racemase domain-containing							1,17									
YP_003576434	ribA	GTP cyclohydrolase II			1,56		1,03		1,25	9,27			1,30	7,27			0,96	
YP_003576435	rcc00263	winged helix family two component t			4,54	26,54	4,83	11,23	5,42	32,97	4,67	24,04	5,07	8,79	3,69	43,73	5,99	20,70
YP_003576439	rcc00267	M16 family peptidase	9,60	16,93	9,88	14,33	8,85	7,64	8,75	5,05	6,72	4,09	9,33	8,73	6,12	2,57	7,53	26,59
YP_003576440	rcc00268	M16 family peptidase	11,73	10,66	11,86	6,14	11,89	2,93	12,39	7,74	10,08	6,48	13,87	3,57	9,60	2,58	10,36	10,68
YP_003576443	purH	bifunctional phosphoribosylaminoim	12,76	28,03	8,80	16,88	10,97	20,91	12,36	13,17	8,59	19,14	11,15	4,82	9,12	16,90	13,44	28,50
YP_003576445	rsmB1	ribosomal RNA small subunit methylt	1,55		1,47		1,61		1,99	18,29	1,79		1,46	34,68	1,75	13,19	2,14	18,58
YP_003576447	dapB	dihydrodipicolinate reductase	3,10	2,45	2,16	30,35	3,26	15,45	3,32	19,13	1,83	14,38	3,34	15,70	2,42	18,84	2,84	32,23
YP_003576448	rbfA	ribosome-binding factor A	1,59	18,25	1,86	12,52	1,52	18,94	1,39		1,66	7,60	1,94	29,10	2,26	2,54	2,23	11,37
YP_003576450	truB	tRNA pseudouridine synthase B	1,33	0,08	1,35		0,93	16,85	0,97	4,53	0,75		0,98		0,94	4,92	1,18	13,98
YP_003576453	rpsO	30S ribosomal protein S15	82,37	13,51	121,54	13,81	90,76	30,85	78,17	12,44	116,12	27,78	102,86	8,05	133,74	19,44	122,21	17,63
YP_003576454	rcc00282	aldehyde dehydrogenase			4,54	13,32	5,13	23,22	6,10	9,42	3,37	14,78	5,86	5,04	3,91	20,38	5,40	23,69
YP_003576456	pnp	polyribonucleotide nucleotidyltransf	57,34	7,61	52,70	20,60	60,58	16,18	82,00	2,85	54,35	13,14	78,14	14,67	66,70	8,75	80,32	5,45
YP_003576458	nusG	transcription antitermination protein	12,21	12,55	11,75	12,75	11,63	3,34	10,00	2,75	10,44	12,47	11,15	16,71	8,89	4,67	10,75	6,09
YP_003576459	rplK	50S ribosomal protein L11	34,66	3,85	39,45	16,74	34,18	14,33	38,62	2,55	31,66	34,82	43,44	5,55	51,80	13,29	65,31	12,06
YP_003576460	rplA	50S ribosomal protein L1	43,66	15,83	47,35	6,02	38,60	5,21	44,65	2,72	46,54	6,84	61,16	2,00	52,74	3,77	60,24	7,87
YP_003576461	rplJ	50S ribosomal protein L10	49,60	2,35	54,84	4,19	48,82	5,80	53,43	2,58	58,62	1,04	70,01	7,85	62,89	3,88	71,27	14,81
YP_003576462	rplL	50S ribosomal protein L7/L12	187,52	21,65	160,53	24,63	174,65	29,94	81,16	11,49	170,54	21,52	101,26	20,17	144,42	16,51	135,37	34,65
YP_003576463	rpoB	DNA-directed RNA polymerase subur	30,55	31,86	25,47	43,30	38,17	53,52	27,53	14,68	19,21	34,83	23,18	15,39	24,36	12,95	29,54	8,13
YP_003576464	rpoC	DNA-directed RNA polymerase beta'	21,69	3,27	21,73	13,61	28,98	6,56	35,41	5,28	23,10	12,06	29,39	12,96	26,78	8,74	32,12	18,10
YP_003576465	rcc00293	hypothetical protein	2,30	18,67			1,10	4,55	1,25	65,82	0,78							
YP_003576466	rpsL	30S ribosomal protein S12	45,13	11,28	46,54	12,57	66,42	28,32	52,04	4,49	49,48	11,41	53,63	9,08	78,09	8,54	73,23	22,39
YP_003576467	rpsG	30S ribosomal protein S7	80,46	13,81	102,64	3,08	86,69	10,26	97,50	3,88	102,11	3,07	108,96	13,16	129,15	6,27	155,62	3,14
YP_003576468	fusA1	translation elongation factor G	65,04	2,37	72,61	13,31	76,30	16,05	99,37	2,16	75,18	8,02	113,17	8,37	89,66	0,63	100,37	1,03
YP_003576470	rpsJ	30S ribosomal protein S10	61,20	24,66	83,40	8,66	67,35	29,40	80,28	5,45	92,60	12,07	96,58	7,84	94,95	12,19	95,14	28,78
YP_003576471	rplC	50S ribosomal protein L3	64,50	11,75	62,93	18,75	57,93	13,01	60,85	10,61	58,34	10,72	55,87	15,38	129,90	11,64	110,51	7,25
YP_003576472	rplD	50S ribosomal protein L4	32,96	14,43	47,78	15,46	33,47	25,76	53,99	7,17	42,81	18,51	45,54	13,71	73,24	0,55	71,82	8,88
YP_003576473	rplW	50S ribosomal protein L23	67,81	12,03	81,50	14,16	58,12	22,68	47,44	11,09	75,58	25,92	66,53	12,13	84,33	26,54	74,88	37,95
YP_003576474	rplB	50S ribosomal protein L2	38,92	6,13	41,23	8,13	44,85	16,54	33,29	5,53	43,21	7,03	42,42	6,50	74,30	8,30	65,51	3,55
YP_003576475	rpsS	30S ribosomal protein S19	44,90	13,32	80,70	22,75	69,61	41,28	51,18	13,24	83,99	32,17	75,51	6,83	94,97	20,17	96,48	29,83
YP_003576476	rplV	50S ribosomal protein L22	25,26	11,89	54,15	1,29	39,32	30,11	47,92	3,64	55,75	13,37	56,66	4,76	79,27	13,59	75,60	17,34
YP_003576477	rpsC	30S ribosomal protein S3	41,35	10,50	61,68	19,95	54,69	8,34	80,62	4,97	58,72	13,42	74,49	15,65	85,11	2,16	90,07	17,57
YP_003576478	rplP	50S ribosomal protein L16	29,12	10,61	43,39	19,78	41,99	20,72	52,35	7,54	40,42	13,50	48,39	10,86	81,35	5,64	81,04	7,33

YP_003576480	rpmC	50S ribosomal protein L29	46,48		132,15		54,02	33,51	33,36		70,13	35,39	76,89	6,56	74,83	20,25	92,89	
YP_003576481	rpsQ	30S ribosomal protein S17	18,83	18,52	24,44		36,27		16,99	12,13	27,40	24,61	30,11	8,16	34,70		28,19	36,82
YP_003576482	rplN	50S ribosomal protein L14	26,22	10,27	47,02	10,23	33,91	15,94	55,13	2,45	42,15	3,01	54,95	7,83	69,91	4,97	68,32	7,11
YP_003576483	rplX	50S ribosomal protein L24	109,28	17,78	122,63	23,81	94,23	24,85	71,71	13,67	111,70	27,82	95,94	2,72	132,25	21,26	104,45	44,96
YP_003576484	rplE	50S ribosomal protein L5	17,43	5,34	24,16	21,31	17,98	25,04	28,20	4,17	19,79	11,08	31,41	0,40	29,17	14,48	37,25	18,10
YP_003576485	rpsN	30S ribosomal protein S14			26,87	3,60	51,63		52,81		52,87		63,72	6,68	72,24	5,14	73,30	24,38
YP_003576486	rpsH	30S ribosomal protein S8	70,71	15,04	94,31	4,98	94,33	31,56	74,34	6,63	106,16	22,26	97,64	0,69	115,56	10,83	125,63	19,99
YP_003576487	rplF	50S ribosomal protein L6	29,07	17,41	53,84	5,95	35,74	16,32	52,93	5,67	47,24	4,20	59,73	3,42	68,40	5,72	74,81	4,05
YP_003576488	rplR	50S ribosomal protein L18	27,66	17,91	41,22	4,99	29,63	24,72	34,21	0,88	41,03	16,35	41,82	10,57	60,44	13,90	56,59	17,62
YP_003576489	rpsE	30S ribosomal protein S5	59,12	12,75	63,23	1,87	67,25	16,32	59,12	3,41	64,87	9,47	71,51	5,68	88,57	3,14	95,90	0,34
YP_003576490	rpmD	50S ribosomal protein L30	5,91	0,05	34,73	7,02	14,81	12,51	27,48	7,54	34,87	13,40	39,31	6,26	45,95	9,28	46,07	2,02
YP_003576491	rcc00319	hypothetical protein	5,32										4,51					
YP_003576493	rplO	50S ribosomal protein L15	58,09	11,37	74,34	5,66	52,54	15,98	55,71	7,33	73,64	12,07	66,20	15,77	101,38	15,83	81,33	29,65
YP_003576495	adk	adenylate kinase	43,14	11,66	35,29	10,64	40,39	5,03	34,99	4,34	33,04	4,33	30,62	6,71	24,38	4,21	32,17	8,15
YP_003576496	rpsM	30S ribosomal protein S13	42,29	14,82	48,52	4,70	51,94	26,23	38,77	5,85	52,52	11,57	54,29	3,00	63,00	2,56	70,63	17,98
YP_003576497	rpsK	30S ribosomal protein S11	27,17	16,18	28,54	6,09	27,88	13,21	23,45	8,98	21,96	6,39	29,90	9,07	33,03	2,73	40,10	13,92
YP_003576498	rpoA	DNA-directed RNA polymerase subur	50,60	8,18	43,69	5,90	54,39	8,03	53,71	3,39	46,89	5,42	53,31	2,21	43,08	1,54	55,19	7,88
YP_003576499	rplQ	50S ribosomal protein L17	18,55	14,87	20,81	17,30	21,25	16,79	23,91	4,25	22,19	4,39	25,77	15,19	41,54	1,21	42,11	9,74
YP_003576500	rcc00328	LuxR family autoinducer-binding tran	3,95	24,50	2,62	36,69	5,04	36,40	4,56	16,12	3,83		3,82		3,32	31,72	4,44	13,82
YP_003576504	rluC	ribosomal large subunit pseudouridir	1,89	0,39	2,26	25,96	1,72	16,86	1,51		1,58						2,76	
YP_003576506	rcc00334	ATP12 chaperone protein family					2,80	13,79	4,78		2,46	35,14	2,91	19,60	2,26		2,60	13,83
YP_003576507	bztA	glutamate/aspartate ABC transporte	0,89		0,87		1,61		1,45	0,40	0,51		1,29		2,67	5,68	3,82	14,16
YP_003576510	bztD	glutamate/aspartate ABC transporte	2,42		1,83	24,70	2,03				1,57		1,97				1,97	
YP_003576511	sixA	phosphohistidine phosphatase	9,90	17,51	10,50	8,68	13,06	6,15	12,87	1,81	10,76	6,39	12,83	1,53	8,44	9,70	10,75	18,63
YP_003576513	argB	acetylglutamate kinase	5,93	5,29	4,92	25,34	6,36	15,43	6,78	15,73	5,32	29,02	4,98	10,53	4,82	15,96	5,67	5,10
YP_003576514	rcc00342	short-chain dehydrogenase/reductas	3,61	16,83	2,11	15,64	3,25	2,63	4,27	9,98	2,39		3,34	3,90	4,08	43,58	3,50	24,56
YP_003576517	oxaA	inner membrane protein OxaA	4,42	7,00	3,66	15,43	2,93	33,12	2,62	2,96	1,89	28,75	2,55		1,95	15,32	3,32	9,57
YP_003576526	pepT	peptidase T	4,48	5,70	3,61	19,12	4,10	7,33	4,72	16,72	3,42	17,10	4,94	8,45	3,75	6,24	4,31	18,45
YP_003576527	rcc00355	S1 RNA binding domain-containing p	2,58	19,97	5,57	39,18			3,40	57,08	1,47	11,03	1,81	28,12	2,12	3,28	3,26	7,08
YP_003576532	mipZ	chromosome partitioning protein Mi									0,66							
YP_003576533	rpmE	50S ribosomal protein L31	33,09	19,43	53,86	17,08	22,39	4,40	36,17	7,39	50,76	13,74	61,12	8,93	43,66	9,80	47,93	39,64
YP_003576534	rplS	50S ribosomal protein L19	29,27	8,53	55,61	12,99	37,27	14,22	44,33	3,99	50,72	17,07	60,57	13,31	68,09	15,05	65,36	11,35
YP_003576539	rpsP	30S ribosomal protein S16	176,90	15,42	257,81	37,95	177,36	28,61	126,05	16,47	210,91	32,73	163,26	2,64	194,26	28,01	176,99	29,17
YP_003576542	ffh	signal recognition particle protein	2,09	1,36	2,19		1,81		2,30	1,51	1,30		2,35	4,40	2,46		2,07	
YP_003576545	mdoH	glucans biosynthesis glucosyltransfer	0,76						1,31									
YP_003576547	mdoG1	glucans biosynthesis protein G	7,37	10,67	6,11	20,27	9,52	19,71	9,12	9,59	6,10	26,70	6,52	2,69	6,06	15,26	9,19	40,58
YP_003576550	glnA1	glutamine synthetase															0,74	
YP_003576551	dapA	dihydrodipicolinate synthase	10,57	6,10	9,50	18,32	11,42	14,70	12,11	16,02	11,04	4,62	14,45	8,14	9,51	15,10	13,22	7,59

YP_003576554	rcc00382	hypothetical protein							1,32								0,52	
YP_003576555	rcc00383	FAD dependent oxidoreductase	2,79	6,11	2,78	9,32	3,01	1,24	3,48	16,59	2,42	40,82	5,63	89,95	3,73	15,53	4,26	19,49
YP_003576556	gshA	glutamate--cysteine ligase	2,61	0,72	2,87	15,36	2,58	22,19	2,33	13,96	3,38		2,96	12,33	1,71	12,75	2,16	9,02
YP_003576558	rcc00386	hypothetical protein	1,03	40,63	1,05	25,93	1,19		1,33	4,48					1,56			
YP_003576560	rcc00388	OmpA/MotB domain-containing prot	1,97		2,66		2,79		2,03	16,03	1,46	13,77			1,31		1,38	
YP_003576561	rcc00389	hypothetical protein									1,60							
YP_003576569	pyrC1	dihydroorotase	4,30	3,20	3,57	46,73	3,87	23,48	5,14	15,89	3,51	30,91	3,67	16,57	3,76	18,49	4,21	1,36
YP_003576572	pyrB	aspartate carbamoyltransferase	3,09	2,70	3,16	4,38	3,61	17,59	3,77	23,70	2,88	24,76	3,31	5,01	3,03	14,20	3,59	2,63
YP_003576574	rcc00402	hypothetical protein															0,81	
YP_003576576	rcc00404	Sua5/YciO/YrdC/YwlC family protein	1,87	29,11	1,21	14,01	1,39	49,52	2,19	46,51	2,07	12,02	1,43	20,24	1,32	27,37	1,51	1,50
YP_003576577	rcc00405	acyl-CoA dehydrogenase domain-con	1,96		2,01		2,03	7,21	2,49	5,38			1,79				1,36	
YP_003576579	rcc00407	lipoprotein			0,33													
YP_003576584	rcc00412	arylformamidase	3,32	29,55	2,04	1,90	2,31	12,13	2,33	8,62	2,09	10,28	3,02	10,10	2,18	10,84	2,33	7,45
YP_003576585	gabD	succinate-semialdehyde dehydrogen	12,03	7,10	9,72	18,70	14,77	15,00	15,90	9,66	10,39	16,42	11,21	8,32	7,03	26,90	8,91	21,37
YP_003576587	rcc00415	short-chain dehydrogenase/reductas	5,71	7,38	5,43	8,61	5,83	16,12	6,71	11,73	4,55	14,28	6,25	17,54	4,93	17,69	5,91	19,66
YP_003576592	pykA1	pyruvate kinase	2,02	39,16							0,82				1,03		1,21	
YP_003576593	lysC	aspartate kinase	13,39	3,62	10,44	3,07	13,11	3,03	12,64	6,29	11,41	3,25	11,93	12,60	8,94	15,61	11,56	17,90
YP_003576594	ptsP	phosphoenolpyruvate-protein phosp					2,31	19,74			0,61		1,49	16,57				
YP_003576599	panC	pantoate--beta-alanine ligase			1,96				3,22									
YP_003576600	panB	3-methyl-2-oxobutanoate hydroxyme	2,29	22,34	1,33		1,95	20,14	2,77	18,69	1,48				1,69		1,51	
YP_003576601	rcc00429	hypothetical protein							1,86									
YP_003576602	rcc00430	ErfK/YbiS/YcfS/YnhG family protein													1,84			
YP_003576604	mdtB	multidrug resistance protein MdtB			2,25													
YP_003576605	rcc00433	RND family efflux transporter subuni	2,86				3,03	8,27	2,96									
YP_003576610	glyA	serine hydroxymethyltransferase	21,57	17,85	17,92	15,11	22,03	15,82	26,78	2,04	18,16	10,51	24,09	2,39	18,56	11,98	24,91	23,17
YP_003576614	prpE	propionate--CoA ligase											12,74		3,33			
YP_003576615	maeB1	malate dehydrogenase													1,72			
YP_003576616	cdd	cytidine deaminase	8,26		7,18	34,48	6,93	31,42	4,37	4,59	3,62	5,65	6,78	22,56	2,78	16,44	3,53	29,56
YP_003576617	deoA	thymidine phosphorylase	1,92	8,35	1,91	33,82	2,45	17,96	2,38	46,21	2,56		1,33	3,52	1,31		2,14	35,88
YP_003576618	deoB	phosphopentomutase					2,57										1,02	
YP_003576619	upp	uracil phosphoribosyltransferase	10,95	7,82	10,30	29,72	12,24	17,27	14,94	13,22	9,69	34,86	10,79	21,35	10,64	16,69	13,43	9,27
YP_003576621	rcc00449	hypothetical protein	0,55		1,02		1,28		0,96		1,22						1,22	
YP_003576625	rcc00453	amino acid ABC transporter periplasr	7,70	16,54	10,79	24,06	9,76	23,50	8,72	2,81	8,94	4,55	8,60	24,59	5,02	1,76	5,40	
YP_003576627	dut	deoxyuridine 5'-triphosphate nucleot	5,46	55,65	5,40	38,36	5,62	40,49	2,46				2,04	9,88			9,50	
YP_003576629	rcc00457	hypothetical protein									1,39							
YP_003576630	rpoH1	RNA polymerase sigma-32 factor							9,04				11,34					
YP_003576631	rcc00459	glutathione S-transferase	4,94	22,07	6,23	30,07	7,10	18,66	8,47	8,69	5,94	2,84	11,25	2,07	3,24	33,83	7,45	29,57
YP_003576634	gshB	glutathione synthase	3,25	4,81	3,43	22,07	4,49	18,44	5,17	31,21	2,89	22,64	5,38	9,88	3,39	36,36	3,80	11,78

YP_003576637	rcc00465	extracellular ligand-binding receptor	2,80	0,59	2,94	17,45	2,60	34,24	2,85	9,84	2,00	19,75	2,44	17,68	2,91	16,92	3,07	19,86
YP_003576641	trpS	tryptophanyl-tRNA synthetase	5,92	4,09	4,23	26,26	5,69	21,95	5,77	18,84	4,20	28,36	4,27	15,26	5,17	18,35	6,40	24,50
YP_003576643	rcc00471	thioesterase superfamily protein							2,14	14,36			1,81					
YP_003576644	rcc00472	thioesterase superfamily protein									0,52							
YP_003576645	rcc00473	MerR family transcriptional regulator					1,60						1,88					
YP_003576646	rcc00474	MerR family transcriptional regulator					1,99											
YP_003576647	rcc00475	acyl-CoA dehydrogenase domain-con	2,70	8,54	2,02		2,43	13,26	2,10		2,10	25,90						
YP_003576648	rcc00476	hypothetical protein	13,38	27,12	14,97	14,46	19,05	13,09	13,63	3,26	13,74	9,35	15,97	2,13	12,03	7,72	16,12	5,31
YP_003576650	rcc00478	alcohol dehydrogenase							1,62		1,08	10,08	1,82	5,52				
YP_003576651	rcc00479	COQ9 family ubiquinone biosynthesis	11,37		1,41	17,35	6,54	57,13	1,99	1,87	1,02		1,57		1,97	20,81	2,56	25,92
YP_003576652	rpsU	30S ribosomal protein S21			18,63												18,06	
YP_003576656	ald	alanine dehydrogenase	8,16	19,41	4,64	18,73	10,62	17,68	9,70	12,25	7,61	13,62	6,91	12,61	4,61	21,22	7,93	21,92
YP_003576659	suhB	inositol-1-monophosphatase	6,81	8,36	5,51	21,79	6,87	28,05	6,72	19,47	5,03	19,95	19,09	86,95	16,92	53,79	9,47	27,31
YP_003576662	metF	5_10-methylenetetrahydrofolate red	3,82	2,52	3,34	34,32	3,76	18,73	4,26	1,93	2,64	1,68	3,48	6,48	7,49	18,64	6,04	13,46
YP_003576665	ldc	lysine/ornithine decarboxylase	11,80	22,82	8,12	17,02	16,10	24,65	13,92	5,22	10,98	9,58	11,52	0,35	7,07	19,14	7,54	13,16
YP_003576668	valS	valyl-tRNA synthetase	9,12	21,24	6,45	15,24	9,80	29,26	9,21	9,79	6,30	16,73	7,28	2,37	7,02	17,42	7,86	16,34
YP_003576674	rcc00502	type II and III secretion system prote	3,57		1,75		1,52				2,06		2,04	6,43				
YP_003576675	rcc00503	OmpA/MotB domain-containing prot					0,79		1,05		0,14		0,28	32,05				
YP_003576683	ispE	4-diphosphocytidyl-2C-methyl-D-eryt	3,53	0,01	3,21	14,22	3,92	4,70	4,02	12,06	3,00	10,64	4,35	6,36	3,31	7,64	3,38	4,73
YP_003576684	rcc00512	hypothetical protein	4,43	5,74	4,51	16,60	5,17	35,72	3,99	16,70	3,44	9,85	5,63	6,78	4,31	8,00	5,42	11,37
YP_003576685	etfD	electron transfer flavoprotein-ubiqui	6,22	4,80	3,28	21,80	5,10	5,40	4,32				3,70					
YP_003576686	greA	transcription elongation factor GreA	22,69	19,44	20,21	38,23	23,03	27,46	11,92	8,02	17,49	26,67	16,72	17,13	13,46	5,77	14,21	22,49
YP_003576688	tpl	tyrosine phenol-lyase	6,30	1,57	5,73	27,21	4,49	13,01	5,49	13,97	5,31	31,69	6,53	7,20	4,65	19,31	7,06	14,54
YP_003576690	fadA	fatty acid oxidation complex subunit	0,82		1,43	19,04	1,52	14,30	1,63	11,54	1,15		1,39	11,95	2,95		0,49	
YP_003576692	fadB	fatty acid oxidation complex subunit			1,21	5,47	1,63		1,69	27,33			0,85					
YP_003576693	dcp	peptidyl-dipeptidase Dcp	2,09	7,92	2,16	11,14	2,15	17,18	2,36	10,96	2,07	19,90	2,28	16,66	1,65	8,55	2,12	23,38
YP_003576696	gyaR1	glyoxylate reductase	1,96				3,99		3,16		1,65		1,94	19,84				
YP_003576703	carB	carbamoyl-phosphate synthase large	11,68	12,33	11,31	19,43	11,91	19,21	14,93	10,96	12,34	18,73	13,66	7,49	13,17	12,27	15,72	13,13
YP_003576704	aspS	aspartyl-tRNA synthetase	12,59	6,39	9,52	27,00	10,82	15,74	12,60	16,78	8,72	21,88	12,25	6,75	11,97	16,41	15,48	23,71
YP_003576705	rcc00533	hypothetical protein					1,40		2,54	1,45	2,26		2,97		0,77		1,38	
YP_003576706	rcc00534	hypothetical protein			3,10	12,16	1,59				2,08	21,96	4,02	27,15			1,44	
YP_003576708	rcc00536	GNAT family acetyltransferase	2,01	21,68	2,07	23,39	2,15	34,61	2,60	31,28	2,97		2,87	5,69	1,25	15,72	2,89	23,60
YP_003576710	rcc00538	methylmalonyl-CoA epimerase					4,57	21,58			3,62	7,53						
YP_003576712	rcc00540	nitroreductase	5,74	8,83	4,59	16,78	6,34	19,53	7,02	17,23	4,47	31,44	4,64	35,14	5,09		4,97	
YP_003576715	rcc00543	hypothetical protein	4,21	4,91	3,91	8,25	5,01	3,09	4,24		3,48						4,22	
YP_003576716	rcc00544	aldehyde dehydrogenase			1,81		2,38	10,59	1,68	22,05			1,22					
YP_003576719	rcc00547	glutaredoxin family protein	21,23	5,44	14,30	7,90	23,19	13,52	12,73	2,45	17,89	5,93	15,69	5,86	22,42	1,00	15,96	8,20
YP_003576720	rcc00548	BolA family protein	12,04	1,97	8,88	24,56	12,03	28,13			10,57	20,82	6,55	8,64	6,71		5,76	

YP_003576721	purL	phosphoribosylformylglycinamidine s	13,25	7,15	12,82	25,80	15,77	12,14	19,02	10,66	14,10	27,83	14,13	21,71	14,98	9,90	15,84	9,88
YP_003576722	rcc00550	LysR family transcriptional regulator	2,40	20,50	3,75	2,05	2,21	14,03	3,77	14,85	2,00	11,85	4,03	1,31	3,08	17,13	3,57	14,87
YP_003576723	murI	glutamate racemase	3,97	11,59	5,73	17,98	5,53	22,49	7,17	12,77	4,74	26,32	6,39	8,90	4,63	14,66	5,88	23,33
YP_003576725	argC	N-acetyl-gamma-glutamyl-phosphate	5,49	24,68	4,78	18,72	6,54		6,61	16,56	4,23	30,86	6,11	7,74	4,00	19,88	6,75	38,97
YP_003576726	ccmE	cytochrome c-type biogenesis protei	4,26	12,43	3,04	15,41	3,05	22,93	3,35	38,15	2,74	39,41	5,18		1,60		3,29	
YP_003576727	rcc00555	hypothetical protein			10,30		4,63											
YP_003576729	metG	methionyl-tRNA synthetase	3,67	9,67	3,51	6,14	4,35	22,59	4,41	16,10	3,18	32,56	3,12	12,85	3,78	12,24	4,65	27,71
YP_003576731	pmtA	phosphatidylethanolamine N-methyl			3,28	15,25			2,18				3,10		8,81	10,84	5,10	9,73
YP_003576732	mopB	molybdenum transport operon repre	1,85	66,75	2,47	53,84	0,84		1,56	5,67	0,73	42,82	1,80	29,62				
YP_003576733	mopA	molybdenum transport operon repre	15,90	8,99	3,09		6,16	18,72			13,72	25,85	2,89	18,63				
YP_003576734	modA1	molybdenum ABC transporter peripla	163,61	21,44	15,03	74,42	90,78	36,79	7,53		648,23	28,66	57,92	50,77	5,60	7,11	7,59	16,98
YP_003576737	modD	molybdenum utilization protein Mod	3,31	10,86			2,79	12,03			4,26	18,39	1,55					
YP_003576738	nifB1	nitrogenase cofactor biosynthesis pr													3,67	14,40		
YP_003576740	rpoN	RNA polymerase sigma-54 factor			2,68													
YP_003576742	nifK	nitrogenase molybdenum-iron prote	108,83	10,29	277,33	21,76			1,55		97,80	31,01	262,31	7,22	261,51	14,86	193,78	6,65
YP_003576743	nifD	nitrogenase molybdenum-iron prote	62,30	10,87	234,85	15,84	8,32				53,92	25,06	216,91	3,44	171,92	9,87	140,83	0,44
YP_003576744	nifH1	nitrogenase iron protein	325,32	7,86	404,48	7,39	13,24		1,52		366,84	11,38	434,05	1,86	389,96	4,70	239,90	6,13
YP_003576745	fdxD	ferredoxin V	17,15	5,00	22,91	22,08					39,04	10,49	19,01	20,39	6,42	0,39	12,44	
YP_003576756	anfA	nitrogen fixation regulatory protein A													2,75			
YP_003576757	anfH	nitrogenase iron protein	329,69	6,69	1,22	43,32					0,43		1,23		466,53	1,35	56,83	14,76
YP_003576758	anfD	nitrogenase iron-iron protein subuni	37,36	23,14									3,24		79,56	13,07	2,39	
YP_003576759	anfG	nitrogenase iron-iron protein subuni	12,34	7,18											11,25	5,09		
YP_003576760	anfK	nitrogenase iron-iron protein subuni	76,28	15,61	2,48	37,70							3,25		113,13	19,30	17,41	12,66
YP_003576761	anfO	nitrogenase iron-iron accessory prot	2,93	28,29											12,52	14,22		
YP_003576762	rcc00590	hypothetical protein													6,75			
YP_003576763	rcc00591	flavin-nucleotide-binding protein	34,74	10,02	2,91								2,62		48,63	19,50	7,29	12,37
YP_003576766	rcc00594	MoxR family ATPase	8,05	0,58	5,59	35,73	7,13	32,45	7,29	14,38	4,29	42,64	5,34	17,78	7,83	36,57	7,80	32,35
YP_003576767	rcc00595	hypothetical protein									0,66							
YP_003576769	rcc00597	hypothetical protein	0,77		1,30	35,01	0,80	11,02	1,37	59,77			0,94		1,10		0,80	
YP_003576772	nrdJ1	ribonucleoside-diphosphate reducta	2,03				3,01		2,75	17,18	1,87	4,03	2,70	0,21	2,11		1,71	
YP_003576783	nikR	nickel-responsive regulator NikR							0,62									
YP_003576787	acrA	acriflavine resistance protein A	7,43	7,60	5,94	17,14	6,81	11,84	4,41	10,41	3,72	6,88	3,11	20,58	4,27	13,48	4,28	13,52
YP_003576789	rcc00617	hemolysin D	3,15	2,13	3,30	15,45	3,47	18,59	3,64	43,52	3,26	20,13	3,03	28,13	1,63		1,91	
YP_003576794	moeA	molybdopterin biosynthesis protein	16,40	3,65	11,23	16,84	14,07	13,95	12,43	5,64	11,56	1,25	12,93	7,36	14,61	14,92	16,50	19,93
YP_003576796	rcc00624	amino acid ABC transporter periplasm	4,04	21,92	2,65	11,10	4,80	26,04	2,90	1,96	3,85	6,20	2,99	17,85	2,45	2,31	2,06	
YP_003576800	menC	O-succinylbenzoic acid synthetase	0,69						0,87				0,34					
YP_003576809	rcc00637	hypothetical protein					0,93											
YP_003576810	srfB	virulence protein SrfB			1,29	25,73	2,39	64,77					1,09		1,82			

YP_003576817	rcc00645	diguanylate cyclase/phosphodiestera							2,36	14,36			1,25					
YP_003576820	kdtA3	3-deoxy-D-manno-octulosonic-acid t															0,07	
YP_003576826	rcc00654	hypothetical protein															1,47	
YP_003576831	puhA	photosynthetic reaction center subu	51,75	8,93	50,29	25,77	39,59	31,31	37,90	15,78	29,31	31,42	22,53	54,12	46,66	2,03	53,76	3,71
YP_003576834	bchL	light-independent protochlorophylli			2,06						2,93		2,28	7,29	4,92	10,62	6,73	20,73
YP_003576835	bchH	magnesium chelatase H subunit	1,83												3,42	15,80	4,06	42,30
YP_003576836	bchB	light-independent protochlorophylli	2,12	26,61	2,12	19,61	1,52	4,42	2,02	17,33	1,67	1,95	1,84	6,83	4,35	21,29	5,54	4,29
YP_003576837	bchN	light-independent protochlorophylli	1,79	2,70	1,66	44,14	1,31	0,37	1,51		1,83	11,20	2,05	36,93	4,00	6,49	5,62	27,50
YP_003576840	ppsR	transcriptional regulator PpsR	1,73		0,92	24,55			1,12	30,87								
YP_003576841	bchE	magnesium-protoporphyrin IX mono	4,88	10,56	4,99	32,25	3,19	23,80	4,30	15,31	4,16	20,49	5,07	17,74	21,22	10,65	23,87	7,30
YP_003576845	bchP	geranylgeranyl reductase	3,17	6,15			3,56		1,32						5,13	26,97	8,42	44,66
YP_003576846	idi2	isopentenyl-diphosphate delta-isome															1,36	
YP_003576847	bchO	magnesium-chelatase BchO			0,54													
YP_003576849	bchI	magnesium chelatase ATPase subuni	4,57	0,37	4,92	20,23	4,62	2,73	6,03	6,38	5,21	4,59	6,35	11,74	9,15	4,84	12,73	30,13
YP_003576850	crtA	spheroidene monooxygenase	1,71		2,51	21,17	2,56	21,90	2,44		1,34		2,07	36,97	2,91	30,79	3,32	3,08
YP_003576851	crtI	phytoene dehydrogenase	5,66	5,81	4,22		4,22				2,99		3,76	49,99	9,60	9,03	9,44	18,33
YP_003576856	crtE	farnesyltranstransferase	2,40		3,74	55,96	1,18	28,47	2,17	3,59	1,70		1,96	0,33	2,68	15,60	3,53	16,23
YP_003576857	crtF	hydroxyneurosporene methyltransfe													1,16	4,20	1,65	
YP_003576858	bchC	2-desacetyl-2-hydroxyethyl bacterio			1,91		1,71	7,24	2,27		2,20	13,86	1,90		4,52	21,21	5,81	20,93
YP_003576859	bchX	chlorophyllide reductase subunit Bch	1,22		1,81		2,37		2,83		1,22		2,77	42,55	5,34	13,96	6,17	15,47
YP_003576860	bchY	chlorophyllide reductase subunit Bch													1,50		3,04	26,87
YP_003576861	bchZ	chlorophyllide reductase subunit Bch													1,64	8,86	2,14	
YP_003576865	pufL	photosynthetic reaction center subu	16,36	3,55	14,65	3,72	13,37				8,21						6,82	
YP_003576866	pufM	photosynthetic reaction center subu	13,31	19,59	10,58	12,95	3,63		4,66	34,61	2,55	44,41	5,94		7,68		8,71	
YP_003576868	dxs1	1-deoxy-D-xylulose-5-phosphate syn							1,04				1,38	24,10			0,64	
YP_003576873	bsaA1	glutathione peroxidase	2,37		2,50	7,03	4,02	17,23	4,79	4,93	3,07	24,90	2,22	12,54	1,62			
YP_003576878	oppA1	oligopeptide ABC transporter peripla	7,25	16,22	55,00	11,40	18,52	32,41	94,00	6,74	39,37	37,66	49,08	11,18	15,15	12,62	18,54	7,90
YP_003576879	oppF	oligopeptide ABC transporter ATP-bi			1,96	22,85	3,16	7,58	2,53	14,28			1,75		2,95			
YP_003576881	rcc00709	FAD dependent oxidoreductase	2,99		2,30		2,65	25,99	1,89	3,63	1,32				1,12		1,90	
YP_003576882	hipO	hippurate hydrolase	4,67	15,49	4,21	12,56	6,02	11,39	6,51	6,97	4,29	18,78	6,00	8,75	4,36	9,19	4,34	13,26
YP_003576883	rcc00711	universal stress family protein					1,91	10,00			2,51	49,27						
YP_003576885	kynU	kynureninase	3,80	3,96	3,55	21,82	3,77	20,42	3,96	12,43	2,83	13,40	4,46	7,71	3,66	14,74	4,20	6,01
YP_003576887	pntA	pyridine nucleotide transhydrogenas	7,75	6,94	5,56	21,83	7,27	8,06	5,22	6,44	3,48	29,50	5,26		2,78		6,75	32,88
YP_003576890	mdh	malate dehydrogenase	53,85	12,82	49,56	11,65	56,71	19,35	60,71	8,44	53,18	26,74	56,92	7,82	70,23	11,80	67,76	19,79
YP_003576892	sucC	succinyl-CoA synthetase (ADP-formin	13,76	4,01	12,00	18,17	14,62	15,48	17,88	4,97	13,48	15,35	16,44	6,68	12,43	7,94	13,78	23,27
YP_003576893	sucD	succinyl-CoA synthetase (ADP-formin	12,70	16,59	10,69	7,22	13,31	7,75	13,77	7,92	11,31	6,03	14,81	5,22	8,38	12,74	10,59	16,41
YP_003576896	sucA	oxoglutarate dehydrogenase	7,55	11,67	5,94	24,94	7,79	25,17	8,15	14,14	5,97	27,65	6,66	11,17	6,25	15,84	7,77	36,18
YP_003576897	sucB	dihydrolipoyllysine-residue succinylt	10,99	8,51	8,01	31,20	9,40	13,06	10,20	6,95	7,87	22,27	7,53	23,43	8,00	15,43	8,85	22,22

YP_003576898	lpdA1	dihydrolipoyl dehydrogenase	3,48	23,67	3,25		4,92	71,56	3,17	6,75	2,54	69,03	3,07	5,35	3,28		3,33	28,89
YP_003576902	maoC	MaoC domain-containing protein	6,05	21,42	3,67	7,77	4,27	28,66	4,69	15,53	3,90	23,03	4,30	3,26	4,22	10,76	5,09	25,14
YP_003576905	sdhA	succinate dehydrogenase_ flavoprote	14,32	8,01	8,47	26,34	12,12	36,30	9,80	6,86	6,12	31,48	6,00	46,66	7,40	10,51	8,01	23,98
YP_003576908	sdhB	succinate dehydrogenase_ iron-sulfu	4,01	1,94			4,62	20,38	2,85	21,25			3,23				2,19	2,21
YP_003576910	rcc00738	hypothetical protein			0,75		2,19		2,00	13,53	1,43	24,03						
YP_003576915	atpX	ATP synthase F0 subunit B'	33,33	4,01	24,92	20,85	22,15	33,44	20,21	10,42	16,23	27,17	13,64	32,19	21,89	3,41	23,89	18,21
YP_003576916	atpF	ATP synthase F0 subunit B	17,91	9,31	12,32	20,06	12,76	21,84	10,70	18,69	8,62	21,46	8,57	18,98	9,86	0,48	12,09	18,72
YP_003576917	phaZ	polyhydroxyalkanoate depolymerase	3,24	3,64	3,40	8,77	4,16	20,71	2,32	10,52	2,46	23,48	2,96	3,64	1,87		3,20	18,17
YP_003576918	phbC	poly(3-hydroxyalkanoate) polymeras	4,14	0,12	3,19	37,65	3,72	20,54	3,11	4,40	2,80	8,20	2,95	9,06	2,47	16,87	3,17	41,67
YP_003576919	rcc00747	hypothetical protein	12,65	17,48	13,11	21,26	22,28	26,54	11,25	24,51	12,44	14,16	8,78	25,11	5,04	5,46	11,86	0,57
YP_003576920	phaR	polyhydroxyalkanoate synthesis repr	10,85	10,92	11,10	10,24	15,34	14,69	14,11	6,51	9,88	16,27	10,85	14,59	4,46	0,21	7,01	4,98
YP_003576922	pyrG	CTP synthase	5,76	9,22	3,77	30,08	6,23	32,83	5,10	9,96	4,36	29,96	4,49	11,29	4,60	2,87	5,61	5,15
YP_003576924	purA	adenylosuccinate synthetase	6,10	2,39	7,46	16,98	4,51	26,59	10,02	8,61	7,56	14,88	11,00	8,82	8,25	13,70	11,18	16,69
YP_003576926	rcc00754	lipoprotein			0,87													
YP_003576927	thiN	thiamine pyrophosphokinase					1,24	24,48	1,28									
YP_003576929	rcc00757	alpha/beta fold family hydrolase	6,63	1,02	5,54	24,62	7,44	10,46	8,64	6,00	5,80	12,48	6,25	14,65	4,32	17,12	6,22	45,95
YP_003576931	mcpB	methyl-accepting chemotaxis protein	1,97															
YP_003576940	hupB	hydrogenase large subunit	10,46	15,40	9,22	17,17	4,38	20,38	3,86	2,04	7,85	26,80	9,51	9,18	5,14	10,24	7,66	33,56
YP_003576942	hupD	hydrogenase maturation protease Hu									0,62							
YP_003576944	hupG	hydrogenase expression/formation p	1,27															
YP_003576945	hupH	hydrogenase expression/formation p	1,93	13,68	2,56	23,60	0,52				3,19	31,27	3,20	7,90	0,97		1,27	
YP_003576953	hypE	hydrogenase expression/formation p			0,86	21,37					1,62		1,93		0,41		0,48	
YP_003576954	cheR1	chemotaxis protein methyltransferas											1,83					
YP_003576957	sqr	sulfide:quinone oxidoreductase			1,55												0,87	
YP_003576958	rcc00786	basic membrane lipoprotein family	69,11	2,16	77,92	12,19	96,50	6,83	144,30	3,34	87,48	16,42	85,52	5,27	60,35	5,58	64,87	19,50
YP_003576967	hisS	histidyl-tRNA synthetase	3,86	7,44	3,65		3,43		3,49		1,94	10,43	2,78	8,00				
YP_003576968	hisZ	ATP phosphoribosyltransferase regul	1,52	16,21	1,31		1,26	13,35					1,55				1,37	
YP_003576975	mreB	rod shape-determining protein MreB			4,80	18,36	2,03		6,07	1,55	4,54	23,36	5,73	2,76	3,55	2,23	3,93	2,96
YP_003576978	rcc00806	heat shock protein DnaJ domain-con											0,83					
YP_003576981	ppx	exopolyphosphatase	2,62		1,41		1,92	9,41	2,16	28,89	1,45				1,00		0,98	
YP_003576982	ppk	polyphosphate kinase	7,14	19,48	4,69	18,84	6,13	11,80	6,26	14,04	4,98	19,00	5,71	7,59	5,79	14,37	7,03	13,23
YP_003576985	rcc00813	hypothetical protein	1,82		2,80	10,57	3,75		4,00	4,38	3,24		3,78	19,27	2,43	8,39		
YP_003576989	ldh	L-lactate dehydrogenase	12,13	34,53	5,47	47,39	14,36	30,47	9,96	22,66	9,01	42,39	6,27	38,22	8,53	5,80	5,50	19,30
YP_003576991	murG	undecaprenyldiphospho-muramoylp	2,65	23,20	2,92	17,48	2,92	7,97	2,22	18,79	1,79	11,10	2,07				1,55	59,54
YP_003576992	murC	UDP-N-acetylmuramate--L-alanine lig	3,71	5,53	3,24	23,98	4,02	23,43	4,07	13,19	3,47	2,17	3,91	3,83	3,00		3,67	13,53
YP_003576995	ddl	D-alanine--D-alanine ligase									1,09							
YP_003576997	ftsA	cell division protein FtsA							0,81				0,68					
YP_003576998	ftsZ	cell division protein FtsZ	6,91	10,36	6,83	8,88	7,68	9,06	8,24	11,60	6,17	8,02	8,21	6,53	6,88	6,30	7,53	19,25

YP_003577000	comL	competence lipoprotein ComL			0,89						0,83		1,37					
YP_003577002	rcc00830	hypothetical protein															0,18	
YP_003577003	dld	D-lactate dehydrogenase			1,47	20,13			1,87		1,64	30,72	2,01	0,83			1,57	
YP_003577004	pta	phosphate acetyltransferase	25,21	8,38	21,87	24,11	12,29	12,22	16,17	11,74	19,27	30,65	22,88	12,83	20,77	9,65	26,09	4,42
YP_003577005	ackA1	acetate kinase	9,85	13,67	11,35	28,82	5,94	12,94	9,50	15,98	9,50	30,14	11,19	15,21	12,49	15,02	14,34	12,40
YP_003577006	pepP	aminopeptidase P	3,22	3,62	2,74	26,95	3,43	17,99	3,58	18,43	2,74	34,55	2,75	12,41	4,08	35,48	2,37	25,17
YP_003577007	cobT	cobaltochelatase subunit CobT	2,42		2,22		2,54	0,11	2,50	1,44	1,29	17,73			1,68		1,73	25,08
YP_003577008	cobS	cobaltochelatase subunit CobS	13,15	6,97	14,74	27,54	14,68	9,86	17,40	12,93	16,58	1,19	15,12	8,82	19,17	2,95	19,70	7,03
YP_003577009	rcc00837	hypothetical protein	3,02	18,21	2,96	8,93	2,23	8,51	1,55	0,93			1,72		1,64		1,91	12,50
YP_003577010	rcc00838	DnaJ domain-containing protein			1,50	31,08					1,93							
YP_003577011	glcB	malate synthase	9,96	13,42	10,95	17,64	8,85	14,52	11,38	13,22	7,99	18,88	16,42	10,40	8,75	15,15	11,49	17,85
YP_003577012	pepN	aminopeptidase N	6,72	68,67	3,24	23,86	4,44	35,52	4,37	13,36	2,65	21,42	3,72	11,21	3,74	8,63	4,06	11,00
YP_003577013	gatB	glutamyl-tRNA(Gln) amidotransferase	9,94	2,41	7,18	24,69	8,52	14,31	8,75	8,29	7,19	17,01	10,09	28,10	7,27	2,83	9,20	17,45
YP_003577018	dppA	dipeptide ABC transporter periplasm	5,22	8,16	13,61	15,32	9,90	25,08	38,81	6,89	8,23	51,63	13,21	7,65	8,46	16,24	8,81	17,02
YP_003577023	rcc00851	membrane dipeptidase	1,55		1,72		3,21	32,42	5,32	9,83	1,09		2,11				1,35	
YP_003577024	srmB	ATP-dependent RNA helicase SrmB	10,34	26,49	6,16	21,51	9,31	8,17	9,28	4,36	7,08	11,30	10,20	8,70	7,48	8,11	9,25	15,81
YP_003577026	argS	arginyl-tRNA synthetase	7,96	5,54	6,06	23,90	7,48	13,28	7,97	14,02	5,79	21,55	7,24	5,21	6,82	5,92	8,18	32,77
YP_003577028	nagZ	beta-N-acetylhexosaminidase	0,89	22,39			1,19	16,42			0,80	33,56	1,31		0,90		0,84	
YP_003577035	rplM	50S ribosomal protein L13	29,64	31,54	45,82	11,14	38,56	22,23	41,58	5,05	40,99	5,33	50,12	10,84	65,15	2,14	66,21	8,36
YP_003577036	rpsI	30S ribosomal protein S9	63,51	9,52			73,26	22,35	46,50		50,88		69,99		75,02		87,91	4,25
YP_003577041	adhC	bifunctional alcohol dehydrogenase/	10,99	4,72	8,27	14,68	12,13	8,75	11,98	8,27	8,38	17,80	9,83	21,65	6,98	11,91	6,72	9,51
YP_003577042	fghA	S-formylglutathione hydrolase	1,07		1,55		1,68		1,66		1,78		1,94		3,75			
YP_003577043	rcc00871	hypothetical protein	8,95	7,33	6,99	29,88	10,10	11,36	6,77	18,59	6,16				6,26			
YP_003577046	rcc00874	inositol monophosphatase	1,71		2,09	37,44	2,66	0,98	2,14	11,55					1,85			
YP_003577048	guaD	guanine deaminase	1,51	27,91	2,22		2,43	7,13	3,46		1,60		2,27	14,08				
YP_003577050	rcc00878	5-formyltetrahydrofolate cyclo-ligase															0,61	
YP_003577051	phnA	protein PhnA															5,91	
YP_003577053	rcc00881	metallophosphoesterase			1,55								1,52					
YP_003577056	rcc00884	hypothetical protein	31,60	21,59	25,91	21,74	29,90	36,15	19,98	2,67	32,14	18,19	22,32	24,02	27,97	23,65	23,64	37,65
YP_003577065	proV1	glycine betaine/L-proline ABC transp	1,00	18,49			1,32		1,72	79,60					0,74		1,05	
YP_003577067	proX1	glycine betaine/L-proline ABC transp	16,80	14,98	32,62	46,73	25,12	6,63	27,89	4,21	23,12	17,05	29,30	33,76	10,61	12,73	14,75	17,97
YP_003577078	pccB	propionyl-CoA carboxylase subunit b	0,92		1,22								0,80	9,40				
YP_003577081	rcc00909	hypothetical protein	1,73															
YP_003577083	pccA	propionyl-CoA carboxylase subunit a	1,69		3,17	24,92	2,86	4,38	3,87	10,17	2,67	12,91	4,27	12,46	2,83	23,65	3,23	
YP_003577084	bhbA	methylmalonyl-CoA mutase	3,65	4,81	2,85	19,25	3,32	14,38	3,99	13,70	2,57	38,70	3,44	9,40	2,37		3,81	
YP_003577085	bfr	bacterioferritin	44,56	37,41	25,87	21,21	81,87	14,21	27,51	43,75	48,85	48,31	14,21	3,01	3,04	7,06	8,44	29,75
YP_003577087	psd	phosphatidylserine decarboxylase pr	3,44	4,54	2,22	20,24	2,01	23,78	2,13	22,16	1,87		1,69		2,25		2,45	
YP_003577100	rcc00928	hypothetical protein													0,66			

YP_003577102	rcc00930	hypothetical protein									2,21							
YP_003577107	rcc00935	hypothetical protein	2,33		5,13	28,51	9,00	17,96	10,11	17,69	3,72	5,18	5,38	7,34	3,75	27,23	3,83	14,08
YP_003577124	hsdM1	type I restriction-modification system					1,42		1,31	27,83			0,76		1,41		1,73	
YP_003577126	hsdS1	type I restriction-modification system					1,07											
YP_003577133	rcc00963	hypothetical protein			1,54	1,78												
YP_003577149	rcc00979	hypothetical protein							2,54		2,73							
YP_003577166	hup2	DNA-binding protein HU									1,15							
YP_003577181	rcc01011	S24 family peptidase									1,31				0,58			
YP_003577188	ugpC	sn-glycerol-3-phosphate transport AT					1,58										7,37	
YP_003577199	cbiO1	cobalt ABC transporter ATP-binding p			0,83		1,73		1,10								0,59	
YP_003577202	rcc01035	hypothetical protein	4,41	19,32	4,80	21,83	4,54	22,13	6,56	28,60	2,60	29,52	6,94	11,86			1,62	
YP_003577205	rcc01038	hypothetical protein	14,07	14,53	14,65	19,19	15,44	15,99	24,23	14,05	9,89	27,44	20,36	15,36	9,15	15,59	12,36	28,38
YP_003577214	rcc01047	iron siderophore/cobalamin ABC trar													1,63	9,12		
YP_003577229	gvpK	gas vesicle protein GvpK															0,61	
YP_003577233	pyp	photoactive yellow protein	0,94				2,54	6,04	0,75		2,74	51,38			1,02	3,34	1,89	29,40
YP_003577248	rcc01081	group 1 glycosyl transferase	1,45		1,88		2,35		2,17		1,09		0,30					
YP_003577250	lspL1	UDP-glucuronate 5'-epimerase	1,29	13,26	1,04		1,44	23,71	1,75	22,87					1,72		1,92	
YP_003577256	gsiB	glutathione ABC transporter periplas	4,17	19,98	3,06	8,45	4,20	9,05	4,97	10,87	3,02	21,90	3,87	8,96			2,06	
YP_003577260	pepA1	leucyl aminopeptidase					0,60		0,86	18,09			0,63				0,48	
YP_003577274	rsbQ	sigma factor SigB regulation protein	4,24	40,69	3,18	8,23	3,70	13,82	4,62	14,59	3,28	19,22	2,40	26,11	1,98	8,96	1,95	31,09
YP_003577278	rcc01113	MiaB family RNA modification enzym											1,93					
YP_003577279	rcc01114	OmpA/MotB domain-containing prot			1,52													
YP_003577281	rcc01116	PhoH family protein	4,95	7,05	3,74	17,74	5,14	16,59	5,40	13,80	3,46	13,36	5,04	3,39	3,36	9,97	3,92	26,33
YP_003577283	corC	magnesium and cobalt efflux protein	0,98		2,94		1,12		1,32	16,54	0,69	13,00	0,91		1,18	18,57	0,39	
YP_003577285	metK	methionine adenosyltransferase	53,19	5,68	43,96	18,62	51,73	16,31	57,22	6,62	47,64	3,28	53,30	3,76	69,11	11,64	70,34	21,43
YP_003577287	aroA	3-phosphoshikimate 1-carboxyvinyltr	8,79	84,65	3,78	40,52	3,65	3,70	4,61	16,85	3,06	21,18	4,77	28,54	3,12	12,26	3,67	7,81
YP_003577288	cmk	cytidylate kinase													1,60			
YP_003577289	nfnB	nitroreductase/dihydropteridine red	5,87	9,13	3,66	23,88	8,15	7,46	5,11	20,34	5,18	15,47	6,39	1,86	3,11	5,72	3,15	
YP_003577290	rpsA	30S ribosomal protein S1	117,24	8,53	105,12	2,13	103,26	14,04	104,38	0,68	94,51	4,46	111,02	5,96	122,69	9,13	128,27	7,53
YP_003577291	ihfB	integration host factor subunit beta	22,39	9,21	22,71	10,99	24,81	4,18	19,64	15,52	17,24	17,82	17,83	8,09	7,38	7,01	10,26	18,69
YP_003577294	trpB1	tryptophan synthase subunit beta	4,87	10,44	4,57	28,87	6,11	11,47	7,51	8,06	4,63	21,61	6,90	7,51	5,63	2,64	6,88	4,77
YP_003577296	rcc01131	MarR family transcriptional regulator					1,83				1,55	16,57						
YP_003577297	galM	aldose 1-epimerase			1,50	1,98			1,95	11,49	1,53	39,59	1,41	16,17	1,06	2,03		
YP_003577302	pfkB	6-phosphofructokinase	4,16	4,94	4,15	19,14	5,50	13,62	5,75	19,65	3,24	10,62	20,82	103,41	4,05	10,82	4,87	20,81
YP_003577306	gltX1	glutamyl-tRNA synthetase	5,72	16,59	4,70	25,17	5,17	17,50	6,14	11,54	4,93	43,65	6,14	15,20	5,28	11,45	7,51	28,81
YP_003577307	gcvT1	glycine cleavage T protein	9,18	16,53	7,72	20,13	22,76	10,13	23,68	16,62	10,39	26,70	9,87	6,99	9,12	14,09	10,37	7,38
YP_003577309	gcvP	glycine dehydrogenase	11,96	4,41	9,88	17,84	21,61	17,62	22,50	11,03	11,94	20,51	12,40	2,59	12,94	19,42	15,44	20,83
YP_003577311	gyaR2	glyoxylate reductase	1,28	24,61	1,14	12,80	1,28	3,96	1,93	8,67	0,75		1,24		0,53		1,10	3,68

YP_003577312	csaA	chaperone CsaA	5,63	29,02	3,86	34,53	5,42	1,58	2,95	4,94	4,24	53,16	3,32	45,84	2,33	59,06	2,37	
YP_003577313	proC	pyrroline-5-carboxylate reductase	8,27	7,01	5,87	44,30	7,47	12,55	9,29	11,77	6,59	40,33	7,38	21,67	5,10	20,46	6,19	18,79
YP_003577318	hisF	imidazole glycerol phosphate synthas	2,98		3,18	12,91	3,24	10,08	3,89	10,68	3,25	11,12	4,17	0,88	2,49	4,70	3,61	
YP_003577319	hisA	1-(5-phosphoribosyl)-5-[(5-phosphor	4,30	3,87	3,73	5,98	4,06	11,59	4,47	10,78	3,84	9,62	5,10	17,13	4,05	4,22	4,93	8,65
YP_003577320	rcc01155	hypothetical protein	3,44		12,13	60,94	3,55	49,90	5,55	10,01	8,10	22,85	11,07	25,41	4,50		2,66	73,74
YP_003577323	ccoO	cbb3-type cytochrome c oxidase sub	4,49	0,17	4,87	23,40	14,15		4,71		5,57	0,61	7,96		6,19	4,77	4,37	
YP_003577325	ccoP	cbb3-type cytochrome c oxidase sub	3,79	5,60	4,05	30,23	9,07	35,60	5,04	25,84			6,15		5,34	6,24	5,97	43,50
YP_003577333	rcc01168	methyltransferase small domain-con									0,81		1,49					
YP_003577336	rcc01171	ATP-dependent RNA helicase DbpA							3,76	8,37	2,83		6,85	6,79	4,83	9,63	8,55	2,74
YP_003577337	hemE	uroporphyrinogen decarboxylase	6,49	12,99	6,58	17,04	5,66	9,13	6,92	5,60	5,85	3,11	11,07	12,05	10,80	7,99	14,96	2,45
YP_003577338	hemC	hydroxymethylbilane synthase	16,74	16,14	14,94	12,39	14,67	11,10	18,69	7,33	13,87	5,39	21,78	2,46	25,01	8,38	31,38	23,83
YP_003577341	rcc01176	GDSL-like lipase/acylhydrolase	0,77	13,08	1,57	18,75	2,04		1,27	9,18	1,94	17,23	1,27	29,18				
YP_003577344	rcc01179	transglycosylase_ Slt family			1,85								2,66	17,80			2,01	
YP_003577345	copA2	copper-transporting P-type ATPase	4,06	12,67	3,24	33,66	4,50	49,49	3,00	4,35	1,68	43,84	2,75		2,10	12,85	2,99	7,55
YP_003577347	hisH	imidazole glycerol phosphate synthas	1,79		2,25	24,90			3,23	26,51	1,58	2,46	2,53	21,67	0,95		3,12	25,23
YP_003577348	hisB	imidazoleglycerol-phosphate dehydr					0,31											
YP_003577349	rcc01184	CsbD family protein	1,34	57,42			3,14	23,28			2,33							
YP_003577351	rcc01186	hypothetical protein	1,45								1,06	11,22			0,78		1,89	8,01
YP_003577352	phnC	phosphonate ABC transporter ATP-b															0,04	
YP_003577353	phnD	phosphonate ABC transporter peripl	0,91															
YP_003577362	phnK	phosphonates transport ATP-binding															1,79	
YP_003577367	pyc	pyruvate carboxylase	6,95	10,32	5,02	13,83	12,38	16,05	12,70	6,92	6,09	17,64	6,23	1,33	5,36	20,02	6,94	26,07
YP_003577368	rcc01204	peptidoglycan binding domain-conta	5,33	24,74	6,79	58,90	3,92	19,40	4,74	24,28	4,02	12,83	7,42	56,42	3,80	9,58	4,09	8,09
YP_003577369	glyQ	glycyl-tRNA synthetase subunit alpha	0,72				3,65		3,48								2,20	
YP_003577371	glyS	glycyl-tRNA synthetase subunit beta	3,18		5,60		4,91	38,28	6,95		3,86		4,31	20,72	4,13		4,88	11,54
YP_003577372	ppdK	pyruvate_ phosphate dikinase	2,37	33,01	1,75	33,88	3,53	27,10	2,18	14,85	1,67	20,59	1,92	1,48	1,89		1,82	
YP_003577374	folB	dihydroneopterin aldolase	2,45	14,61	2,65	0,30	2,58	15,66	3,13	15,25	2,16	25,46	2,41	0,07	2,13	9,47	2,27	
YP_003577375	folP	dihydropteroate synthase	1,87		0,88		1,97		1,87		1,41		2,03				1,47	
YP_003577376	glmM	phosphoglucosamine mutase	1,97	22,63	2,50	7,27	9,93	66,15	2,85		1,46		2,22	10,34	1,01		2,30	
YP_003577378	ilvC	ketol-acid reductoisomerase	46,45	7,60	34,92	22,63	40,66	19,22	46,00	8,08	34,86	11,79	47,88	5,16	60,90	17,74	61,60	14,05
YP_003577382	ureA	urease subunit gamma	11,03				8,88	23,19	6,36		4,58							
YP_003577383	ureB	urease subunit beta					6,75	37,18										
YP_003577384	ureC	urease subunit alpha	4,35	3,99	3,12	13,51	10,27	13,98	10,08	8,24	3,92	6,97	4,18	13,84			3,22	15,76
YP_003577386	ureE	urease accessory protein UreE					2,45											
YP_003577388	ureG	urease accessory protein UreG			0,87								1,13					
YP_003577390	urtB	urea ABC transporter urea binding pr	77,28	3,01	41,39	11,11	233,07	3,89	278,03	4,74	94,58	12,81	63,55	5,51	35,04	0,17	29,52	20,43
YP_003577394	urtE	urea ABC transporter ATP-binding pr					1,08											
YP_003577395	rcc01231	UbiH/UbiF/VisC/COQ6 family ubiquir	5,51	2,20	4,25	23,78	6,15	13,45	4,48	7,11	2,95	36,15	3,63	39,24	4,15	28,68	4,48	28,86

YP_003577400	rcc01236	GntR family transcriptional regulator			2,04		1,53						2,37	21,25				
YP_003577402	carA	carbamoyl-phosphate synthase smal			5,15		3,47		5,67	6,85	4,33	18,57	5,38	14,33	4,10	40,12	5,36	20,94
YP_003577403	rcc01239	glyoxalase/bleomycin resistance prot									1,29							
YP_003577404	cycA1	cytochrome c2	154,54	11,57	387,89	15,56	175,09	20,06	358,38	5,47	409,25	7,58	321,69	9,31	294,84	7,64	202,03	1,62
YP_003577405	lipB	lipoyltransferase	1,67				1,26		3,08	27,01	1,48		2,66				2,34	
YP_003577407	potA1	polyamine ABC transporter ATP bind	3,29	11,99			9,97	24,06	9,03	9,32					5,23	5,80	6,63	19,73
YP_003577408	potD1	polyamine ABC transporter periplasn	202,76	8,34	218,71	20,82	632,56	14,29	611,37	5,16	174,66	10,91	188,84	15,30	143,66	9,00	156,98	8,40
YP_003577413	rcc01249	TatD-related deoxyribonuclease	1,89				1,69		1,72	16,66			1,65	8,03	1,47	19,63	1,26	
YP_003577416	dacC2	D-alanyl-D-alanine carboxypeptidase	2,18	15,72	2,39	41,66	2,20	27,52	2,89	15,95	1,90	34,65	2,40	26,46	2,26	6,44	2,17	18,34
YP_003577417	rcc01253	sporulation domain-containing prote	1,67														1,16	
YP_003577424	mod	type III restriction-modification syste			0,92													
YP_003577425	res	type III restriction-modification syste	0,78		1,00		1,35	15,30	1,08	13,14			1,23		1,09		1,19	
YP_003577438	rcc01276	Cas1 family CRISPR-associated protei	3,34															
YP_003577448	mcrB	McrBC restriction endonuclease syste	1,71															
YP_003577455	rcc01293	hypothetical protein									0,50							
YP_003577461	rcc01302	Csd1 family CRISPR-associated protei			1,52		1,08								0,70			
YP_003577462	rcc01303	Cas2 family CRISPR-associated protei	1,65	17,02	1,38	5,72	2,39	36,95	2,63	41,68	1,19	38,07	2,49	24,27	2,23	38,24	1,53	24,98
YP_003577472	rcc01314	hypothetical protein	2,70		1,78	14,56	1,68		2,80	25,34	2,00	26,92	2,10	8,12			1,28	
YP_003577473	rcc01315	hypothetical protein					1,66		2,68		0,78	7,01	1,07					
YP_003577483	hsdS2	type I restriction-modification system					1,64											
YP_003577491	acxA	acetone carboxylase subunit beta	10,72	15,08			7,71	15,34	4,07	11,04	6,82	30,56	5,46	16,51				
YP_003577492	acxB	acetone carboxylase subunit alpha	9,82	11,27	1,42		7,35	13,54	2,21	23,47	6,88	39,30	4,88	29,39				
YP_003577493	acxC	acetone carboxylase subunit gamma	5,34	25,79	1,01		3,61	15,71	1,33	1,27	4,09	30,08	2,82	8,58				
YP_003577499	rcc01345	hypothetical protein															0,28	
YP_003577516	leuA	2-isopropylmalate synthase	7,68	2,34	21,56	108,86	7,27	13,87	6,95	10,97	6,02	12,80	7,47	4,35	7,67	19,63	9,13	18,79
YP_003577519	nadE	NAD(+) synthase (glutamine-hydroly:			1,04	20,44	1,52				1,01		1,42	13,54	1,91			
YP_003577521	rcc01368	antibiotic biosynthesis monooxygena	16,03	3,15	12,73	6,70	16,88	12,74	13,59	8,25	11,75	16,74	11,28	17,12	5,70	13,88	8,73	20,08
YP_003577522	rcc01369	ABC transporter periplasmic substrat	60,08	76,32	44,45	32,95	104,73	42,26	54,44	44,95	142,91	48,79	55,47	17,96	9,03	17,51	21,33	16,33
YP_003577525	rcc01372	ABC transporter ATP-binding protein	2,07				2,71	40,77	1,72						1,92			
YP_003577529	dctP1	TRAP C4-dicarboxylate transport syst	13,01	7,67	10,33	25,11	24,14	13,74	29,09	5,61	10,56	12,73	11,06	5,52	3,74	26,74	4,59	32,50
YP_003577530	glnA2	glutamine synthetase			3,58		4,68	50,12	5,56	9,37	2,53		3,72	4,81				
YP_003577531	rcc01378	aldehyde dehydrogenase	10,96	14,22	8,33	17,98	14,19	15,21	15,12	6,69	9,20	15,60	11,02	4,65	7,62	20,14	8,82	
YP_003577532	rcc01379	iron-containing alcohol dehydrogena	14,86	14,53	10,95	14,89	17,90	12,90	20,24	9,68	12,28	9,75	15,59	1,69	9,80	13,50	13,50	24,69
YP_003577533	rcc01380	hypothetical protein			1,80	27,79												
YP_003577536	ugd	UDP-glucose 6-dehydrogenase	2,62	0,74	2,81	27,51	3,58	20,14	3,21	8,78	2,73	26,96	3,29	13,80	2,86	20,50	4,02	19,20
YP_003577537	uvrB	UvrABC system protein B	1,12		1,03										0,74			
YP_003577538	rcc01385	NDUFA4 family NADH ubiquinone ox	3,79	11,54	3,65	1,61	4,08	3,61	3,21		2,77	17,17	2,51	2,53	3,90		1,88	5,70
YP_003577541	potD2	polyamine ABC transporter periplasn	3,12	15,95	2,36	12,34	5,52	14,45	6,42	9,90	2,68	14,84	3,14	7,16	1,13		1,23	

YP_003577555	rcc01402	ABC transporter substrate-binding pr	19,04	11,96	23,42	11,53	20,66	11,38	23,59	5,67	23,73	8,96	29,05	13,75	16,57	8,72	19,31	4,07
YP_003577567	rcc01415	serine/threonine-protein kinase									1,95						5,14	
YP_003577575	rcc01423	hypothetical protein											3,18	2,69			3,64	
YP_003577579	rcc01427	ABC transporter ATP-binding/permea	2,67															
YP_003577593	fepC2	ferric enterobactin transport ATP-bin													0,44			
YP_003577597	rcc01445	tonB-dependent receptor	0,84															
YP_003577598	rcc01446	M20 family peptidase	9,67	30,48	6,87	18,96	7,77	8,13	9,15	7,17	7,46	21,42	10,12	6,81	7,12	11,09	8,57	13,03
YP_003577599	hemA	5-aminolevulinate synthase	22,75	14,55	13,86	34,44	28,46	20,50	31,15	14,68	21,33	27,98	12,36	21,03	29,62	0,89	42,71	34,45
YP_003577600	rcc01448	hypothetical protein	1,30		0,77						0,43		0,42		0,60		0,85	27,83
YP_003577601	ispG	4-hydroxy-3-methylbut-2-en-1-yl dip	6,66	3,15	5,37	34,90	6,18	27,75	6,30	25,26	5,15	11,40	4,67	17,45	4,09	13,47	5,66	31,57
YP_003577602	serS	seryl-tRNA synthetase	33,48	61,98	14,43	9,63	11,46	24,98	9,94	24,06	12,25	11,54	8,05	45,68	15,84	14,88	12,57	57,72
YP_003577603	rcc01451	RND family efflux transporter subuni			0,10													
YP_003577605	rcc01453	ArsR family transcriptional regulator					1,21		1,43		0,97							
YP_003577610	tatA	Sec-independent protein translocase	16,37	2,76	13,43	15,68	17,28	30,40	13,35		5,54	35,25	15,27		2,18		3,88	
YP_003577611	tatB	Sec-independent protein translocase	3,44	7,13	4,22	22,45	1,64	12,10			1,30	5,45	2,44	1,93	0,76		4,28	
YP_003577614	nrdJ2	ribonucleoside-diphosphate reducta:	2,53	0,34	2,33		2,70		3,14	22,63	2,14		2,46	11,66	2,79	13,52	4,42	
YP_003577615	rcc01463	hypothetical protein					0,73	14,86					2,42		1,16			
YP_003577617	rcc01465	hypothetical protein	5,81	3,98	5,70	22,09	6,94	16,23	7,90	5,85	4,98	14,63	7,66	4,98	4,05	6,24	6,93	24,46
YP_003577623	aspC1	aspartate aminotransferase							0,45									
YP_003577633	murE	UDP-N-acetylmuramoyl-L-alanyl-D-gl	2,97	12,97	2,06	25,24	5,89	61,18	3,32	15,98	2,33	28,97	3,23	3,31	4,70	51,53	4,34	44,29
YP_003577634	murF	UDP-N-acetylmuramoyl-tripeptide--D	2,56	13,01	1,74	22,28	2,94	26,23	2,21	18,76	1,62	9,63	2,82	30,08	2,20	23,57	2,43	23,14
YP_003577637	murD	UDP-N-acetylmuramoylalanine--D-glu	2,25	15,85	1,77	1,72	2,91		2,63	4,26	2,02	18,17	2,18	5,83	1,86	7,60	1,78	
YP_003577640	purM	phosphoribosylformylglycinamidine c	5,83	1,12	5,09	24,82	6,20	12,45	6,15	18,56	5,27	16,33	6,67	4,79	6,42	16,63	7,59	15,98
YP_003577641	purN	phosphoribosylglycinamide formyltra							2,45									
YP_003577642	rnd2	ribonuclease D	3,90		3,90	29,91	5,01	11,56	5,65	9,91	3,44	28,86	4,62	11,70	4,99	12,38	4,58	
YP_003577643	uvrD	DNA helicase II	1,08						2,36						0,83		1,17	
YP_003577645	rcc01493	hypothetical protein	16,91	22,13	16,42	9,95	23,13	5,01	19,69	8,97	16,32	7,59	21,93	13,96	14,16	11,48	24,17	56,54
YP_003577646	mdeA	methionine gamma-lyase	3,19	17,04	2,64	11,70	3,94	21,15	4,95	11,82	2,86	23,83	3,42	4,77	2,84	4,92	2,22	
YP_003577649	sufE	cysteine desulfuration protein SufE	3,38		2,17												1,45	
YP_003577651	rcc01499	DNA alkylation repair enzyme family					1,12	38,63										
YP_003577652	glmS	glutamine--fructose-6-phosphate tra	3,84	17,44	3,15	19,86	4,01	11,01	4,68	13,26	3,18	25,12	3,43	5,80	2,74	10,09	3,35	
YP_003577653	glmU	bifunctional UDP-N-acetylglucosamir	4,84	10,70	3,85	8,55	4,81	24,85	5,25	16,63	3,15	23,19	4,60	25,01	3,40	15,88	4,22	27,94
YP_003577654	gph2	phosphoglycolate phosphatase	1,60		2,20	6,11	1,45		2,68	17,54	1,87	31,13			1,57	26,13	1,94	17,90
YP_003577656	rcc01504	DegT/DnrJ/EryC1/StrS family aminot															0,57	6,70
YP_003577659	rcc01507	hypothetical protein			2,76				3,14	24,04								
YP_003577660	rcc01508	LuxR family two component transcrip															0,10	
YP_003577664	mccB	methylcrotonoyl-CoA carboxylase su					3,40											
YP_003577666	rcc01514	glutathione S-transferase									0,56							

YP_003577670	nuoB	NADH-quinone oxidoreductase subu			1,17		2,45											
YP_003577671	nuoC	NADH-quinone oxidoreductase subu	6,59	5,18	5,05	10,16	5,71	17,23	4,95	10,68	2,85	40,31	3,01	33,29	5,43	18,24	3,80	
YP_003577672	nuoD	NADH-quinone oxidoreductase subu	5,16	11,13	3,78	21,57	4,89	29,49	3,80	18,60	2,93	25,56	2,99	5,34	5,44	25,83	2,80	5,77
YP_003577673	nuoE	NADH-quinone oxidoreductase subu	14,02	11,20	8,77	4,02	11,62	12,67	8,76	4,66	7,19	5,96	7,55	11,11	9,84	5,86	6,69	20,53
YP_003577674	rcc01522	hypothetical protein	15,19	7,46	10,86	33,58	15,60	13,07	8,71	24,14	8,80		5,31	56,15	9,06		8,95	27,86
YP_003577675	nuoF	NADH-quinone oxidoreductase subu	7,95	4,45	5,07	7,39	6,36	28,27	5,02	7,65	4,55	26,67	4,54		5,78	19,87	4,01	6,89
YP_003577678	rcc01526	hypothetical protein	1,44		1,98	37,38	1,82		1,85	18,99	1,42		1,91	3,36	1,48	20,23	0,79	
YP_003577679	nuoG	NADH-quinone oxidoreductase subu	12,57	9,26	8,38	18,88	11,23	20,85	9,82	4,13	6,97	19,63	7,52	15,26	11,38	10,92	8,03	26,44
YP_003577682	rcc01530	hypothetical protein	10,52	29,04	14,63	40,16	15,50	14,42	6,94	7,68	10,23	28,21	9,52	14,15	8,52	23,50	8,26	
YP_003577683	nuoI	NADH-quinone oxidoreductase subu	2,63	44,02	3,73		3,32				2,84				1,83			
YP_003577684	pcaC	4-carboxymuconolactone decarboxyl															1,39	
YP_003577692	rcc01540	metallo-beta-lactamase	8,71	9,24	6,56	22,17	7,81	21,02	9,36	13,65	6,13	22,79	7,51	7,12	7,51	14,91	7,86	12,86
YP_003577694	ilvH	acetolactate synthase small subunit	13,05	25,78	14,69	6,75	10,46	16,00	10,15	15,41	11,22	2,05	15,89	7,79	8,91	21,64	15,75	35,50
YP_003577695	ilvI	acetolactate synthase large subunit	6,79	1,88	5,90	24,85	5,53	20,95	5,62	9,54	4,82	19,16	7,09	7,03	5,36	20,72	8,71	31,69
YP_003577698	rcc01546	AMP-dependent synthetase and ligas	10,63	2,87	10,54	12,56	7,83	13,85	12,42	9,28	7,88	25,05	11,34	3,52	3,96	20,76	6,81	2,07
YP_003577699	prfB	peptide chain release factor 2	3,20	37,99	3,86		3,55	22,09	5,67		4,04	7,71	4,60	43,71	3,08		4,60	
YP_003577701	rcc01549	hypothetical protein	51,99	8,71	42,88	9,10	48,95	5,80	39,78	0,62	35,21	8,27	37,53	12,67	25,61	5,65	32,54	16,04
YP_003577702	rcc01550	M23 family peptidase			2,04				1,02	19,50	0,62							
YP_003577703	bcp	peroxiredoxin	8,43	23,68	12,75	4,17	15,33	17,94	11,75	20,46	11,42	34,93	14,68	3,04	8,07	4,25	9,46	21,90
YP_003577705	queA	S-adenosylmethionine:tRNA ribosyltr	1,01		1,07	20,40			1,67		1,01		1,29	0,13	1,02		0,95	16,13
YP_003577707	rcc01555	hypothetical protein	1,67				2,59	11,16	2,64	20,31	1,44							
YP_003577708	lpdA2	dihydrolipoyl dehydrogenase	11,30	11,27	8,57	24,78	13,87	19,79	16,69	12,55	9,03	29,80	9,22	8,47	9,85	10,69	9,84	21,23
YP_003577710	uvrA	UvrABC system protein A			13,58				12,70				22,39		1,33		1,90	31,11
YP_003577717	mmsA	methylmalonate-semialdehyde dehy	7,25						1,87	36,15			2,40	25,03				
YP_003577721	purF	amidophosphoribosyltransferase	8,86	39,77	5,22	22,08	6,73	20,09	7,35	9,79	5,39	24,37	6,49	17,42	6,30	16,20	7,86	16,77
YP_003577722	cvpA	colicin V production protein									1,01							
YP_003577726	alr	alanine racemase	5,21	8,25	4,46	12,15	4,51	10,89	5,67	12,88	4,81	32,29	7,03	1,23	5,65	19,37	7,46	21,43
YP_003577727	dnaB	replicative DNA helicase			1,08		1,88											
YP_003577728	pyrE	orotate phosphoribosyltransferase	7,78	5,71	5,56	27,29	5,55	10,72	7,19	18,12	5,22	36,29	6,46	16,02	7,03	9,12	8,41	0,32
YP_003577730	rcc01578	lipoprotein			4,97	22,19	3,12	45,92	4,08	8,06	3,20	29,48	4,73		3,97	5,37	5,50	20,14
YP_003577736	rcc01584	hypothetical protein															1,07	
YP_003577737	rcc01585	hypothetical protein	2,47		2,32		0,89		0,64		1,02		0,64					
YP_003577738	rcc01586	GSCFA family protein	6,36	15,63	5,57	1,07	6,16	14,88	7,00	17,28	4,06	29,22	4,47	18,57	3,38	37,73	4,30	6,32
YP_003577739	rcc01587	hypothetical protein			0,58													
YP_003577740	rcc01588	GSCFA family protein			0,77		1,02	44,05	1,04	14,49	1,33	4,15	1,69	47,04	0,87	36,02	1,65	
YP_003577742	infC	translation initiation factor IF			8,72	48,93	15,86	20,43			7,85		11,22	1,60	10,15		14,65	
YP_003577743	fpr	ferredoxin--NADP reductase	16,69	13,47	16,11	20,19	15,12	15,42	19,70	7,59	18,96	9,22	17,87	6,61	18,52	7,40	18,55	13,74
YP_003577744	rcc01592	hypothetical protein	6,07	6,53	4,44	16,61	4,57	29,56	3,13	20,23	5,14	7,90	2,91	10,98	4,74	2,98	3,64	2,94

YP_003577746	cysI	sulfite reductase (NADPH) hemoprot	10,60	10,48	7,35	18,79	8,60	17,00	8,67	10,81	7,29	22,57	9,21	7,70	10,56	18,12	10,36	25,23
YP_003577747	rcc01595	hypothetical protein	9,28		5,70	22,30	3,20	11,11	6,74						5,80	20,99		
YP_003577750	tpiA	triose-phosphate isomerase	40,69	8,24	38,96	14,18	58,83	8,70	61,75	3,06	42,99	12,45	32,19	12,60	28,04	5,25	40,04	10,89
YP_003577752	nemA	N-ethylmaleimide reductase	8,42	13,60	6,84	13,70	9,66	16,24	8,65	12,37	7,80	22,46	8,62	6,68	8,78	12,27	9,15	25,93
YP_003577757	rcc01605	thiamine monophosphate synthase	4,36	16,13			3,12								1,90		2,59	
YP_003577758	rcc01606	tRNA/rRNA methyltransferase_ SpoU					0,62											
YP_003577760	rcc01608	cytochrome c oxidase assembly prote					0,59											
YP_003577761	cxp	thermostable carboxypeptidase 1	4,87	5,49	4,91	4,60	5,46	14,92	5,27	32,52	3,81	34,62	4,56	14,41	4,17	11,45	4,84	4,96
YP_003577764	rcc01612	hypothetical protein					1,54											
YP_003577766	metH4	methionine synthase subunit B	4,70	12,54	3,95	33,19	5,57	27,89	5,34	9,18	3,93	49,99	5,14	25,19	4,12	9,64	6,89	14,72
YP_003577767	rcc01615	methylenetetrahydrofolate reductase															1,11	
YP_003577772	aldB	aldehyde dehydrogenase	46,95	10,72	28,66	11,53	28,01	17,19	17,12	1,81	39,23	19,82	53,76	14,81	11,07	8,70	18,46	27,39
YP_003577777	pyrH	uridylate kinase	10,45	10,27	9,66	17,23	13,40	11,08	16,29	7,85	10,63	14,61	11,88	1,29	8,37	27,39	12,48	18,59
YP_003577778	frr	ribosome recycling factor	28,79	24,32	24,79	21,08	31,67	29,35	17,14	2,58	27,74	1,07	16,74	25,40	18,51	8,46	18,22	34,59
YP_003577779	uppS	di-trans_poly-cis-decaprenylcistransf			0,58													
YP_003577781	dxr	1-deoxy-D-xylulose-5-phosphate redu			1,20		1,33		2,15	15,86	2,01		2,20	29,87			1,13	
YP_003577782	rcc01630	M50 family peptidase			1,62		1,55				1,13		1,42				0,94	
YP_003577784	yaeT	outer membrane protein assembly fa	4,41	32,81	3,88	42,49	2,91	33,26	2,95	12,11			3,52		2,26		3,75	21,77
YP_003577785	rcc01633	outer membrane chaperone Skp fam	3,20	26,34	2,72		3,17	28,11			2,40		1,29					
YP_003577786	fabZ	(3R)-hydroxymyristoyl-ACP dehydrat											2,39				1,92	
YP_003577787	lpxA	acyl-ACP--UDP-N-acetylglucosamine			2,05				2,60		1,61		1,81				1,71	
YP_003577793	rcc01641	transglutaminase-like family protein							0,90									
YP_003577794	rcc01642	hypothetical protein					0,90		1,83									
YP_003577795	rcc01643	transglutaminase-like family protein	4,59	5,64	3,70		4,77	38,20	3,41	10,17	2,96	47,16	2,71	4,55	3,30		4,80	
YP_003577813	trmU	tRNA (5-methylaminomethyl-2-thiou			1,95				1,61	24,42					1,62			
YP_003577815	ctrA	cell cycle transcriptional regulator Ct	3,14	10,02	6,19	63,89	5,06	2,54	5,89	14,97	2,87	9,68	3,28	32,98			2,17	
YP_003577819	rcc01667	methyl-accepting chemotaxis sensory					0,10											
YP_003577820	rcc01668	hypothetical protein													1,27			
YP_003577823	rcc01671	carbohydrate kinase							1,19				0,49					
YP_003577825	glnB1	nitrogen regulatory protein P-II	123,72	12,35	111,30	34,95	110,41	18,99	77,39	10,29	101,31	18,07	68,51	10,76	73,71	19,24	56,91	17,79
YP_003577826	glnA3	glutamine synthetase	531,73	10,17	453,59	17,59	612,58	16,25	778,94	3,10	479,00	17,59	483,22	13,28	447,40	3,57	397,87	13,01
YP_003577827	fabD	ACP S-malonyltransferase	12,12	17,76	8,80	29,86	10,90	8,34	12,45	7,57	10,42	18,38	11,14	19,68	11,19	2,60	13,74	19,15
YP_003577828	fabG	3-oxoacyl-ACP reductase	15,13	12,93	11,65	17,64	12,94	14,57	14,44	6,88	11,23	11,89	14,69	1,29	12,55	15,67	18,35	24,27
YP_003577832	fabF1	3-oxoacyl-ACP synthase II	8,27	20,89	7,57	24,27	9,17	32,22	8,79	16,51	6,70	34,38	7,40	12,95	6,45	11,63	12,09	52,22
YP_003577833	rcc01681	aminodeoxychorismate lyase			1,53								1,54					
YP_003577836	rcc01684	HK97 family phage portal protein															0,13	
YP_003577839	rcc01687	HK97 family phage major capsid prot			6,56		6,14	22,80	7,38	6,63	4,42							
YP_003577852	cysE1	serine O-acetyltransferase							2,04				2,42					

YP_003577855	pdhC	pyruvate dehydrogenase complex E2	9,68	1,14	6,52	18,65	6,08	13,21	6,51	7,57	6,96	18,82	8,40	10,39	13,78	11,55	8,75	19,44
YP_003577856	pdhB	pyruvate dehydrogenase complex E1	15,66	14,69	10,18	20,16	9,87	14,24	10,83	7,19	11,37	18,87	12,55	6,03	24,21	18,08	14,50	21,48
YP_003577857	pdhA	pyruvate dehydrogenase complex E1	9,88	3,11	6,66	6,79	6,25	16,75	6,06	15,13	7,54	20,96	8,55	1,40	15,48	15,06	9,57	15,94
YP_003577859	fda	fructose-bisphosphate aldolase	42,26	6,56	31,50	23,14	39,87	10,42	45,96	5,00	35,26	17,89	33,15	14,28	36,32	9,66	39,76	0,82
YP_003577860	pgk	phosphoglycerate kinase	74,42	7,02	66,80	12,92	89,02	9,65	92,94	5,32	80,57	6,14	77,95	5,32	51,55	6,44	68,04	2,09
YP_003577861	ppiA	peptidyl-prolyl cis-trans isomerase A	14,87	16,00	24,20	14,25	15,81	22,98	23,05	8,32	15,76	41,86	21,41	20,51	16,81	6,39	22,72	14,76
YP_003577862	ppiB	peptidyl-prolyl cis-trans isomerase B	2,94		4,28						3,75		3,21				3,62	
YP_003577863	tyrS	tyrosyl-tRNA synthetase	10,65	5,16	7,51	36,08	7,37	26,67	7,87	13,39	6,53	35,50	5,67	17,79	7,65	3,84	9,65	16,75
YP_003577864	anmK	anhydro-N-acetylmuramic acid kinas	0,81				0,60		1,54				1,16					
YP_003577866	rcc01714	hypothetical protein			3,57		3,01		1,82	9,32	1,58	4,04	2,34					
YP_003577867	eno	phosphopyruvate hydratase	28,50	4,98	24,58	16,20	31,38	15,38	38,77	3,09	25,06	15,75	32,50	5,46	25,00	9,41	32,24	22,71
YP_003577869	rcc01717	hypothetical protein															1,98	
YP_003577870	rihA	pyrimidine-specific ribonucleoside hy							2,07								1,58	
YP_003577872	ndk	nucleoside diphosphate kinase	55,33	9,57	46,69	35,45	54,09	11,38	33,59	5,15	45,36	27,60	48,80	22,44	36,32	3,94	49,31	1,87
YP_003577875	ccpA	cytochrome-c peroxidase	1,63		0,64		1,66	44,15	0,80		1,54	20,82	0,60	12,58				
YP_003577876	speB1	agmatinase	4,29	13,26	3,85	39,83	4,56	9,82	6,39	18,05	2,41		4,37		2,93		7,30	
YP_003577878	mcpH	methyl-accepting chemotaxis protein					1,75											
YP_003577879	pyrD1	dihydroorotate oxidase			0,59				0,62									
YP_003577882	rcc01730	U32 family peptidase	2,35		2,02	0,04	1,59	18,21	2,34	13,40	1,61	32,01	1,65	11,04	2,35	11,86	2,99	18,65
YP_003577883	rcc01731	U32 family peptidase	2,19		4,23		2,75	19,17	3,59	19,83	2,46	5,01	3,48	3,13	3,91		5,55	20,30
YP_003577890	katG	catalase/peroxidase	6,00	5,97	9,12	21,09	5,27	15,65	10,61	10,80	4,46	20,61	14,22	25,99	6,55	17,75	7,97	26,37
YP_003577895	rcc01743	DSBA family oxidoreductase	10,04	23,06	9,29	12,45	9,86	25,68	7,47	12,20	9,66	4,95	9,05	20,51	7,64	3,04	6,48	35,07
YP_003577896	rcc01744	class I aminotransferase			1,01		1,70		1,98	43,17	1,11		1,96	42,07	0,72		2,36	
YP_003577897	amiC	N-acetylmuramoyl-L-alanine amidase															1,80	
YP_003577900	rsmB2	ribosomal RNA small subunit methylt					2,88										0,92	
YP_003577903	recA	RecA protein	3,41	6,51	3,11	31,33	3,53	18,44	4,28	10,07	3,33	21,01	4,35	5,26	5,54	7,29	5,92	25,03
YP_003577904	alaS	alanyl-tRNA synthetase	12,99	45,39	7,84	24,64	10,67	27,90	11,82	12,39	8,33	24,62	8,56	4,27	10,24	28,41	13,14	37,48
YP_003577905	rcc01753	hypothetical protein	7,66	12,08	4,75	18,38	6,07	3,69	3,75	31,70	5,52	13,01	5,28	24,14	3,28	6,67	5,85	
YP_003577907	cysS	cysteinyl-tRNA synthetase					2,14	0,86	2,79									
YP_003577908	rcc01756	2-isopropylmalate synthase/homocit	2,18	0,36	3,61	9,10	2,33	17,84	2,47	30,76	1,87	19,55	2,44	13,55	4,38		2,10	1,91
YP_003577918	cheY3	chemotaxis protein CheY					1,57										2,22	
YP_003577921	bglA	beta-glucosidase A					0,38		0,56	16,77							1,03	
YP_003577923	aglE	alpha-glucoside ABC transporter sub	16,16	24,57	13,38	11,92	9,38	6,83	13,05	15,10	10,78	17,92	12,45	22,02	7,57	2,23	7,89	9,72
YP_003577926	aglA	alpha-glucosidase			0,97		1,12		26,57	64,12	2,08	9,56	1,16	18,22	0,40			
YP_003577928	rcc01776	trimethylamine methyltransferase	9,07		27,10	10,22	0,63				4,47		23,47	4,00			18,82	
YP_003577931	rcc01779	MazG family protein			1,49		1,96						2,00				0,73	
YP_003577932	rcc01780	M20 family peptidase	2,10	26,14	2,78	4,96	2,62	11,70	2,79	16,95	1,33	46,28	3,25	10,59	2,03	4,03	2,25	16,92
YP_003577933	yajC	preprotein translocase subunit YajC	16,35		13,24				7,31				12,64		5,51	1,44	9,58	

YP_003577934	secD	protein-export membrane protein Se	7,56	24,35	6,60	40,79	4,45	34,40	3,37	14,55	3,31	25,61			3,23		6,90	5,73
YP_003577935	secF	protein-export membrane protein Se															0,67	
YP_003577936	rcc01784	hypothetical protein	1,36		1,21		1,07				1,01		1,21	3,44	1,39	36,51	1,77	
YP_003577941	ccmG	thiol:disulfide interchange protein	2,31	11,64	2,03		1,87	5,36					1,66				1,53	
YP_003577942	hpt	hypoxanthine phosphoribosyltransfe			1,06				0,58									
YP_003577947	ispDF	bifunctional 2-C-methyl-D-erythritol	8,37		6,01	17,95	8,44	4,75	7,41		5,95		6,19				7,65	
YP_003577949	ntrB	nitrogen regulation protein NtrB					1,44	16,45	1,07	5,03	1,12							
YP_003577950	ntrC	nitrogen assimilation regulatory prot	3,43		2,68	10,26	4,49	31,39	4,46	16,79	2,45	30,01	2,13	12,98	1,84		1,90	
YP_003577952	ntrX	nitrogen assimilation regulatory prot	2,46	28,68	1,56	41,94	2,07	3,72	2,87	13,27	1,57	51,38	1,98	11,68	1,49	15,38	2,37	10,19
YP_003577956	garR	2-hydroxy-3-oxopropionate reductas	1,62	15,23	1,82	13,24	1,48		2,37		1,54		2,36				1,88	
YP_003577957	ssb1	single-stranded DNA-binding protein	3,95	19,20	4,16	22,00	3,69	9,19	5,35	7,84	3,19	23,63	4,87	19,06	4,01	13,30	3,68	25,65
YP_003577959	rcc01807	hypothetical protein															2,68	
YP_003577960	hemB	porphobilinogen synthase	9,07	12,85	6,76	19,33	7,96	9,86	8,67	12,19	6,84	18,43	7,28	8,01	6,71	7,63	8,11	9,28
YP_003577964	rcc01812	DSBA family oxidoreductase			0,96		1,17						1,28				1,14	
YP_003577965	rcc01813	AMP-dependent synthetase and ligas	0,88										8,03					
YP_003577968	rcc01816	family 5 extracellular solute-binding	1,68	17,92	1,37	34,33	1,78	21,02	2,01	19,68	1,65	40,88	1,95	31,47	1,79	10,09	1,84	12,16
YP_003577969	bdhA	3-hydroxybutyrate dehydrogenase	10,02	23,60	9,64	10,19	11,74	14,13	14,85	8,27	9,83	15,77	10,89	12,80	5,86	16,36	7,97	26,76
YP_003577971	rcc01819	indigoidine synthase A like protein fa	5,64	0,65	5,38	23,69	5,97	10,01	6,77	21,51	6,36	41,51	7,31	13,21	6,96	13,85	9,84	20,01
YP_003577972	rcc01820	carbohydrate/purine kinase															1,58	1,05
YP_003577973	rpsB	30S ribosomal protein S2	54,37	17,24	59,52	15,31	54,60	15,21	71,33	3,90	57,70	14,76	78,02	11,28	80,65	8,63	99,70	20,80
YP_003577974	tsf	translation elongation factor Ts	123,89	3,51	107,54	12,32	122,38	9,88	94,48	0,64	108,73	10,34	97,78	7,38	102,54	0,70	120,24	0,70
YP_003577976	rcc01824	hypothetical protein					11,98										5,74	
YP_003577979	rcc01827	AhpC/TSA family protein					2,83								2,14		0,47	
YP_003577980	rpe1	ribulose-phosphate 3-epimerase	6,98	16,44	5,84	2,78	8,62	8,21	9,53	8,00	6,28	8,07	9,81	16,31	5,03	1,47	7,29	16,20
YP_003577981	cbbM	ribulose bisphosphate carboxylase la	31,04	10,16	24,25	26,96	39,02	26,81	44,62	11,38	24,66	32,96	27,68	11,98	25,09	20,71	34,05	28,69
YP_003577982	fba	fructose-bisphosphate aldolase	11,70	8,73	9,48	19,53	13,75	25,84	16,04	8,36	8,78	10,34	12,28	5,74	6,70	17,19	9,91	23,50
YP_003577983	gap1	glyceraldehyde-3-phosphate dehydr	13,62	17,17	9,50	13,59	14,04	17,52	15,85	7,94	10,70	16,90	11,72	6,30	7,18	3,47	9,30	7,50
YP_003577984	tkt1	transketolase	4,24		3,49	18,15	3,68	30,08	4,11	20,43	2,58	28,45	4,17	5,20	3,35	24,52	5,87	27,16
YP_003577985	cbbP	phosphoribulokinase	8,74	19,98	6,73	15,10	9,76	20,10	10,92	9,44	5,98	14,62	8,49	3,60	6,53	15,60	9,99	30,08
YP_003577986	fbp	fructose-bisphosphatase	4,98	4,85	4,24	18,38	6,42	16,87	7,25	8,18	3,54	26,54	5,66	4,37	3,73	27,29	4,77	11,34
YP_003577988	qor	NADPH:quinone reductase	8,12	12,67	7,64	16,90	9,73	13,53	11,45	13,13	7,34	20,21	8,33	1,93	5,50	12,61	6,65	16,43
YP_003577989	pgm	phosphoglucomutase	10,96	12,46	8,97	43,42	11,45	26,02	11,83	16,62	7,95	41,50	6,67	13,98	9,55	15,96	10,27	23,03
YP_003577991	glgA	glycogen synthase	3,97	9,95	2,59	4,15	4,07	15,31	4,13	16,43	2,54	31,04	2,63	6,40	2,21	25,94	3,00	18,03
YP_003577992	glgC	glucose-1-phosphate adenylyltransfe	5,27	19,04	3,91	21,01	7,17	13,44	7,27	9,76	4,45	19,84	5,42	8,02	3,80	0,44	5,07	26,77
YP_003577993	glgB	1_4-alpha-glucan branching protein			2,75		3,18	25,21	3,62	3,03	2,28		2,51	19,97				
YP_003577994	glgP	phosphorylase	6,08	4,29	4,14	20,58	6,86	21,67	7,15	12,90	3,91	18,86	4,50	6,54	4,06	5,75	5,40	27,16
YP_003577995	rcc01843	family 13 glycosyl hydrolase			2,20	4,60			2,29	16,05	1,60	26,67	2,49	6,26	2,10	21,36	2,52	17,98
YP_003577996	rcc01844	sensor histidine kinase	0,78		2,71		2,18		1,36	12,73	0,91		0,43		0,40		1,89	

YP_003577997	rcc01845	hypothetical protein	2,03		1,85	3,17	2,74	15,82	2,41	15,98	1,84	26,89	1,22	5,51	1,36		2,19	
YP_003578000	yicC	protein YicC									0,56				1,09		1,79	
YP_003578002	aroF	3-deoxy-7-phosphoheptulonate synt	15,59	4,18	10,60	23,25	10,75	5,19	13,45	16,00	10,26	24,48	13,01	6,51	16,25	16,92	16,93	15,34
YP_003578004	livK1	branched-chain amino acid ABC trans	126,88	21,89	167,09	33,19	171,74	37,07	169,60	10,81	171,01	17,84	137,29	17,50	107,25	21,56	107,82	46,31
YP_003578005	livG1	branched-chain amino acid ABC trans					1,35											
YP_003578010	guaA1	GMP synthase	8,90	3,37	6,86	27,97	7,67	9,73	8,69	24,67	6,08	21,32	8,79	6,66	10,33	20,54	12,16	24,21
YP_003578011	aroB	3-dehydroquinate synthase	2,67	10,51	2,42	11,77	2,42	26,18	3,18	15,81	2,35	18,42	3,00	23,00	2,04	18,67	2,62	
YP_003578016	lipA	lipoyl synthase	1,82		3,51				2,86	18,49			6,47		4,09			
YP_003578021	rcc01869	invasion associated locus B family pr	54,35	17,35	63,20	34,11	48,08	34,27	37,93	12,04	62,89	23,60	59,03	14,36	43,03	22,06	43,46	43,81
YP_003578024	lpxD	UDP-3-O-[3-hydroxymyristoyl] glucos	3,66	12,43	3,67	15,68	4,01	8,06	6,25	13,68	3,44	27,58	4,69	8,58	4,19		5,10	23,20
YP_003578027	sufS1	cysteine desulfurase	4,34		4,32		3,30		3,41	14,37	4,25	17,14	2,86	4,35			3,11	
YP_003578030	sufD	FeS assembly protein SufD	11,66	9,83	6,76	24,85	4,85	25,26	5,85	25,92	5,92	33,46	6,86	20,77	14,28	7,80	9,75	27,77
YP_003578031	sufC	FeS assembly ATPase SufC	17,59	11,58	11,38	17,89	11,57	21,44	11,53	13,53	14,43	18,18	16,70	13,62	24,58	6,81	22,45	26,50
YP_003578033	sufB	FeS assembly protein SufB	6,32		2,99		3,12		2,77		3,98	3,72	5,25	6,79	7,61	9,50	3,51	
YP_003578036	rcc01884	alpha/beta fold family hydrolase	8,34		5,45	3,20	7,57	31,67	7,87	1,22	6,87	10,60	6,71	13,13	7,10		7,47	16,95
YP_003578039	icd	isocitrate dehydrogenase	33,87	18,21	24,76	21,25	28,27	21,64	35,49	5,72	30,30	17,91	30,35	6,68	74,34	15,48	30,16	34,26
YP_003578044	typA	GTP-binding protein TypA/BipA	8,11	16,51	6,38	18,99	7,26	11,20	8,39	15,63	5,81	31,68	11,09	2,48	9,22	14,68	11,96	6,51
YP_003578045	potG1	polyamine ABC transporter ATP-bind							0,34						3,80	18,15	4,34	
YP_003578061	rpmF	50S ribosomal protein L32											6,43					
YP_003578063	fabH2	3-oxoacyl-ACP synthase III	4,75				3,10		3,76	37,92	5,59	43,24	4,26	6,50	3,81	20,41	4,46	
YP_003578064	ihfA	integration host factor subunit alpha	26,90	10,34	24,53	6,10	24,53	5,94	24,31	3,53	21,55	2,00	18,82	2,65	13,24	7,37	17,10	10,92
YP_003578067	guaB	inosine-5'-monophosphate dehydrog	11,43	6,52	9,48	23,09	9,40	13,95	11,77	17,13	8,35	29,13	8,95	11,97	10,24	20,54	11,33	19,59
YP_003578073	rcc01921	hypothetical protein					2,03											
YP_003578076	rcc01925	hypothetical protein											1,12					
YP_003578078	rcc01927	hypothetical protein													1,97			
YP_003578101	rcc01950	polysaccharide biosynthesis/export f													0,40			
YP_003578103	lspL2	UDP-glucuronate 5'-epimerase			1,02		1,08		1,14	23,77	0,76		0,90		1,11			
YP_003578106	rcc01955	hypothetical protein			14,37				6,17	27,60	8,85				3,50		4,28	
YP_003578109	wzc	tyrosine-protein kinase Wzc	1,80												0,44			
YP_003578116	gyrA	DNA gyrase subunit A			3,98	5,58	4,72	5,64	5,78	15,79	4,18	20,23	5,73	4,80	4,99	6,81	5,49	
YP_003578126	rcc01975	hypothetical protein							1,63									
YP_003578159	tig	trigger factor	198,39	8,53	166,16	11,13	156,67	10,83	106,35	7,15	141,71	7,16	140,06	9,47	186,53	13,78	190,93	31,55
YP_003578160	rplI	50S ribosomal protein L9	125,84	9,62	112,06	4,38	89,11	11,72	74,77	7,83	96,13	19,59	100,54	6,70	103,49	7,83	106,56	8,27
YP_003578161	rpsR	30S ribosomal protein S18	24,79	13,01	36,38	6,82	26,60	21,06	34,84	2,95	35,28	12,10	46,01	6,68	44,31	0,41	45,75	11,57
YP_003578162	rpsF	30S ribosomal protein S6	67,62	7,57	96,38	4,27	65,45	6,26	87,72	6,54	83,68	6,20	93,59	11,98	94,99	6,21	100,39	12,15
YP_003578165	rcc02014	hypothetical protein	7,55		3,56		5,28	45,55	1,24		4,90							
YP_003578170	rbsB	ribose ABC transporter periplasmic ri	15,43	9,25	24,53	30,61	10,50	32,23	10,46	8,85	16,76	9,88	18,74	13,00	8,73	6,29	10,16	34,10
YP_003578171	rcc02020	HAD superfamily hydrolase			0,35		0,72						0,25				1,02	

YP_003578173	rcc02022	polyols ABC transporter periplasmic	7,01	1,77	8,29	3,42	5,40	6,97	6,40	13,23	5,77	7,21	8,64	11,85	3,83	7,95	4,96	14,84
YP_003578176	rcc02025	polyols ABC transporter ATP-binding															0,61	
YP_003578181	allA	ureidoglycolate hydrolase					0,85											
YP_003578183	cobB	cobyrinic acid A_C-diamide synthase			0,63		0,34											
YP_003578189	cobF	precorrin-6A synthase											0,67				1,51	
YP_003578190	cobA2	uroporphyrinogen-III C-methyltransfe															2,31	
YP_003578191	cobM	precorrin-4 C11-methyltransferase									0,56							
YP_003578195	cobJ	precorrin-3B C17-methyltransferase					1,19						1,24					
YP_003578197	cobH	precorrin-8X methylmutase	3,60	3,65	2,52	21,55	2,96	19,02	3,00	4,77	2,09	12,61	3,33	4,41	3,20	17,08	3,86	14,85
YP_003578198	cobN	cobaltochelatase subunit CobN															1,37	
YP_003578199	cobW	cobalamin biosynthesis protein CobV	6,30	1,51	9,89	8,09	8,00	23,50	9,27	15,44	8,23	1,53	10,19	6,01	8,72	8,37	12,24	19,82
YP_003578201	cobO	cob(I)yrinic acid a_c-diamide adenos					0,76				0,89						0,91	
YP_003578203	bluB	cob(II)yrinic acid a_c-diamide reducta	2,26		1,78	17,01	2,44				2,65		3,47	29,14	2,35		2,58	
YP_003578204	cobC	threonine-phosphate decarboxylase	1,55	2,76	1,31	11,21	3,05	88,50	1,22	9,76	1,57		1,57	17,59	0,97	4,23	1,03	
YP_003578209	cobU	nicotinate-nucleotide--dimethylbenz	7,96	3,74	6,57	24,09	6,88	10,51	7,82	20,05	6,39	28,80	8,13	2,14	7,10	13,99	10,74	29,46
YP_003578212	rcc02061	GNAT family acetyltransferase	0,89		0,91								0,47					
YP_003578222	gap2	glyceraldehyde-3-phosphate dehydro	0,34		0,37													
YP_003578224	rcc02074	ferredoxin domain-containing protei					0,69											
YP_003578230	rcc02080	HAD superfamily hydrolase	3,42	18,94	3,51	12,17	3,14		2,76	17,25	2,65	63,62	3,12		3,46	23,16	4,46	
YP_003578232	ribF	riboflavin biosynthesis protein RibF					2,59											
YP_003578234	ltaE	threonine aldolase	1,90		1,54		2,07	30,01	1,72		1,07		2,16	1,19	2,48		3,46	
YP_003578243	rcc02106	NACHT domain-containing protein					0,46										3,74	
YP_003578246	rcc02109	hypothetical protein	2,00		2,87	25,68	1,27	1,43	0,90		2,69	18,01	2,22		1,45	21,67	1,43	2,59
YP_003578247	rcc02110	multicopper oxidase	25,39	9,16	27,07	4,27	30,14	14,71	18,64	14,93	27,45	14,39	40,83	19,34	15,40	17,04	22,49	45,38
YP_003578255	cat	chloramphenicol acetyltransferase					3,49		2,96									
YP_003578256	rcc02119	type 12 family methyltransferase					0,79						1,70					
YP_003578257	rcc02120	ABC transporter ATP-binding/peripla													0,33		1,54	
YP_003578261	actP2	cation/acetate symporter ActP			6,22				2,24				4,29					
YP_003578263	acsA1	acetate--CoA ligase	21,59	4,43	28,30	27,10	21,03	20,42	30,45	9,02	23,72	22,21	30,93	12,26	21,63	13,72	29,65	23,59
YP_003578271	sbcC	exonuclease SbcCD subunit C					2,35											
YP_003578276	rcc02139	methyl-accepting chemotaxis protein			1,73				0,20		2,90		0,15		0,57			
YP_003578281	rcc02144	ZapA family cell division protein	7,88	31,99	4,66	13,99	6,92	38,02			5,82	48,17	6,71		1,03		2,03	
YP_003578282	rcc02145	hypothetical protein	7,61	69,33	3,14	41,33	8,06	32,61	1,69	35,61	9,20	7,33	1,08				2,22	
YP_003578283	tkt2	transketolase	22,61	11,08	17,29	18,49	21,51	18,48	23,92	5,55	17,52	20,30	19,08	3,54	18,40	13,33	24,01	28,27
YP_003578286	rcc02149	hypothetical protein	1,21		2,36		1,08	46,01	2,27		2,04		2,22		1,26	35,83	1,58	50,49
YP_003578287	acnA	aconitate hydratase	22,70	9,70	17,13	20,42	20,42	16,34	24,77	9,31	20,99	19,27	19,81	8,64	50,92	17,42	21,52	33,54
YP_003578288	rcc02151	methyl-accepting chemotaxis sensory					0,44											
YP_003578294	purB	adenylosuccinate lyase	9,49	15,09	7,32	20,38	9,08	12,56	10,42	10,10	7,20	25,92	9,96	5,38	8,23	15,60	10,55	20,10

YP_003578295	fliG	flagellar motor switch protein FliG															0,96	
YP_003578297	gap3	glyceraldehyde-3-phosphate dehydro	59,95	7,57	47,73	16,17	61,90	15,95	74,85	4,30	57,16	11,36	59,82	10,39	57,89	8,26	68,35	17,83
YP_003578302	rne	ribonuclease E	14,53	9,61	14,08	10,57	13,20	5,56	16,23	4,92	10,59	15,85	13,88	12,92	11,27	0,23	13,69	13,82
YP_003578303	dctD	C4-dicarboxylate transport transcript	6,78	17,62	8,30	19,86	7,68	16,66	11,23	10,81	6,69	22,16	11,06	4,24	7,30	11,29	10,62	28,77
YP_003578305	purQ	phosphoribosylformylglycinamidine s	10,59	9,28	9,77	25,13	11,77	24,24	13,89	12,22	9,89	22,16	11,11	6,63	10,41	14,14	12,43	22,89
YP_003578306	purS	phosphoribosylformylglycinamidine s	17,58	18,84	19,56	44,72	23,08	46,31	12,53	15,64	21,51	14,19	14,13	3,20	9,79	16,23	15,32	33,85
YP_003578307	purC	phosphoribosylaminoimidazole-succi	8,90	15,62	9,21	21,90	10,49	19,55	12,62	10,03	8,56	25,85	11,42	15,70	10,60	11,75	13,28	17,23
YP_003578308	rcc02171	hypothetical protein	3,20				5,53	25,76			5,62	75,54						
YP_003578310	metH3	methionine synthase subunit A	1,94	14,75	2,49	16,96	2,05	40,51	3,55	6,89	1,64	12,42	2,30	0,44	1,54	12,49	2,27	
YP_003578319	rcc02182	class III aminotransferase	8,73	5,13	6,02	26,62	10,39	19,38	9,80	20,08	6,84	30,19	7,53	17,35	9,96	20,69	12,15	27,35
YP_003578320	potG2	polyamine ABC transporter ATP-bind	9,27				11,19	17,50	6,15	59,29					12,36	2,25	13,69	
YP_003578323	potF	polyamine ABC transporter periplasm	23,69	8,78	19,52	5,47	41,21	2,51	36,21	10,91	22,85	15,86	19,35	4,12	11,11	8,64	8,55	4,58
YP_003578324	rcc02187	amidohydrolase	3,61		2,57		5,30	5,81	4,36	17,49	3,68	7,98	3,10	4,33				
YP_003578327	zntA2	heavy metal translocating P-type ATP					5,74											
YP_003578328	rcc02191	MerR family transcriptional regulator															0,62	
YP_003578331	rcc02194	phospholipase/carboxylesterase	6,24	0,48	4,99	7,58	4,53	22,01	4,13	26,44	5,99		4,43		6,34	17,92	6,71	23,71
YP_003578352	rcc02215	sulfotransferase							0,64									
YP_003578356	modA2	molybdate ABC transporter periplasm	19,49	9,88							40,49	16,98			45,93	13,38	27,62	15,56
YP_003578357	modC2	molybdate ABC transporter ATP-bind	1,60															
YP_003578358	guaA2	GMP synthase									4,83	15,76			7,29	14,24	5,17	42,14
YP_003578361	rcc02224	ABC transporter substrate-binding pr					0,22										0,24	
YP_003578362	rcc02225	ABC transporter ATP-binding protein							0,47									
YP_003578377	tpa	taurine--pyruvate aminotransferase															1,36	
YP_003578385	rcc02248	aldo/keto reductase family oxidoredu	4,71	9,55	2,91	8,22	7,80	13,18	3,35	42,09	4,59	25,55	2,45					
YP_003578398	rcc02261	hypothetical protein											26,15					
YP_003578402	rcc02265	short-chain dehydrogenase/reductas													1,72		2,39	47,74
YP_003578405	potD3	polyamine ABC transporter periplasm	5,67	24,65	3,80	16,81	13,52	10,43	12,46	5,41	5,73	4,20	9,66	4,28	15,73	7,19	23,27	18,24
YP_003578406	potA3	polyamine ABC transporter ATP-bind															1,58	6,87
YP_003578412	oppA2	oligopeptide ABC transporter peripla	3,16	1,28	2,41	34,21	4,11	9,23	2,34	14,56	2,17	8,67						
YP_003578424	yieF	NAD(P)H dehydrogenase (quinone)	2,58		1,64				4,89				1,96					
YP_003578426	rcc02289	two-component response regulator r	1,87	14,29	1,78	17,54	2,52	1,85	1,41		1,62		1,45	23,05			1,54	
YP_003578432	proB	glutamate 5-kinase	1,36				1,04		1,62	34,58			1,08	20,16	1,30		1,43	11,83
YP_003578433	rcc02296	GTP-binding protein Obg/CgtA	2,25	2,44	1,99	29,67	2,73	15,29	2,49	14,11	2,24	28,07	2,68	14,40	2,30	0,94	2,49	0,08
YP_003578435	rcc02298	GNAT family acetyltransferase			7,67	9,76			7,27	29,32	8,03	7,84	10,04	3,27	7,72	12,11	11,57	11,55
YP_003578436	rpmA	50S ribosomal protein L27	24,69	11,80	54,96	9,16	37,14	34,05	47,47	4,68	55,87	13,35	55,91	11,01	88,19	14,22	82,77	14,94
YP_003578437	rplU	50S ribosomal protein L21	52,11	12,90	61,37	21,04	48,91	19,31	35,69	9,83	50,91	27,36	51,73	12,53	59,65	23,85	54,69	23,85
YP_003578439	engA	GTP-binding protein EngA	2,87		2,09		2,23	16,31	3,02	20,15	2,48	24,68	2,72	12,01			2,74	
YP_003578440	rcc02303	PQQ enzyme repeat family protein	2,62		1,41	5,05			2,33	2,72	1,89		1,81		2,27			

YP_003578441	rcc02304	hypothetical protein	6,51	3,22	4,64	17,72	4,18	6,80	3,05	37,13	3,73	12,32	1,42	15,04	2,99	38,93	2,78	23,69
YP_003578443	rcc02306	LysM domain-containing protein	6,19	15,81	4,26	12,09	5,88	4,46	3,90	4,52	3,49	2,85	2,70		3,04	40,11	2,63	
YP_003578448	sodB	superoxide dismutase	58,68	13,00	40,14	27,33	65,12	18,48	59,90	6,45	43,25	33,24	45,90	4,59	35,50	15,24	42,65	15,08
YP_003578450	soxG	sarcosine oxidase subunit gamma			1,15		1,97											
YP_003578451	soxA	sarcosine oxidase subunit alpha	2,60	3,69	2,28		3,39	11,94	2,71	20,57							2,65	
YP_003578453	soxB	sarcosine oxidase subunit beta	2,00	1,72	2,01	5,52			1,88	16,67			1,49		1,48		1,10	
YP_003578454	cycH	cytochrome c-type biogenesis protei					1,53		1,62	6,76	0,72		3,94		1,33		4,33	
YP_003578455	yqgF	holliday junction resolvase YqgF					1,05				0,88	15,59	1,46	7,05	1,21	28,48	1,41	
YP_003578459	prfC	peptide chain release factor 3	2,13	0,19	2,37	26,34	2,41	20,99	3,20				3,18		3,37		3,91	16,73
YP_003578462	rcc02325	HesB/YadR/YfhF family protein	6,32		5,69		6,13	24,21			9,99							
YP_003578479	rcc02342	hypothetical protein											2,03					
YP_003578480	rcc02343	hypothetical protein			1,35		1,45		1,87									
YP_003578485	rcc02348	cold-shock DNA-binding domain-con	6,67	11,62	5,11	7,67	6,31	14,76	2,38	25,37	4,85	10,09	5,25	1,03	3,54		3,87	12,12
YP_003578486	pdxH	pyridoxal 5'-phosphate synthase	1,58	29,62	1,42	26,00	6,77	73,92	4,60	78,68	1,25	15,77	1,94	6,62	1,34		1,19	
YP_003578487	fabI1	enoyl-ACP reductase	6,18	22,94	5,54	9,29	6,50	24,11	9,86	37,65	5,81	10,69	8,19	11,29	4,84	21,55	9,55	1,83
YP_003578489	gpt	xanthine phosphoribosyltransferase	2,09	1,38			1,72		1,94	8,62	1,73		1,73		1,85	4,89	1,94	
YP_003578490	rcc02353	class I/II aminotransferase	1,39		1,18	6,45	2,30	9,72	1,95	22,83	1,11	24,16	1,65					
YP_003578491	ppiD	peptidyl-prolyl cis-trans isomerase D	7,35	18,09	5,89	3,72	5,54	15,13	5,45	8,02	4,03	18,36	3,99	27,36	4,84	1,75	4,79	6,33
YP_003578492	trpE	anthranilate synthase component I	5,08	2,85	4,23	27,28	6,08	6,97	5,93	19,55	4,35	15,83	4,44	1,91	4,47	26,62	4,92	26,23
YP_003578494	trpG	anthranilate synthase component II	3,40										3,20					
YP_003578495	trpD	anthranilate phosphoribosyltransfera	5,24		6,27	2,86	6,42	26,49	7,00	13,38	4,62	32,29	4,96	10,05	4,98	44,72	6,62	8,49
YP_003578496	trpC	indole-3-glycerol phosphate synthase	2,63	0,60	1,79	25,38	3,24	16,48	3,51	10,80	2,15	12,11	3,07	5,49	2,19	25,08	3,92	
YP_003578497	moaC1	molybdenum cofactor biosynthesis p					1,78	12,92	1,79									
YP_003578498	lexA	LexA repressor	1,94		2,90	25,42	3,84		2,14	16,88	2,68		2,17		1,59		1,34	
YP_003578500	gltX2	glutamyl-tRNA synthetase	2,65		1,35		2,52		1,82		3,85		3,08		1,81		3,08	36,89
YP_003578501	gltA	citrate (Si)-synthase	36,73	15,81	27,68	18,04	44,88	19,04	52,74	7,27	31,29	21,57	34,97	8,08	50,26	6,05	25,12	10,17
YP_003578502	mdoG2	glucans biosynthesis protein G															0,76	
YP_003578507	rbtD	ribitol 2-dehydrogenase					2,52	50,29			1,60		0,78					
YP_003578510	rcc02373	monosacharide ABC transporter peri	9,15	66,99	8,26	26,99	7,37	50,81	4,50	20,95	15,91	41,73	5,97	23,80			2,56	
YP_003578516	rcc02379	CobW/HypB/UreG family cobalamin	0,74															
YP_003578519	mraW	S-adenosyl-methyltransferase MraW	3,17	14,27	1,55	24,36	2,70	14,35	2,97	4,89	2,53	42,13	3,02				2,39	22,66
YP_003578520	mraZ	protein MraZ	2,32				1,16		1,82						0,89		1,06	
YP_003578521	rcc02384	Mrp/NBP35 family protein	6,99	17,37	6,44	22,13	5,18	21,39	5,18	39,86	4,32	6,20	6,74	24,49	8,26	6,10	8,30	32,53
YP_003578527	rcc02390	alkane 1-monooxygenase	4,68	2,45	3,44	40,52	3,61	37,76	2,93	29,86	3,29	14,30						
YP_003578529	xseA	exodeoxyribonuclease VII large subu	3,72						0,81									
YP_003578530	purD	phosphoribosylamine--glycine ligase			1,69	25,96	3,13		3,43		2,39	18,39	2,82	4,53	2,42		4,42	27,56
YP_003578533	rpiA	ribose 5-phosphate isomerase A	6,74	9,01	5,42	10,33	7,02	9,46	6,23	3,63	4,98	5,30	6,49	12,38	4,30	8,77	4,96	17,40
YP_003578535	gor	glutathione-disulfide reductase	2,17	31,14	1,49								3,12		2,19		2,19	

YP_003578536	hflK	HflK protein	10,36	17,72	6,33	18,71	7,97	24,02	8,00	11,47	5,04	28,09	4,38	52,76	5,84	5,14	7,31	19,27
YP_003578537	hflC	HflC protein	8,35	9,51	6,58	22,28	8,92	11,54	7,00	13,43	4,08	21,64	4,01	55,56	5,41	12,89	5,57	40,63
YP_003578539	degP	protease Do	71,30	10,92	71,63	25,49	79,55	22,50	46,33	10,86	59,99	11,93	67,20	6,81	39,87	15,38	46,11	1,35
YP_003578542	purU	formyltetrahydrofolate deformylase	2,16						2,67	28,63			1,87				1,74	
YP_003578543	pgi	glucose-6-phosphate isomerase			1,23		2,14		1,83	12,72	1,53		2,12	5,60			2,00	20,40
YP_003578544	pgl	6-phosphogluconolactonase			1,81	8,38	1,58	8,14	2,19	24,32	1,23	4,43	2,27	12,01	1,55	39,22	1,39	25,46
YP_003578545	zwf	glucose-6-phosphate 1-dehydrogena	3,33	5,36	2,49	41,66	2,58	28,65	2,51	16,54	2,07		3,18	14,47	2,66	25,52	2,34	36,37
YP_003578547	rcc02410	hypothetical protein					1,25										4,14	
YP_003578548	edd	phosphogluconate dehydratase	3,45	8,27	2,48	35,71	4,24	12,38	4,53	12,40	2,65	28,23	4,94	27,36	4,84	12,55	3,24	13,55
YP_003578549	eda	KHG/KDPG aldolase	7,82	6,44	6,38	23,21	7,38	12,06	8,59	12,24	6,41	20,73	9,61	10,36	9,79	7,07	9,09	16,76
YP_003578554	dctP2	TRAP C4-dicarboxylate transport syst	68,48	15,02	82,67	22,34	87,27	25,23	131,94	4,50	79,61	19,04	90,59	10,13	44,29	14,01	52,64	17,20
YP_003578555	rcc02418	short-chain dehydrogenase/reductas			1,24		1,00		1,26	28,95	1,24		1,07	5,51				
YP_003578558	bglX	periplasmic beta-glucosidase					2,18		2,53				1,03				2,09	
YP_003578561	rcc02424	short-chain dehydrogenase/reductas	5,90	15,79	3,64	25,64	3,65	8,09	4,37	31,33	3,67	31,08	3,25	8,06	5,30	13,36	5,28	7,11
YP_003578562	surE	5'-nucleotidase SurE	3,04				2,08											
YP_003578563	pcm1	protein-L-isoaspartate O-methyltrans			1,19				1,42				1,60		0,91	4,33		
YP_003578564	rcc02427	M23 family peptidase	2,94	7,57	2,21	21,90	3,19	6,63	2,70	22,26	1,21	32,66	2,88					
YP_003578566	xthA1	exodeoxyribonuclease III			1,75	16,43	1,99		2,74	11,62	1,84	18,49	1,54				2,62	
YP_003578567	nahG	salicylate hydroxylase	0,45															
YP_003578568	dksA1	DnaK suppressor protein	15,37	3,93	11,10	17,70	12,05	26,53	6,15	9,99	9,79	7,18	6,73	30,04	9,26		8,27	46,57
YP_003578569	rcc02432	ATPase AAA			1,23	24,96	0,91		0,82		1,27							
YP_003578571	rcc02434	cephalosporin hydroxylase	1,87	2,69	2,07		2,59	15,71	2,41	23,81	2,13		2,21	4,13	1,79	17,82	2,73	23,59
YP_003578572	rcc02435	family 2 glycosyl transferase					1,30				0,98		1,50	7,29	2,04		2,13	38,68
YP_003578574	rcc02437	cytochrome P450 family protein	2,32	15,42	1,85	3,98	2,63	4,32	2,89	16,06	1,93	18,08	2,37	10,28	1,41	16,95	3,14	43,46
YP_003578577	rcc02440	AsnC/Lrp family transcriptional regul			2,12	18,97												
YP_003578579	livJ	branched-chain amino acid ABC trans	9,39	7,60	5,33	9,94	15,46	14,89	15,01	13,03	10,22	15,07	7,14	9,30	5,35	1,72	4,25	15,88
YP_003578585	aldH2	aldehyde dehydrogenase	3,91				3,20	10,22	2,55	2,41	1,57	30,19						
YP_003578589	potD4	polyamine ABC transporter periplasm	6,04	16,29	3,43	12,20	6,73	17,16	5,73	10,48	4,78	10,33	4,94	12,77	2,62		2,26	
YP_003578597	cspA2	cold shock protein CspA	149,71	10,86	111,31	17,86	119,10	8,22	91,74	5,13	96,53	18,50	59,04	18,43	90,09	10,59	83,07	4,38
YP_003578598	thrS	threonyl-tRNA synthetase	9,08	34,94	6,01	13,34	7,10	10,27	7,66	10,29	5,24	11,59	6,56	14,95	7,85	14,67	8,15	10,29
YP_003578601	proS	prolyl-tRNA synthetase	9,76	12,30	7,19	26,72	9,91	31,41	9,98	9,47	7,45	15,28	8,03	6,11	7,22	16,13	10,15	26,37
YP_003578602	rcc02465	hypothetical protein	0,84		1,75													
YP_003578605	rcc02468	thioesterase superfamily protein	3,55		2,30		3,09		2,84	11,44	2,60		2,23					
YP_003578606	rcc02469	ErfK/YbiS/YcfS/YnhG family protein															0,24	
YP_003578610	rcc02473	TonB-dependent receptor plug doma	1,71															
YP_003578613	ppaC	inorganic pyrophosphatase	25,92	18,61	19,17	0,69	26,35	5,25	24,68	4,24	21,29	7,20	27,03	12,20	14,91	1,62	20,93	6,39
YP_003578614	groS	chaperonin GroS	259,91	19,12	174,31	26,81	191,11	18,68	108,21	12,70	169,15	23,03	119,76	10,58	195,82	17,51	163,18	18,63
YP_003578615	groL	chaperonin GroL	132,85	0,75	92,70	5,21	113,21	7,09	103,43	4,82	103,32	4,58	110,33	7,61	118,35	1,48	129,38	2,23

YP_003578617	polA	DNA polymerase I			1,66		1,80		1,91		1,40	14,42	2,09	8,95			3,67	11,35
YP_003578619	rcc02482	HIT family protein	13,63	21,21	7,97	8,83	10,79	17,82	5,15	45,27	7,40	9,31	9,23	5,67	5,46	9,86	8,89	12,26
YP_003578623	rcc02486	carbohydrate/purine kinase	7,05	6,16	6,61	7,67	6,43	12,85	8,18	12,01	6,02	7,31	8,41	14,26	6,59	10,33	8,61	13,84
YP_003578627	rcc02490	OmpA family protein	13,64	2,84	20,01	13,27	14,62	22,76	14,69	11,18	9,26	13,59	14,75	18,97	15,10	7,51	22,44	23,30
YP_003578630	rcc02493	Crp/Fnr family transcriptional regulat											1,61				0,42	
YP_003578631	hemN2	oxygen-independent coproporphyrin	20,28	10,90	15,22	30,90	19,45	26,36	21,68	15,68	14,52	37,76	14,82	12,31	19,95	13,39	24,08	23,34
YP_003578637	rcc02500	ABC transporter periplasmic substrat	9,70	12,26	8,62	18,19	8,38	15,72	10,54	11,78	7,76	20,65	12,03	2,54	7,08	16,54	8,90	17,59
YP_003578638	cycY	cytochrome c	31,00	8,05	24,83	23,55	23,51	25,11	21,64	16,07	17,77	26,74	13,74	40,81	20,93	1,02	21,71	13,21
YP_003578639	pheA	prephenate dehydratase	2,06		2,75	26,50	3,94		3,56	11,50	2,62	41,10	2,49	7,61	2,30		3,04	
YP_003578640	rcc02503	hypothetical protein	1,32						1,17								0,80	
YP_003578643	rluB	ribosomal large subunit pseudouridir			2,93		1,36	4,01	2,08		1,77		2,59	14,16	1,97		2,43	
YP_003578644	rcc02507	hypothetical protein					4,07		2,71						1,71			
YP_003578646	rcc02509	TelA family toxic anion resistance pro	19,52	11,75	16,19	7,64	17,75	8,20	16,41	7,70	13,00	4,92	11,90	19,03	10,91	0,09	11,04	6,68
YP_003578650	cscK	fructokinase			0,93	31,92	0,59		0,97	27,53	1,24		0,96	14,15			0,52	
YP_003578652	rhlE	ATP-dependent RNA helicase RhlE											2,90		1,68		1,36	
YP_003578655	rcc02518	type 12 family methyltransferase	2,23						1,32									
YP_003578658	rcc02521	pyrimidine ABC transporter periplasm	61,55	10,30	57,37	18,97	98,98	14,47	97,96	3,48	81,72	19,17	83,48	13,63	30,20	1,31	30,49	3,25
YP_003578661	rcc02524	pyrimidine ABC transporter ATP-bind	2,05				1,60											
YP_003578662	dht	dihydropyrimidinase													3,87			
YP_003578665	rcc02528	dihydropyrimidine dehydrogenase					3,59											
YP_003578666	rcc02529	pyridine nucleotide-disulfide oxidore					1,67	3,88	2,92	16,10	1,31	2,64	0,71					
YP_003578667	pucB	light-harvesting protein B-800/850 su	29,13				15,17				21,73							
YP_003578670	pucDE	light-harvesting protein B-800/850 su	99,32	5,83	68,96	30,39	41,75	35,36	34,42	7,33	44,88	36,41	35,21	39,30	100,88	17,01	118,01	26,11
YP_003578674	cysK1	cysteine synthase	29,06	8,75	18,15	17,54	23,07	13,61	21,84	11,58	23,04	13,69	18,51	4,16	20,08	17,22	20,34	36,78
YP_003578675	trpB2	tryptophan synthase subunit beta	10,56	9,30	8,79	21,52	7,89	13,45	8,91	13,75	8,61	30,55	8,08	7,46	6,43	15,58	10,62	39,74
YP_003578685	rng	ribonuclease G			1,60						1,14		2,25					
YP_003578686	maf	septum formation protein Maf			0,45													
YP_003578687	infA	translation initiation factor IF	13,97		19,37						17,99		15,45				17,70	
YP_003578690	rcc02553	hypothetical protein	2,39		2,47	4,90	2,92	17,11	2,71	7,17	2,06		2,68	16,72	1,28		1,82	
YP_003578694	rcc02557	uncharacterized protein family UPF0	1,39	41,17	2,08	61,40	1,41	6,10	1,85	17,44	1,14		1,35	6,44			1,66	19,81
YP_003578695	hisD	histidinol dehydrogenase	5,21	44,42	4,92	38,67	5,14	20,57	5,27	22,37	3,99	25,59	5,44	8,82	3,74	30,04	4,49	36,11
YP_003578697	rcc02560	hypothetical protein	1,63	15,80	1,40	0,98	2,18	33,47	1,21	8,96	1,54	7,54	1,79		0,92	22,47	1,17	
YP_003578698	murA	UDP-N-acetylglucosamine 1-carboxyv	3,47	14,14	2,67	21,00	3,47	11,97	3,90	10,80	2,69	19,87	4,09	3,75	2,62	2,11	3,69	13,11
YP_003578702	rcc02565	hypothetical protein													0,36			
YP_003578704	rcc02567	resolvase					0,78											
YP_003578715	rcc02578	iron(III) ABC transporter periplasmic									1,10		2,02	13,99	2,64	22,01	1,55	
YP_003578720	lon	ATP-dependent protease La					3,17	29,18	4,03		3,19	19,52	4,88	1,33	5,09	8,83	5,50	
YP_003578721	hup3	DNA-binding protein HU	10,79	13,01	15,78	23,28	13,50	16,72	10,99	12,51	14,19	22,70	9,53	17,84	7,47	13,46	7,36	19,68

YP_003578722	ndh	NADH dehydrogenase_ cyclic nucleot			1,83	20,11	1,71	7,57	2,40	24,61			3,16				1,91	
YP_003578724	rcc02587	hypothetical protein			2,93		2,53		3,69		6,11	6,17	1,13					
YP_003578726	fadJ	fatty acid oxidation complex subunit	1,01				2,11	70,27	1,00	1,53	0,58	13,90	0,76	11,20	1,50		0,93	
YP_003578731	rcc02594	response regulator receiver protein			2,57													
YP_003578735	rcc02598	beta-alanine--pyruvate transaminase	11,07	4,78	9,66	7,85	14,37	14,55	16,18	14,50	9,11	23,68	11,54	4,09	9,28	12,02	11,61	21,12
YP_003578738	accB	acetyl-CoA carboxylase biotin carbox	15,82	12,28	11,06	33,12	15,39	28,43	5,22	21,23	13,21	31,61	6,80	6,26	5,16	14,12	6,03	33,42
YP_003578739	accC	acetyl-CoA carboxylase biotin carbox	5,86	17,87	5,53	10,71	6,22	22,71	7,02	10,68	5,44	18,30	7,38	9,17	5,55	19,35	7,69	24,89
YP_003578744	rcc02607	NDUFA12 family NADH ubiquinone o	3,73	11,93	1,32		4,15											
YP_003578745	clpX	ATP-dependent Clp protease ATP-bin	21,92	15,13	16,65	12,99	20,63	13,26	21,13	8,30	17,21	9,53	19,25	0,83	17,60	15,29	18,19	21,52
YP_003578746	clpP	ATP-dependent Clp protease proteol	29,79	8,73	22,15	16,79	31,38	21,01	30,63	13,83	23,78	27,95	27,91	8,72	26,85	18,57	31,25	34,10
YP_003578749	rcc02612	transthyretin family protein	2,26															
YP_003578750	rcc02613	chitin deacetylase					1,91	80,03	1,55	28,19					1,45			
YP_003578751	rcc02614	cupin domain-containing protein	7,12				2,23										2,06	
YP_003578753	rcc02616	hypothetical protein					1,71											
YP_003578754	ushA	5'-nucleotidase	4,95	12,93	6,06	15,11	5,18	13,30	9,19	8,51	4,10	36,38	5,25	3,89	3,33	6,49	3,86	3,64
YP_003578756	pyrD2	dihydroorotate oxidase	2,79	8,75	3,40	30,57	3,25	45,65	4,02	6,75	2,02		2,96	18,76	6,62	21,56	4,55	
YP_003578757	acsA2	acetate--CoA ligase			0,41		0,59		0,52	15,35			0,16	71,12			0,56	
YP_003578759	rcc02622	pyridoxamine 5'-phosphate oxidase	1,15															
YP_003578761	lysS	lysyl-tRNA synthetase	4,45	21,19	5,25		6,14	20,72	5,33	23,43	3,98	18,79	5,61	2,22	4,59	7,24	5,74	30,03
YP_003578762	rcc02625	hypothetical protein	4,33	2,47	3,08	10,98	4,18	23,20	2,22	8,93	2,56	34,98			2,05		2,44	
YP_003578771	rcc02634	response regulator receiver modulat	2,43	5,47	2,38		2,88	23,17	3,07	16,16	0,98		2,17	8,15			2,68	
YP_003578775	rcc02638	calcium-binding EF-hand domain-con									1,35						1,46	
YP_003578776	rcc02639	NUDIX superfamily hydrolase	2,86															
YP_003578779	rcc02642	aldo/keto reductase family oxidoredu			2,46								2,04	10,02				
YP_003578783	rcc02646	hypothetical protein			7,65	2,10							16,13	1,00				
YP_003578784	putA	bifunctional protein PutA					1,85				0,79							
YP_003578786	selD	selenide_ water dikinase			0,88	14,81			1,08									
YP_003578787	rcc02650	UspA domain-containing protein			3,36													
YP_003578789	rcc02652	RbsD/FucU transport protein family					2,21		1,00								2,42	
YP_003578790	glpK1	glycerol kinase							0,67								1,06	
YP_003578791	tktB	transketolase_ C-terminal subunit													1,96	19,01	1,98	8,61
YP_003578792	tktA	transketolase_ N-terminal subunit															0,15	
YP_003578793	rcc02656	hypothetical protein	0,60												2,70	13,65	2,99	29,97
YP_003578794	rcc02657	monosacharide ABC transporter peri	2,71		3,34	19,80					3,76	4,16			1,94		2,51	
YP_003578795	rcc02658	lipoprotein															1,08	
YP_003578799	rcc02662	class II aldolase/adducin N-terminal d	3,29		9,89	65,21	2,27	7,54	2,74	24,04	2,48		2,71	13,59	3,12	9,07	3,42	40,12
YP_003578800	rcc02663	DeoR family transcriptional regulator			0,26													
YP_003578801	mtnA	methylthioribose-1-phosphate isome	1,84														1,75	3,31

YP_003578803	rcc02666	alpha/beta fold family hydrolase	1,62		2,06	3,07	2,16	11,45	2,27	18,33			3,05	16,47	1,94			
YP_003578804	fabI2	enoyl-ACP reductase	3,35				2,80										2,08	
YP_003578805	fabB	3-oxoacyl-ACP synthase I	13,31	9,85	12,82	18,60	15,34	9,91	18,12	11,00	11,83	22,27	15,27	1,57	12,69	11,87	17,05	24,71
YP_003578806	fabA	3-hydroxydecanoyl-ACP dehydratase	6,85	28,74	4,48	18,72	6,18	24,96	6,96	9,87	4,73	23,19	5,04	1,90	4,33	23,41	6,95	
YP_003578808	efp	translation elongation factor P	75,13	19,73	75,23	46,68	72,35	32,97	44,35	12,81	62,85	25,21	65,84	21,13	45,58	20,36	51,27	38,07
YP_003578814	aspC2	aspartate aminotransferase	4,53	33,81	3,91	9,16	4,31	5,15	4,84	14,80	3,65	4,19	5,67	9,53	3,42	7,63	4,73	10,36
YP_003578816	rcc02679	pyridine nucleotide-disulfide oxidore	1,60	11,59	1,59	9,67	2,03	25,86	1,60		1,03	4,21	1,56	19,54	1,43	1,79	1,38	7,44
YP_003578818	rcc02681	cytochrome b561 family protein	4,49				5,06										5,52	
YP_003578819	rcc02682	cytochrome c'	18,82	57,35	22,77	24,74	25,34	58,51	9,70	5,03	42,40	59,94	16,96	12,61	13,78	2,98	15,94	14,27
YP_003578822	rcc02685	glutathione S-transferase	11,34	1,75	10,79	20,77	13,03	24,92	15,24	3,60	9,07	18,25	12,21	3,38	9,09		11,25	30,56
YP_003578824	hemK	protein methyltransferase HemK							3,23	35,01	1,03		2,04	12,93			1,67	
YP_003578825	rcc02688	hypothetical protein	5,90	14,82	4,80	13,62	8,31	37,19	4,36	13,33	6,99	26,82	6,25	24,74	6,02	3,11	5,90	24,10
YP_003578827	pdxA	4-hydroxythreonine-4-phosphate de															1,18	
YP_003578828	surA	chaperone SurA	2,57		1,02		1,58											
YP_003578832	pepA2	leucyl aminopeptidase	8,21	12,41	6,78	21,32	7,87	14,13	9,96	10,74	6,68	18,20	13,60	26,52	7,17	15,82	8,80	25,22
YP_003578837	rcc02700	lipoprotein					3,83											
YP_003578839	rcc02702	cytochrome c/b561 family protein	11,74	23,34	37,37	4,23	16,83	14,39	30,33	7,50	36,93	11,83	40,56	5,36	31,52	9,09	30,66	14,68
YP_003578841	rcc02704	NLP/P60 family protein	1,35	14,41			0,23				1,42						1,31	
YP_003578842	pepA3	leucyl aminopeptidase	10,09	18,59	8,37	26,47	9,83	21,11	12,21	18,14	7,24	31,08	8,60	12,08	9,41	20,57	11,09	20,03
YP_003578844	cynT	carbonate dehydratase	2,66		1,87	22,29	2,66	68,36	4,48	29,57	1,27		1,51				2,62	31,72
YP_003578847	rcc02710	hypothetical protein	26,06	7,42	32,43	17,52	27,69	15,11	23,48	4,88	24,99	6,71	41,99	3,27	22,12	6,50	27,35	3,24
YP_003578848	asd	aspartate-semialdehyde dehydrogen	15,32	5,60	12,02	13,25	14,93	8,00	17,58	5,41	14,32	14,20	16,59	7,77	16,31	12,18	17,93	2,77
YP_003578857	rcc02720	hypothetical protein					1,04											
YP_003578871	rcc02735	virulence-associated protein E			1,43				0,25		7,38							
YP_003578882	rcc02746	RND family efflux transporter subuni	1,66	13,04	1,16	12,00	8,54	113,82	1,05	13,89	1,26	9,46	1,15	24,43	0,91		0,68	11,38
YP_003578885	rpmG	50S ribosomal protein L33													17,42		18,27	
YP_003578886	rcc02750	hypothetical protein	11,27	4,56	11,01	17,59	7,56	24,97	9,80	7,76	9,46	15,34	11,50	15,91	5,18	3,98	6,80	8,88
YP_003578888	rcc02752	lipoprotein	0,85		2,00	20,95							1,28				1,49	
YP_003578889	gatA	glutamyl-tRNA(Gln) amidotransferas	9,94	2,05	7,96	22,40	10,30	18,08	11,32	10,55	7,56	16,18	10,46	4,89	9,08	19,53	12,03	23,71
YP_003578890	gatC	glutamyl-tRNA(Gln) amidotransferas															1,74	
YP_003578893	dnaX	DNA polymerase III subunit gamma/t	1,11										0,96					
YP_003578894	rcc02758	hypothetical protein	6,52	4,96	6,17	22,24	9,72	19,29	4,29	27,93	7,44	15,97	3,40	37,70	2,22		3,44	22,51
YP_003578900	rcc02764	hypothetical protein			19,15						7,13							
YP_003578901	ilvE1	branched-chain-amino-acid transami	13,51	17,23	8,44	20,15	12,50	20,22	11,44	9,93	9,88	26,28	10,99	11,04	10,38	9,99	12,07	5,41
YP_003578902	petP	transcriptional regulator PetP											1,24					
YP_003578903	petR	transcriptional regulator PetR					1,38											
YP_003578904	petA	ubiquinol--cytochrome-c reductase i	60,70	10,93	44,59	7,68	41,37	14,80	22,96	21,52	32,44	33,28	14,95	52,96	37,24	16,66	28,85	8,10
YP_003578905	petB	ubiquinol--cytochrome-c reductase c									8,32							

YP_003578906	petC	ubiquinol--cytochrome-c reductase c	65,77	7,42	55,74	27,12	51,79	32,01	53,12	19,66	40,24	28,59	30,97	59,01	44,46	6,26	55,85	28,12
YP_003578909	hisP	polar amino acid ABC transporter AT	1,71	8,19	1,56	10,19	1,49	0,02	1,78	7,10	1,02	35,92	1,88		0,72		1,17	32,56
YP_003578910	hisJ	polar amino acid ABC transporter pe	13,80	11,35	12,83	19,73	17,68	29,36	10,61	22,20	18,01	23,50	8,70	14,13	5,23	6,16	4,24	22,18
YP_003578913	glnA4	glutamine synthetase-4	8,29	8,53	7,36	26,06	10,81	19,77	12,30	12,60	6,69	14,23	8,80	17,29	7,61	15,84	9,94	26,71
YP_003578914	rcc02778	class I glutamine amidotransferase	3,38	8,88	3,23		5,65	19,07	5,25	14,75	3,90	25,70	4,56	5,69	3,16		3,48	26,36
YP_003578916	glnA5	glutamine synthetase-5	4,46				3,39											
YP_003578920	rcc02784	TrmH family RNA methyltransferase									1,14		0,78				1,33	41,81
YP_003578923	metB	O-acetylhomoserine aminocarboxypr	49,08	6,43	45,04	22,06	44,91	18,07	56,98	9,99	43,65	23,78	49,06	8,52	39,28	16,27	46,73	24,28
YP_003578924	rcc02788	hypothetical protein	4,85		4,14	17,06	3,80	20,69	3,54	5,47	2,93	18,03	3,30		4,33		3,60	
YP_003578926	rcc02790	CarD family transcriptional regulator	3,33	7,54	3,26	13,95	3,36	22,73	2,56	16,70	3,02	16,65	2,90	14,75	3,59	0,93	3,50	5,11
YP_003578929	rcc02793	helicase domain-containing protein	21,89	5,65	19,66	15,56	21,60	12,20	24,29	3,23	19,42	10,01	20,38	26,17	19,80	4,73	22,01	7,87
YP_003578931	rcc02795	sterol-binding domain-containing pro	20,46	27,46	18,40	56,69	20,72	43,64	8,68	18,08	18,62	56,11	15,49	44,55	9,50	28,05	10,50	31,30
YP_003578934	rcc02798	transglutaminase domain-containing			1,12		3,99											
YP_003578935	rcc02799	hypothetical protein			1,40		3,15	23,36	2,95	18,20	2,63		1,63	2,98	1,16		1,26	
YP_003578936	rcc02800	hypothetical protein	2,73	2,97	2,25	23,36	5,01	16,85	4,80	21,27	2,72	15,66	2,14	13,77	2,32		2,94	30,90
YP_003578937	pepF	oligoendopeptidase F	5,09	25,30	3,71	18,80	6,71	21,85	6,25	11,08	3,72	20,67	4,87	10,70	3,42	19,28	6,28	51,09
YP_003578941	rcc02805	hypothetical protein	2,47	12,03	2,01	48,73	2,64	23,93	2,46	26,07	1,66	50,82	1,43	27,22	1,47	6,02	2,70	26,62
YP_003578942	rcc02806	hypothetical protein					0,23											
YP_003578944	rcc02808	hypothetical protein					12,66				6,66							
YP_003578946	rluD	ribosomal large subunit pseudouridir									0,38							
YP_003578948	gfo	glucose--fructose oxidoreductase							1,63	16,57			1,39	35,59				
YP_003578949	lrp	leucine-responsive regulatory protei	2,35	7,97	1,50		2,53	13,63	1,80	29,65	2,17		1,77	32,78	1,18	31,88	2,25	32,43
YP_003578950	trxB	thioredoxin-disulfide reductase	5,06	10,48	6,42	11,36	7,68	3,81	8,56	28,88	7,27	3,46	7,68	15,43	6,73	1,64	9,26	10,97
YP_003578952	sat	bifunctional sulfate adenylyltransfera	16,27	4,49	13,41	26,59	14,92	23,24	15,95	13,25	15,79	23,90	18,33	11,38	22,30	14,13	22,73	5,66
YP_003578954	ibpA	small heat shock protein IbpA			1,91								1,76		1,51			
YP_003578955	rcc02819	hypothetical protein	5,91	3,24	2,84		8,77	25,05	2,68		6,74	30,47			1,28		2,92	
YP_003578956	purE	phosphoribosylaminoimidazole carbo	5,72	27,86	4,53	8,71	4,86	16,55	6,01	10,81	4,85	8,71	5,99	3,94	5,18	16,48	6,13	2,13
YP_003578957	purK	phosphoribosylaminoimidazole carbo					2,00	43,10									5,41	
YP_003578959	prs	ribose-phosphate diphosphokinase	7,42	24,73	5,22	25,99	5,90	27,53	5,83	12,62	5,24	32,17	7,75	14,01	5,33	13,75	8,07	11,26
YP_003578960	rcc02824	DSBA family oxidoreductase	2,61	3,11	2,73	26,59	3,66	18,95	4,42	16,43	1,86	2,25	2,93	14,50	2,48	1,66	2,57	25,39
YP_003578961	rcc02825	alpha/beta fold family hydrolase	2,55		2,56	13,86	2,49	14,44	2,77	5,81	2,39	31,22	2,16	21,79	1,25	1,55	2,14	
YP_003578962	fumC	fumarate hydratase	20,22	3,75	14,80	11,40	17,99	7,62	19,29	9,76	15,45	15,01	16,57	7,73	16,71	10,37	22,27	46,56
YP_003578963	rcc02827	hypothetical protein	4,19	8,15	4,96	28,66	6,55	19,09	5,89	26,28	5,09	44,17	4,37	2,71	5,45	32,15	6,33	
YP_003578964	rcc02828	hypothetical protein			3,30	3,28	4,49	42,33	4,11		3,90	2,38					2,49	
YP_003578965	rcc02829	hypothetical protein													0,39			
YP_003578968	rcc02832	PTS system_ IIA component	0,77		1,25	15,28	2,01	23,13	2,50	33,31			1,94	41,36			1,21	
YP_003578972	chvI	transcriptional regulator ChvI	2,52		2,50	15,38	2,96	11,69	2,97	17,25	2,66	15,24	2,43	11,94	2,88	11,42	3,27	16,08
YP_003578973	pckA	phosphoenolpyruvate carboxykinase	31,90	8,37	27,65	15,47	39,66	22,65	38,85	5,88	25,81	16,50	29,84	5,50	24,31	13,40	38,69	35,19

YP_003578974	rnr	ribonuclease R	1,92	30,84	1,35	34,86	1,25		3,76	90,90	5,37	28,39			0,95		4,76	82,24
YP_003578975	mobA	molybdopterin-guanine dinucleotide					1,08		0,67								0,61	
YP_003578995	acsA3	acetate--CoA ligase	20,26						0,98				2,38	33,17			0,28	
YP_003579002	pflB	formate C-acetyltransferase			1,33		2,46	2,49	3,22	16,74			1,97	8,96			1,10	
YP_003579007	glcD	glycolate dehydrogenase_ subunit Gl	13,06		15,81		16,73	22,67	10,56	87,48	18,99		20,15				24,52	
YP_003579008	rcc02872	hypothetical protein	1,72	17,20	1,41		2,68	24,46	1,03		2,97		0,99	19,82			0,95	
YP_003579010	kpsE2	capsule polysaccharide export inner-			1,57		1,29		0,54	30,18					0,70			
YP_003579012	rcc02876	DeoC/LacD family aldolase	8,17	28,78							6,49		7,72		7,89		5,63	
YP_003579013	rcc02877	monosacharide ABC transporter peri	11,14	16,13	11,01	17,61	6,99	24,33	6,03	1,76	12,89	5,91	9,93	12,68	6,35	5,40	6,70	5,70
YP_003579020	rcc02884	FGGY family carbohydrate kinase	0,78															
YP_003579030	mdtE	multidrug resistance protein MdtE	4,11	30,52	1,99	19,98	2,15	14,63	1,29	11,01	2,06	11,60	1,28	12,00	1,91	4,86	1,38	18,32
YP_003579031	mdtF	multidrug resistance protein MdtF	3,07	1,13	4,36	23,98	3,10	39,00	4,68	7,30	3,24	35,90	3,24					
YP_003579032	baeR	transcriptional regulatory protein Ba	8,33	5,75	9,20	20,25	11,19	7,70	13,58	12,78	10,33	11,60	9,47	5,22				
YP_003579033	rcc02897	sensor histidine kinase	3,44		1,79		2,72		1,67	5,78	1,66		1,80					
YP_003579034	rcc02898	ABC transporter ATP-binding/permea	12,64	6,51	8,88	28,67	11,07	33,76	9,32	8,43	5,94	33,17	3,94	61,99				
YP_003579063	rcc02929	hypothetical protein					0,90		1,90									
YP_003579066	pinE	DNA-invertase	2,86															
YP_003579073	rcc02939	hypothetical protein	2,46	14,52	2,86	8,88	2,91	24,27	2,69	28,75	3,03		3,86	34,96	1,79	17,61	2,74	6,72
YP_003579076	rcc02942	hypothetical protein					3,49											
YP_003579083	rcc02949	hypothetical protein					0,59		0,83									
YP_003579087	rcc02953	phage integrase	1,40															
YP_003579088	rcc02954	mechanosensitive ion channel family							2,02		0,86		2,95					
YP_003579089	cysK2	cysteine synthase	18,65	18,63	16,06	17,21	17,89	10,67	22,35	12,79	14,91	11,47	20,51	13,40	16,99	11,65	18,99	15,47
YP_003579092	phrB	deoxyribodipyrimidine photo-lyase			3,07		3,72						0,79		1,40	45,45	1,62	
YP_003579093	potD5	polyamine ABC transporter periplasm	10,66	37,89	16,72	39,97	21,91	22,16	14,93	12,75	13,79	44,52	13,54	48,43	5,32	4,94	6,57	
YP_003579094	neuB	sialic acid synthase	3,57		3,66	16,23	4,84	9,02	5,45	9,88	4,18	20,88	4,51	7,91	3,63		3,08	
YP_003579095	rcc02961	family 25 glycosyl transferase	0,60		0,41		0,51								0,40			
YP_003579098	webC	UDP-N-acetylglucosamine 2-epimera					1,23		1,04		2,18		1,30				1,19	
YP_003579099	rcc02965	oxidoreductase family NAD-binding F	1,29		1,03		1,19		1,16				2,05				1,42	
YP_003579100	rcc02966	short-chain dehydrogenase/reductas			2,95	27,16	4,49	10,03	4,83	19,58	2,84		4,11				4,68	29,89
YP_003579101	rcc02967	cytidylyltransferase															2,32	
YP_003579104	atpC	ATP synthase F1 subunit epsilon	50,90	15,14	38,14	31,10	32,23	11,10	18,60	36,69	29,16	21,07	12,65	31,53	27,56	21,17	21,53	40,32
YP_003579105	atpD	ATP synthase F1 subunit beta	214,42	2,93	185,76	14,09	178,55	24,49	219,21	19,10	163,02	19,25	119,68	39,54	213,79	3,01	196,81	16,17
YP_003579106	atpG	ATP synthase F1 subunit gamma	26,19	3,49	17,94	29,97	17,86	35,44	18,22	10,24	13,76	32,67	10,64	39,55	19,10	9,00	20,48	26,41
YP_003579107	atpA	ATP synthase F1 subunit alpha	127,87	9,60	99,26	19,74	99,37	34,47	113,04	13,59	77,70	27,15	59,83	40,60	120,17	8,23	131,28	31,60
YP_003579108	atpH	ATP synthase F1 subunit delta	46,99	14,76	33,90	4,05	27,66	15,27	23,79	14,32	23,27	11,17	15,93	26,19	30,56	19,79	28,83	3,42
YP_003579110	gloB	hydroxyacylglutathione hydrolase	3,87												2,05			
YP_003579111	clpA	ATP-dependent Clp protease ATP-bir			2,44	13,70	2,65				4,82		8,70		3,77	15,85		

YP_003579113	rpsD	30S ribosomal protein S4	57,95	3,51	75,66	8,95	77,09	19,56	62,23	6,55	78,46	22,63	72,68	2,02	91,69	16,21	89,66	11,63
YP_003579115	hisC1	histidinol-phosphate aminotransfera	8,54	50,83	4,57	10,67	5,79	11,76	6,16	10,12	4,04	20,41	5,55	4,34	4,78	12,97	5,36	11,52
YP_003579124	rcc02990	YbaK/EbsC family protein											0,49					
YP_003579125	hadH	3-hydroxyacyl-Coa dehydrogenase/3	3,66	0,60	3,13	28,11	2,61	7,95	3,29	1,17	2,67	29,72	3,54	9,94			1,13	
YP_003579126	atoB1	acetyl-CoA acetyltransferase	1,75	49,40	1,88	19,83	1,70	22,51	1,71	12,53	1,64	44,69	2,24	10,10	1,66		1,89	
YP_003579127	ilvD	dihydroxy-acid dehydratase	12,06	16,80	9,34	17,37	11,33	14,32	12,71	10,94	8,48	19,97	10,20	4,37	10,31	13,48	12,64	26,87
YP_003579135	proA	glutamate-5-semialdehyde dehydrog	6,53		3,14	29,03	5,89		3,71	9,43	3,27	8,83	3,71	49,98	2,18		3,86	
YP_003579138	rcc03004	DeoR family transcriptional regulator											0,69					
YP_003579139	rcc03005	diguanylate cyclase/phosphodiestera	1,17		1,12	13,19	2,44		2,96	14,14	3,05		0,55	37,75			0,68	
YP_003579147	rcc03013	DeoC/LacD family aldolase	6,44	56,26	7,88	25,88	9,45	17,16	19,87	59,39	5,78	30,28	6,40	51,18	4,22	63,18	8,27	64,95
YP_003579155	dctP3	TRAP C4-dicarboxylate transport syst	397,34	14,56	519,90	4,66	266,29	15,20	258,88	25,61	483,38	10,50	591,79	4,85	104,12	12,88	269,03	23,54
YP_003579164	fdhE	NAD-dependent formate dehydroger									1,04							
YP_003579167	fdhB	NAD-dependent formate dehydroger							0,69									
YP_003579172	thyX	thymidylate synthase ThyX											1,34					
YP_003579173	rcc03042	lipoprotein	2,10	14,75	2,33	25,50	2,27	14,03	2,22		1,85		1,67	10,06	2,18	8,16		
YP_003579175	gloA	lactoylglutathione lyase	2,82		1,87		3,24				3,60	17,10						
YP_003579176	rcc03045	hypothetical protein	4,27		1,11		4,76										5,70	
YP_003579177	engD	GTP-dependent nucleic acid-binding			4,32		3,09		3,59	13,79	2,18	33,95	5,31	7,93	5,04		5,03	9,85
YP_003579178	trpA	tryptophan synthase subunit alpha	5,45	6,25	5,29	6,22	6,15	7,97	5,14	28,78	5,07	8,87	6,17	19,97	4,80	2,48	4,95	23,11
YP_003579179	lldD	L-lactate dehydrogenase	3,21	22,27	1,33	9,69	2,59	38,02	2,06	26,63			1,71					
YP_003579181	rplY	50S ribosomal protein L25	63,64	5,24	82,64	10,17	65,66	3,89	86,87	0,98	80,14	10,84	90,94	10,72	125,66	4,65	110,11	9,46
YP_003579185	rpoD	RNA polymerase sigma factor RpoD	5,06	12,91	3,90	27,57	5,00	4,63	5,41	11,18	4,00	26,93	4,98	14,77	4,53	8,32	5,64	
YP_003579192	hom	homoserine dehydrogenase	4,93	9,50	4,55	30,15	6,41	17,04	6,50	18,27	4,51	21,52	5,97	8,44	5,45	16,31	5,61	0,97
YP_003579193	glpX	fructose-1_6-bisphosphatase	4,67	21,12	6,38	23,83	5,49	16,10	7,13	3,42	6,30	16,37	8,74	11,13	6,35	15,69	9,68	8,12
YP_003579198	rcc03067	hypothetical protein									0,46				1,25	11,11		
YP_003579204	nusB	transcription antitermination factor N	2,46	13,75	2,76	19,62	2,62	22,65	1,84	33,03	2,22	6,61	2,57	4,05	1,69	22,92	1,54	
YP_003579205	ribH	riboflavin synthase subunit beta	6,21	5,71	4,27	24,30	5,16	5,67	4,60	16,78	4,01	13,54	4,78	18,61	4,31	11,22	4,45	5,50
YP_003579206	ribAB	3_4-dihydroxy-2-butanone 4-phosph	14,81	8,86	10,77	4,00	13,58	9,15	12,28	6,11	11,26	10,11	13,24	10,23	12,07	13,57	15,11	23,92
YP_003579207	ribE	riboflavin synthase subunit alpha			4,04	21,10	4,43	20,75	3,44	9,40							3,33	
YP_003579209	rcc03078	polysaccharide biosynthesis/export f					0,90											
YP_003579212	nrdR	transcriptional regulator NrdR					3,05		2,00				1,61	7,77			2,24	
YP_003579222	mtnP	S-methyl-5-thioadenosine phosphory	3,38	11,95	3,20	18,79	3,98	14,75	3,99	16,51	3,46	18,96	4,79	11,22	2,70	25,83	3,68	11,49
YP_003579223	apt	adenine phosphoribosyltransferase	3,43		3,87	27,04	4,23	13,34	5,57	9,55	3,15	5,16	4,90	4,48	3,18		5,38	
YP_003579224	rcc03093	D-2-hydroxyglutarate dehydrogenase	0,89				1,47											
YP_003579225	rimJ	ribosomal-protein-alanine N-acetyltr			0,59				1,37									
YP_003579226	rcc03095	M16 family peptidase			2,37		2,25		2,45	15,73			2,69	10,01			2,85	20,21
YP_003579227	thrC	threonine synthase	7,38	3,40	5,91	18,44	7,12	11,13	8,16	15,98	5,89	24,73	7,58	3,98	7,61	11,38	9,07	6,08
YP_003579228	tldD	protein TldD	0,67				0,93								5,11		0,95	

YP_003579231	topA	DNA topoisomerase I	4,44	2,57	3,89	28,58	5,33	18,84	4,39	13,78	3,15	31,38	3,63	5,34	4,43	13,28	5,12	24,25
YP_003579232	scoA	succinyl-CoA:3-ketoacid-CoA transfer	8,93	6,48	7,81	13,68	9,52	15,64	12,34	5,65	6,97	10,33	9,33	10,82	3,99	14,95	6,64	23,45
YP_003579233	scoB	succinyl-CoA:3-ketoacid-CoA transfer	29,79	18,46	28,06	15,61	32,25	19,75	31,10	1,39	26,48	7,48	25,10	7,49	13,15	1,78	17,74	6,32
YP_003579238	hisC2	histidinol-phosphate aminotransfera			1,18		3,23	59,74	2,35				2,08	12,05			0,57	
YP_003579242	gcvT2	glycine cleavage T protein	2,69	21,82	2,34	10,79	1,90	6,81	2,56	26,25	2,05	6,41	3,22	12,80	1,68		2,24	16,38
YP_003579245	rcc03114	TolC family type I secretion outer me	3,46		3,64		2,80		2,05				3,34		2,35		1,62	
YP_003579246	pcm2	protein-L-isoaspartate O-methyltrans	12,14	9,44	10,21	9,31	10,87	13,74	8,70	0,88	8,96	5,47	8,76	13,49	11,29	12,11	7,53	18,56
YP_003579251	rcc03120	ABC transporter ATP-binding protein	5,14	2,56	8,66	20,46	6,70	21,21	11,11	9,70	8,33	15,10	11,12	2,38	10,22	2,01	13,20	23,58
YP_003579254	lepA	GTP-binding protein LepA	5,56	3,17	5,65	33,80	5,25	10,98	6,86	14,45	4,59	23,61	6,25	9,73	7,05	7,43	8,32	5,28
YP_003579255	rcc03124	hypothetical protein	8,20	30,15	10,26	9,73	9,44	2,73	15,25	15,43	7,13	1,23	9,74	14,77	4,81	6,81	4,45	
YP_003579256	rcc03125	heavy metal transport/detoxification					1,66	13,79			1,02				1,02			
YP_003579258	etp	protein-tyrosine-phosphatase									0,98							
YP_003579260	rpmB	50S ribosomal protein L28	23,40	10,37	44,98	6,23	25,16	20,31	34,31	6,83	42,02	7,90	46,20	9,17	47,11	1,09	52,99	11,40
YP_003579261	argK	LAO/AO transport system kinase													0,93			
YP_003579262	rcc03131	hypothetical protein			1,09													
YP_003579265	argF	ornithine carbamoyltransferase	2,83				3,92	21,07					2,78				2,15	
YP_003579266	argD	acetylornithine aminotransferase	3,49	11,37	3,41	18,85	4,88	32,69	5,38	20,99	3,44	25,90	5,36	7,64	4,54	18,51	4,25	4,56
YP_003579270	rcc03139	ABC transporter ATP-binding protein			2,55		2,55				0,54				0,61		1,94	
YP_003579271	fadD	long-chain-fatty-acid--CoA ligase	4,12	15,71	3,59	17,68	3,40	10,31	4,07	6,33	2,73	21,74	3,38	14,43	2,34	13,47	4,66	40,78
YP_003579272	cspD	cold shock-like protein CspD	173,01	18,09	255,12	28,93	185,19	24,20	152,13	17,00	209,23	34,08	378,58	10,21	288,96	25,24	269,40	25,63
YP_003579273	rcc03142	MiaB family RNA modification enzym									2,13		2,20	25,88	1,96		1,82	
YP_003579277	gabT1	4-aminobutyrate aminotransferase	12,09	13,16	10,65	16,28	15,79	9,79	16,10	11,06	10,80	11,49	12,71	7,85	12,34	18,05	15,38	24,63
YP_003579282	pheT	phenylalanyl-tRNA synthetase subun	9,66	8,71	8,15	22,85	9,79	15,30	11,10	10,05	8,38	28,21	8,26	7,67	9,60	12,60	10,92	11,26
YP_003579284	pheS	phenylalanyl-tRNA synthetase subun	7,10	6,08	5,55	23,51	6,53	17,91	8,20	13,26	4,67	40,44	5,31	13,98	5,92	17,97	6,66	4,34
YP_003579285	rplT	50S ribosomal protein L20	41,22	14,73	36,18	19,83	37,02	25,57	17,51	9,82	31,79	19,65	30,30	34,62	61,26	10,12	52,03	23,53
YP_003579286	rpmI	50S ribosomal protein L35	20,81						17,70		22,13				32,54		20,84	
YP_003579288	pykA2	pyruvate kinase			1,88	30,35	2,43	10,40	4,67				2,48		2,39	11,40	2,70	15,74
YP_003579292	dat	D-amino-acid transaminase	11,88	11,52	10,67	2,08	12,90	5,65	14,17	3,86	12,00	6,78	14,14	8,53	10,14	4,03	13,58	13,64
YP_003579293	rcc03162	mandelate racemase/muconate lacto	1,79	2,21	2,98		2,03	3,44	1,95	11,31	1,96	14,20	2,49	8,86	2,34		1,79	26,23
YP_003579294	rcc03163	hypothetical protein	7,25	9,43	4,75	13,56	14,24	9,86	13,13	9,99	8,36	13,29	7,26	0,73	9,34	9,67	8,87	16,61
YP_003579296	accA	acetyl-CoA carboxylase carboxyl tran	4,29	22,02	5,65	20,87	6,08	11,76	6,94	8,88	3,96	20,08	5,31	8,41	4,18	16,11	5,94	15,63
YP_003579298	rcc03167	hypothetical protein											1,64					
YP_003579302	lysA	diaminopimelate decarboxylase	3,17	6,99	3,07	27,62	3,70	23,55	3,36	20,15	2,59	19,84	3,36	13,43	2,81		2,79	24,70
YP_003579305	argH1	argininosuccinate lyase	3,05	1,86	2,95	1,40	2,81	1,06	3,62	16,99	2,41	26,93	3,20	17,45	3,06	12,77	3,67	20,61
YP_003579306	dsbE	thiol:disulfide interchange protein	1,95	7,96	3,20	17,23	2,40	22,83	4,39	14,31	3,29	9,08	3,75	11,05	2,64	4,67	2,76	6,32
YP_003579309	atoB2	acetyl-CoA acetyltransferase	43,89	12,15	43,13	16,74	41,62	14,74	58,84	9,74	37,32	21,45	40,55	5,10	26,55	17,36	37,50	17,81
YP_003579310	phbB	acetoacetyl-CoA reductase	7,86	1,19	5,55	20,17	5,60	1,37	5,60	9,64	5,03	15,55	5,59	16,46	2,82	19,44	4,99	18,83
YP_003579312	rcc03181	hypothetical protein	21,25	11,95	25,41	38,40	23,51	35,69	12,73	2,76	30,16	35,39	16,71	12,86	9,99		20,96	

YP_003579315	ispB	octaprenyl-diphosphate synthase	4,80	8,15	4,69	13,19	5,64	18,20	5,33	15,34	3,97	15,45	5,33	18,70	3,49	2,66	5,12	7,56
YP_003579319	folD2	bifunctional protein FolD	7,57	12,37	6,75	16,86	8,45	4,75	8,93	11,68	6,59	14,83	9,43	22,92	7,61	12,89	8,34	6,40
YP_003579321	fhs	formate--tetrahydrofolate ligase													6,56			
YP_003579323	ftsH	cell division protease FtsH	9,51	5,05	7,17	30,01	7,87	41,81	7,06	7,31	4,04	40,95	6,99		6,51	10,91	8,71	26,28
YP_003579325	rcc03194	hypothetical protein									1,18							
YP_003579326	rcc03195	OmpA/MotB domain-containing prot	7,77	8,92	8,88	19,03	9,30	17,70	7,53	11,20	4,34	24,94	6,74	30,96	5,92	6,93	9,26	8,72
YP_003579327	tolB	TolB protein	12,28	4,27	15,34	10,98	13,58	7,74	18,62	11,24	14,14	6,37	18,77	5,29	14,41	10,19	17,92	29,07
YP_003579330	tolQ	TolQ protein	3,78	17,78			2,89		2,48				1,88					
YP_003579332	rcc03201	hypothetical protein	2,95		2,43	25,58	3,47	30,76	4,20	4,52	1,97		2,77	7,67	1,94		3,05	33,65
YP_003579333	glpK2	glycerol kinase	0,58		1,50				1,78		0,68		0,91				0,61	
YP_003579335	ileS	isoleucyl-tRNA synthetase	19,08	10,90	14,39	19,80	19,76	12,93	22,34	12,48	15,43	20,10	16,00	4,76	15,28	14,76	16,40	6,31
YP_003579341	talC	transaldolase	23,16	11,78	17,86	14,55	22,63	8,44	24,22	4,34	21,07	10,26	26,11	6,50	22,97	7,65	29,26	14,19
YP_003579343	prmA	ribosomal protein L11 methyltransfe	2,31		2,14		2,41		3,14	16,97	2,17	32,87	3,35	19,98	2,48	11,51	3,33	21,81
YP_003579348	ruvA	holliday junction DNA helicase RuvA					2,72						1,31				2,35	
YP_003579352	rcc03221	hypothetical protein			1,04		1,10		2,01	14,64	1,20		0,51				0,95	
YP_003579356	rcc03225	NAD-dependent epimerase/dehydrat			0,59													
YP_003579357	rcc03226	polysaccharide biosynthesis/export f			1,84	1,98	0,91		1,22	34,38	0,61		1,43					
YP_003579358	ilvE2	branched-chain-amino-acid transami	1,80		1,76	33,33	2,85		2,69	6,48	2,10	14,00	2,30	19,03	2,59		3,19	3,76
YP_003579359	rcc03228	universal stress family protein			1,04		1,69	29,41	2,24	24,58			1,40	19,02				
YP_003579360	rcc03229	NifU domain-containing protein	11,30	10,04	11,25	21,15	11,28	21,81	10,15	4,90	10,95	10,58	9,63	15,56	9,22	1,62	8,58	18,05
YP_003579363	rcc03232	basic membrane lipoprotein family	46,88	0,78	38,51	6,69	43,54	21,71	38,77	10,01	29,54	16,51	20,48	36,58	24,22	1,03	28,75	12,19
YP_003579367	punA	purine-nucleoside phosphorylase	3,63	17,33	3,58	11,83	3,66	8,42	3,79	22,20	2,85	6,05	3,64	18,32	1,65	12,18	2,01	8,31
YP_003579370	rcc03239	AsnC/Lrp family transcriptional regul			4,28	0,32	7,11				4,53		6,06					
YP_003579371	rcc03240	ATPase AAA					1,03		0,76				1,94	50,12				
YP_003579373	rcc03242	hypothetical protein			19,89	9,33	9,10	6,40	25,33	6,06	15,32		17,75				6,79	
YP_003579383	cydD	cysteine ABC transporter ATP-binding			0,56				1,07									
YP_003579388	msrA1	peptide-methionine-(S)-S-oxide redu									0,43							
YP_003579391	rcc03260	NAD-dependent epimerase/dehydrat			0,83				1,54									
YP_003579392	rcc03261	TOBE domain-containing protein	161,03	12,05	2,96		86,03	27,70	16,22		164,96	16,47	18,49	23,05	33,14	11,07		
YP_003579393	rcc03262	hypothetical protein	7,35	6,51	6,56	60,39					4,61	28,15	6,30	19,83	11,47	18,27	5,55	22,24
YP_003579394	rcc03263	NifT/FixU family protein	17,83	48,19	11,46	12,35					9,92	8,59	7,27		21,01	10,13	7,77	52,60
YP_003579398	nifA2	Nif-specific regulatory protein	9,25	39,75	12,44	32,13					8,33	25,64	9,35	13,14	7,06	11,38	10,39	22,00
YP_003579399	nifW	nitrogen fixation protein NifW	2,95												4,41	18,53		
YP_003579401	nifS	cysteine desulfurase	2,06	8,28	1,37	4,68									2,22	0,59	1,85	17,68
YP_003579402	nifU2	nitrogen fixation protein NifU	13,04		9,04						1,84		7,33		12,05	9,02	7,12	
YP_003579403	rcc03272	HesB/YadR/YfhF family protein	8,00	0,93							4,15	7,41	2,20		9,02	2,38	2,31	
YP_003579406	fdxB	ferredoxin III	15,12	18,01	6,43	12,18					6,27	13,34	7,15	7,24	10,12	9,47	3,88	28,63
YP_003579407	rcc03276	hypothetical protein	12,52								9,07	50,44			3,55		3,45	

YP_003579408	rcc03277	hypothetical protein	18,71	10,70	14,16	10,29					15,93	2,27	17,02	13,23	20,34	6,12	9,01	22,52
YP_003579409	nifX	nitrogen fixation protein NifX	35,00	9,00	25,02	5,48					24,45	4,21	21,57	10,07	36,49	10,54	15,14	22,27
YP_003579410	nifN	nitrogenase molybdenum-iron cofact	3,55	23,09	1,95	31,04					2,39	17,18			5,58	39,99	1,42	
YP_003579411	nifE	nitrogenase molybdenum-iron biosy	4,58	12,07	2,00	5,28					3,14	35,11	2,02	14,59	5,99	18,77	1,76	
YP_003579412	rcc03281	peroxiredoxin	5,20	25,29	3,67	25,13	2,51	12,35	1,98	18,98	5,85	30,28	5,69	15,76	16,62	10,16	9,71	46,22
YP_003579416	fdxC	ferredoxin IV	19,46	21,89	9,93	13,96					10,67	19,57	10,21	17,67	14,66	8,91	7,20	21,50
YP_003579417	norV	anaerobic nitric oxide reductase flavo	3,54		2,94										5,77	11,68		
YP_003579419	rnfB	electron transport complex protein R	2,29															
YP_003579422	rnfG	electron transport complex protein R	5,77	7,19	2,72	3,66					1,26		1,22	26,47	3,13	2,78	2,26	
YP_003579430	rcc03299	hypothetical protein	0,98															
YP_003579434	dapE	succinyl-diaminopimelate desuccinyl	2,74	7,75	2,68	6,74	3,13	4,88	3,21	10,54	2,62	11,05	3,15	18,58			2,44	
YP_003579437	rcc03306	acyl-CoA dehydrogenase_ medium-c			2,09										1,61			
YP_003579441	era	GTP-binding protein Era	2,60		2,30	26,65	2,85		2,70		0,66		2,50	0,21	1,87	11,72	2,44	3,78
YP_003579443	lepB	signal peptidase I	3,63	45,94	4,68	30,67	4,72	32,06	4,87	5,81	2,79	44,80	3,36	55,06	4,52	14,19	4,36	13,70
YP_003579446	pdxJ	pyridoxine 5'-phosphate synthase	8,22	1,03	7,45	18,68	8,82	17,74	7,86	27,39	6,35	8,66	8,56	8,28	4,53	36,87	13,36	51,84
YP_003579448	spoT	GTP diphosphokinase/guanosine-3'_	3,49		3,41	42,35					1,05		5,91					
YP_003579449	rpoZ	DNA-directed RNA polymerase omeg	3,37	12,39			2,24	7,28			1,25		0,39		1,79		2,05	
YP_003579451	rcc03320	hypothetical protein	12,33	15,49	6,55	24,43	10,40	16,65	9,59	10,20	4,76	42,18	7,76	1,09	8,82	17,86	11,95	25,32
YP_003579453	rcc03322	GAF domain-containing protein			4,16	0,64	6,35		3,86	12,33	4,25	0,14	4,55	13,49	2,44	1,72	2,86	
YP_003579457	rcc03326	lipoprotein					0,83											
YP_003579458	lolA	outer membrane lipoprotein carrier			4,41	23,96	2,60		3,08		4,17	16,66	6,36	16,75	2,65		3,50	
YP_003579460	rcc03329	class I/II aminotransferase	2,46	24,69	1,58	49,98	2,59	6,16	2,05	4,20	1,59		2,17	19,81			2,63	
YP_003579461	rcc03330	amidase	2,07	13,22	2,40	16,20	2,39	1,69	2,40	16,25	2,10	1,03	2,66	20,20	2,27	8,36	2,49	0,84
YP_003579462	ubiF	2-octaprenyl-3-methyl-6-methoxy-1_			1,82		2,88						1,14					
YP_003579464	rcc03333	S16 family peptidase			1,40		2,34		0,90		3,57		1,39	28,03			1,80	11,56
YP_003579465	trxA2	thioredoxin	16,61	9,96	13,06	9,08	16,97	10,71	13,79	6,12	12,46	9,58	12,55	10,62	12,51	1,28	15,03	5,74
YP_003579466	xthA2	exodeoxyribonuclease III					1,88	6,23	1,68				2,90	8,86			2,87	
YP_003579471	rcc03340	hypothetical protein													0,87		1,45	
YP_003579473	nrdD	anaerobic ribonucleoside-triphospha															2,36	
YP_003579483	metH2	methionine synthase subunit B													2,18	4,19	2,35	25,86
YP_003579485	cobQ2	cobyric acid synthase CobQ			1,39								3,47				11,78	
YP_003579486	cobQ3	cobyric acid synthase CobQ	2,90	27,68	3,00						2,64		2,62		3,54	27,34	4,15	
YP_003579490	rcc03359	iron siderophore/cobalamin ABC tra	5,27	6,62	6,87		5,01	7,27			7,88		8,94		9,85		12,62	
YP_003579494	bioB	biotin synthase													2,82	31,34	2,68	
YP_003579496	bioD	dethiobiotin synthase			0,46		0,86				0,44				0,96		0,37	
YP_003579507	rcc03376	hypothetical protein	6,43		14,92	18,89	9,80	19,55	8,02	15,06	12,98	5,66	14,74	16,77			5,11	43,29
YP_003579512	sseA	3-mercaptopyruvate sulfurtransferas	7,40	9,48	7,85	22,65	8,56	23,04	9,32	13,04	7,40	29,22	7,66	5,92	8,95	18,45	10,48	19,12
YP_003579513	tyrB	aromatic-amino-acid aminotransfera	17,90	16,57	17,23	15,31	15,19	20,82	24,09	42,05	14,71	17,23	28,99	26,46	23,11	9,37	19,61	15,00

YP_003579518	glnB2	nitrogen regulatory protein P-II	264,00	8,04	108,14	13,56	270,91	11,50	201,78	11,65	182,65	18,08	80,29	4,58	111,34	18,79	63,92	7,05
YP_003579520	rcc03389	toluene tolerance family protein	6,95	3,61	8,56	16,01	6,72	23,67	10,37	11,88	6,98	5,67	9,55	10,79	8,12	14,75	10,22	21,69
YP_003579521	rcc03390	VacJ family lipoprotein	0,99		1,03	24,98	0,94				0,20							
YP_003579523	rcc03392	hemolysin D							0,76									
YP_003579524	rcc03393	lytic murein transglycosylase							1,20									
YP_003579528	def2	peptide deformylase	2,03	3,11	2,49	10,67	3,00	13,69	2,22	5,58	2,98	15,42	2,05	3,11	1,81		3,56	3,68
YP_003579529	malY	cystathionine beta-lyase			0,60		6,34	6,44	6,79	17,94	2,25							
YP_003579532	rcc03401	band 7 protein family	1,67	15,76	1,53	25,27	1,41	25,99	0,91	29,64	0,95		1,24		1,23	4,98	1,54	18,60
YP_003579535	pyrF	orotidine 5'-phosphate decarboxylas	3,70	21,27	4,50		3,85	21,07	5,19	4,05	3,83	30,20	3,61	26,15	3,61	24,81	3,42	
YP_003579537	clpB	chaperone ClpB	4,98	1,29	2,73	0,43	13,03	32,45			2,97	22,70	2,83	7,16	2,89	3,94	5,63	61,89
YP_003579543	thcD	rhodocoxin reductase			0,73				1,00				1,11					
YP_003579544	rcc03413	peroxiredoxin	28,30	12,70	31,30	14,93	43,83	22,17	37,33	7,80	36,68	23,49	38,74	9,49	18,80	4,10	23,64	2,53
YP_003579545	rcc03414	hypothetical protein											1,53					
YP_003579549	smc	chromosome partition protein Smc											3,72	8,32				
YP_003579554	ispH	4-hydroxy-3-methylbut-2-enyl dipho	6,90	8,71	5,64	15,59	7,37	5,32	6,71	10,13	7,08	10,61	7,55	18,06	6,19	22,91	6,68	6,06
YP_003579555	rcc03424	winged helix family two component							3,33									
YP_003579562	livK2	branched-chain amino acid ABC tran	9,28	7,38	5,70	16,82	2,90		2,79	11,37	4,93	13,07	8,72	8,85	2,83	5,20	5,24	15,19
YP_003579565	map	methionine aminopeptidase	3,50	15,76	2,96	15,91	2,54	27,57	3,94	11,58	2,30	3,35	4,69	15,16	3,09	29,44	4,01	13,61
YP_003579566	rcc03435	molybdopterin binding domain-conta			4,26		4,43	9,44	4,61	1,30	3,95	17,17	5,16	13,95	4,50		4,04	9,22
YP_003579567	rcc03436	GNAT family acetyltransferase							1,53		0,67		1,92	71,23	1,35		1,44	38,45
YP_003579568	rcc03437	OmpA/MotB domain-containing prot	1,07				0,56	6,63	1,29		0,32				0,81			
YP_003579570	fadH	2_4-dienoyl-CoA reductase											1,11				1,84	
YP_003579575	nagA	N-acetylglucosamine-6-phosphate de							1,04									
YP_003579576	serB	phosphoserine phosphatase SerB			1,96		2,13		2,72	12,28	2,34		2,48	1,47			1,96	
YP_003579577	serC	phosphoserine aminotransferase	11,37	3,26	9,55	20,50	11,09	8,40	14,28	11,70	9,99	30,51	10,80	4,86	9,28	32,18	12,15	19,11
YP_003579578	serA	phosphoglycerate dehydrogenase	12,27	13,59	11,39	15,76	12,24	13,04	16,09	15,06	10,98	24,04	13,83	0,88	11,06	13,67	13,40	13,66
YP_003579580	atoB3	acetyl-CoA acetyltransferase	2,59	6,30	4,02	22,97	2,31	26,53	3,81	14,82	2,18	33,02	2,70	14,52			1,72	21,49
YP_003579582	rcc03451	alpha-2-macroglobulin domain-conta	8,49	17,28	6,33	10,78	8,50	13,95	8,94	10,53	6,06	15,54	6,61	0,95	5,04	7,00	5,56	20,84
YP_003579588	mepA	penicillin-insensitive murein endope											1,03					
YP_003579592	ispA	geranyltranstransferase	3,76	21,74	3,53	5,52	3,80	22,02	4,63	5,97	3,00	25,35	3,03	14,52	2,97	20,82	3,74	8,66
YP_003579593	dxs2	1-deoxy-D-xylulose-5-phosphate syn							1,61									
YP_003579597	rcc03466	hypothetical protein			7,06													
YP_003579604	gabT2	4-aminobutyrate aminotransferase							0,85								0,96	
YP_003579608	msrB2	peptide-methionine (R)-S-oxide redu	0,68															
YP_003579609	msrA2	peptide-methionine-(S)-S-oxide redu	3,64	1,37	4,41	2,06	4,43	5,87	3,87	18,42	6,55	67,20	5,62	1,29	4,57		3,69	4,60
YP_003579619	hemS	hemin transport protein HmuS					1,50		1,58	0,60	1,54		0,66		1,17		1,48	
YP_003579621	rcc03490	endoribonuclease_ L-PSP family	3,46	1,77	2,95	6,13	3,79		2,15				4,47	32,18	2,68			
YP_003579622	rcc03491	nucleoside-triphosphatase	4,28	22,89	4,24	19,26	4,88	23,62	2,72	7,16	4,07	26,05	3,13	17,72	2,74	13,73	2,93	28,72

YP_003579623	rph	ribonuclease PH	9,02	7,99	7,68	13,34	9,64	19,74	11,73	9,69	7,32	21,79	9,50	2,96	7,61	15,60	8,79	9,97
YP_003579624	hrcA	heat-inducible transcription represso													0,51			
YP_003579625	grpE	GrpE protein	20,17	6,35	14,77	24,97	21,34	24,58	9,79	29,51	19,08	16,21	10,72	1,82	5,38	5,39	9,90	21,90
YP_003579626	mutS	DNA mismatch repair protein MutS					1,26										1,07	
YP_003579627	maeB2	malate dehydrogenase	8,25	4,70	5,57	26,85	5,94	13,02	7,26	9,76	5,44	24,17	6,29	13,92	5,63	23,16	7,20	19,65
YP_003579628	rbsK	ribokinase	4,06	11,80	2,60	6,70	1,72	0,44	1,66	34,92	2,59	28,37	2,10	7,87	2,18		3,35	
YP_003579629	phoB	phosphate regulon transcriptional re			5,55													
YP_003579630	phoU	phosphate transport system regulato	8,40	10,94	5,32	5,83	6,10	41,01	4,14	18,11	4,70	33,03	4,31	27,24	6,52	25,08	9,48	34,91
YP_003579631	pstB	phosphate ABC transporter ATP-bind							1,86	26,33	1,59		2,76		2,05	29,79	2,81	
YP_003579634	pstS	phosphate ABC transporter periplasr	76,83	22,86	64,08	30,25	69,62	10,21	64,28	11,02	98,62	44,85	45,32	34,89	91,63	8,84	118,10	11,44
YP_003579635	cbiX	cobalamin biosynthesis protein CbiX			0,58				0,84	2,53	0,74		1,25		1,13			
YP_003579641	rcc03510	hypothetical protein	18,72	11,61	16,23	1,70	14,88	21,06	12,04	19,75	11,12	15,41	8,30	37,10	10,08	12,43	9,81	14,41
YP_003579642	rcc03511	HemY domain-containing protein	10,08	10,27	7,34	26,53	9,41	29,05	6,97	13,84	4,54	43,70	6,29		4,82	0,55	5,66	26,29
YP_003579645	flgA	flagella basal body P-ring formation p													1,23		0,90	
YP_003579647	flgF	flagellar basal-body rod protein FlgF	0,25															
YP_003579656	flaA	flagellin protein	3,39	20,32			5,74	27,28	4,15	16,17	3,21	15,11			6,13	11,35	5,13	30,53
YP_003579662	ubiE	ubiquinone/menaquinone biosynthe	4,04	34,36	4,55	23,43	6,19	1,50	4,71	14,01	4,06	20,69	5,78	24,58	5,95	21,74	8,36	21,03
YP_003579664	paaF	enoyl-CoA hydratase	7,22	11,65	6,66	20,59	7,40	17,05	10,35	13,28	6,13	24,50	8,04	4,62	4,65	15,34	5,98	10,21
YP_003579665	rpsT	30S ribosomal protein S20															49,27	
YP_003579688	rcp00023	NAD-dependent epimerase/dehydrat	4,57															
YP_003579709	rcp00044	iron siderophore/cobalamin ABC trar															0,18	
YP_003579754	rcp00091	OLD family nuclease															0,88	
YP_003579756	rcp00093	short-chain dehydrogenase/reductas															9,63	
YP_003579789	mdcE	malonate decarboxylase subunit gam	3,53															
YP_003579804	rcp00142	hypothetical protein			0,48													
YP_003579810	rcp00150	polyols ABC transporter ATP-binding															0,41	
YP_003579813	rcp00153	sugar isomerase domain-containing p					0,55											
